# Base gap switches as transcriptional regulators and their applications as biosensors

**DOI:** 10.1038/s44460-026-00134-z

**Published:** 2026-09-14

**Authors:** Yukina Partington, Beatrice Dalla Via, Fabrice Abgottspon, Isaure de Geyer, Piet Cools, Andrew J. deMello, Daniel A. Richards

**Affiliations:** 1 Institute for Chemical and Bioengineering, ETH Zurich, Zürich, Switzerland; 2 Department of Diagnostic Sciences, Faculty of Medicine and Health Sciences, Ghent University, Ghent, Belgium

**Keywords:** Assay systems, Diagnosis

## Abstract

Transcriptional and translational regulators (‘switches’) function as biosensors by controlling how gene circuits respond to specific stimuli. However, contemporary switches suffer from spontaneous activation or off-target responses, limiting sensitivity and specificity. Here we introduce base gap switches, a class of programmable transcriptional regulators that are selectively activated by DNA. Base gap switches are simple to design and install, provide tight control of gene expression and exhibit high specificity and orthogonality. They can discriminate single-nucleotide mismatches and integrate with nucleic acid amplification methods. Using base gap switches, we developed an assay for *Trichomonas vaginalis* with a limit of detection of 0.6 copies μl^−1^ and validated its performance using clinical samples. We further demonstrate a one-pot assay for differentiating *Neisseria gonorrhoeae* and *Chlamydia trachomatis*, and an assay for detecting single-nucleotide variants of *EGFR*-associated cell-free DNA. These results establish base gap switches as a versatile tool for gene circuit-based biosensing.

## Abstract (web summary)

Base gap switches provide a programmable platform for DNA-responsive gene circuit biosensing, enabling single-nucleotide discrimination and sensitive detection of pathogens and cancer-associated DNA variants.

## Main

Owing to their ability to control transcription and translation, synthetic gene circuits (SGCs) have become indispensable tools for synthetic biologists^1–3^. As biosensors, they are routinely employed to detect nucleic acids^4–6^, proteins^7^ and small molecules^8–10^, and are also used within biological computing^11–14^, drug discovery^3,15,16^ and plant biology^17,18^. Fundamental to the function of SGCs are transcriptional and translational regulators (‘switches’), which modulate the expression of proteins (‘outputs’) in response to specific analytes (‘inputs’). A key feature of these switches is their programmability, which enables inputs and outputs to be adapted to specific applications. Unfortunately, contemporary switches often suffer from a propensity to express protein in the absence of the target analyte (‘leakage’) or presence of unrelated targets (‘crosstalk’)^19–22^. This fundamentally limits the sensitivity, specificity and orthogonality of any resulting biosensor. To circumvent these limitations, we introduce ‘base gap’ switches, a switch design that exhibits minimal leakage, high specificity and low crosstalk.

The most well-studied class of switches are translational riboregulators. Toehold switches, first introduced by Green et al.^4^, are arguably the most ubiquitous example. Combined with in vitro transcription/translation (IVTT), toehold switches have been extensively employed as disease biosensors, including within logic-gated sensors and wearables^2,5,6,23–28^. Conversely, transcription-based switches for in vitro biosensing are comparatively rare. Phillips et al. developed the internal splint-pairing expression-cassette ligase-mediated translation reaction (INSPECTR) assay, which leverages ‘splint pairing’ of genes to induce protein expression^29^. INSPECTR achieves excellent sensitivity, although programming target-specificity into these circuits requires altering the protein coding sequence, potentially impacting expression yields or protein function. Thus, the exact position of the alteration must be optimized for each new target and reporter protein combination. Hu et al. reported a hybridization-based transcriptional switch, although the design restricts actuation to promoter-containing sequences and the system was never coupled to translation^30^. Park et al. introduced the target-assisted synthesis of enzymatic reporters (TASER) method, which employs DNA-directed polymerase strand repair to activate gene circuits^31^. This elegant approach simplifies switch design but suffers from high leakage. Kim et al. reported a split T7 RNA polymerase (RNAP) switch that exploits target-dependent sealing of nicks in a T7 promoter to control transcription, and subsequently, translation^32^. However, the system similarly suffers from high leakage and poor sensitivity. These split T7 switches have also been used effectively in translation-based biosensors in which target presence initiates the transcription of RNA aptamers^33,34^. Despite their limitations, these systems demonstrate that transcriptional switches could be a viable alternative to traditional riboswitches.

**Fig. 1:**
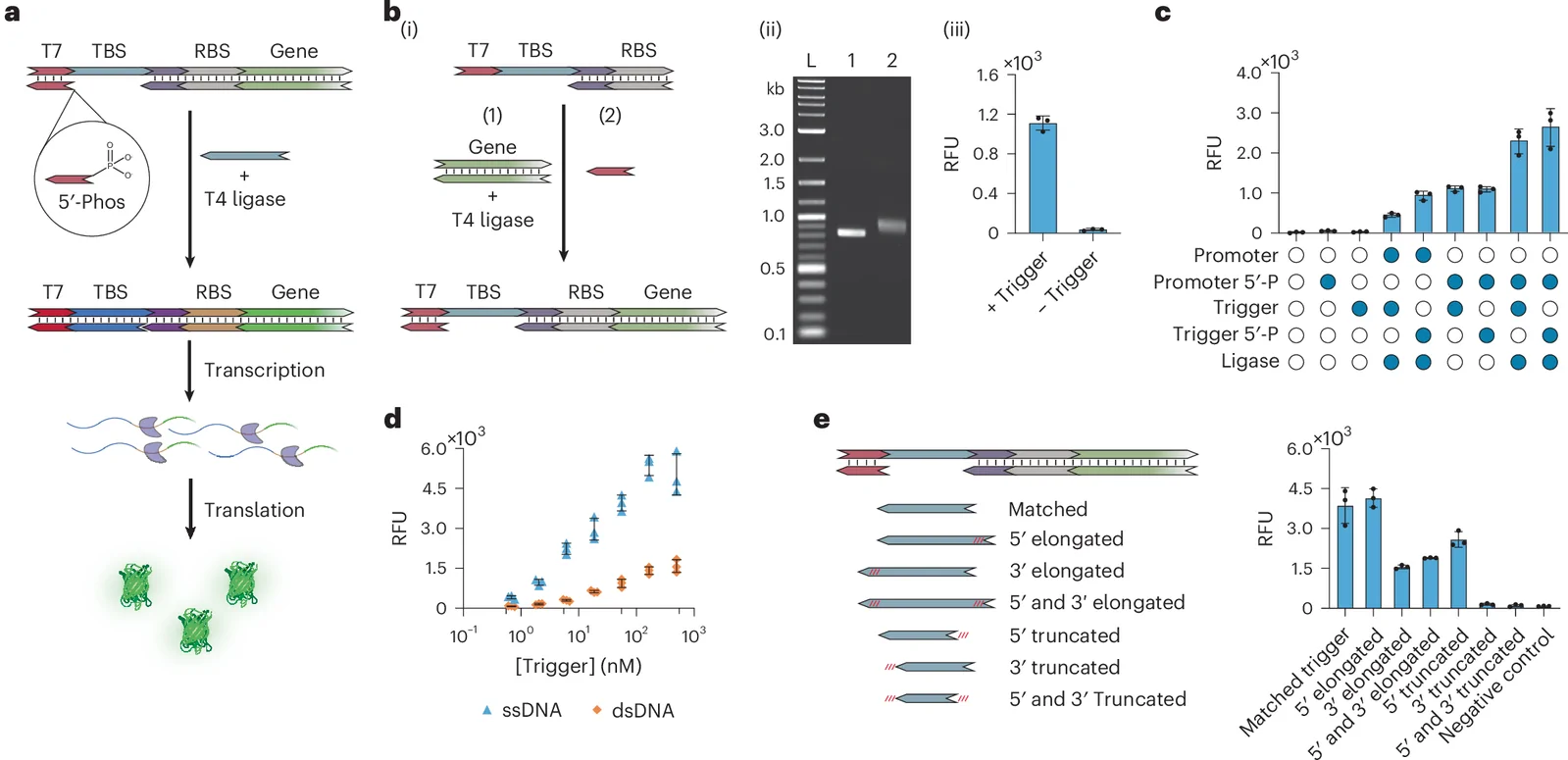
Base gap synthesis and initial validation. **a**, A schematic showing the signalling cascade controlled by base gap switches. A gap, specific to a particular target sequence, prevents protein expression. Binding and ligation of the complementary ‘trigger’ enables transcription by RNAP, followed by translation to produce the protein encoded by the gene. **b**, (i) The ligase-mediated protocol for installing base gap switches onto a gene. (ii) An agarose gel showing a base gap switch attached to an mNeonGreen gene. ‘L’ is the DNA ladder, lane 1 is the native mNeonGreen gene and lane 2 is the mNeonGreen gene with the base gap switch. Successful attachment of the switch is shown by the DNA band shift. (iii) mNeonGreen fluorescence from a base gap switch-controlled gene. Expression only occurs in the presence of the trigger. **c**, mNeonGreen fluorescence under ligating and non-ligating conditions. Ligation between the trigger and the promoter region enhances protein expression. Ligation between the trigger and the RBS region does not alter protein expression. **d**, mNeonGreen fluorescence from a base gap switch-controlled SGC triggered by either ssDNA or dsDNA. Both can actuate the base gap switch. **e**, The relative mNeonGreen fluorescence when using length-mismatched triggers. Three base elongations are tolerated at both the 5′ and 3′ ends of the trigger. However, three base truncations are only tolerated at the 5′ end. All plots with error bars represent the mean of three measurements (*n* = 3) ±1 standard deviation. Source data

Here, we report base gap switches, a class of transcriptional switch that exploits gaps in genes to modulate protein expression (Fig. 1a). In the absence of the target DNA, RNAP is unable to traverse the gap on the template strand, and transcription of the protein-encoding gene is prevented. When present, the cognate target DNA fills this gap, facilitating transcription and translation. Base gap switches can be programmed to respond to specific DNA triggers, operate orthogonally, exhibit low leakage and can control the expression of various functionally distinct proteins. Using base gap switches, we construct three model assays: first, an ultrasensitive assay for *Trichomonas vaginalis* (TV). Second, a one-pot assay for differentiating *Chlamydia trachomatis* (CT) and *Neisseria gonorrhoeae* (NG), and third, an assay for detecting a single-nucleotide variant (SNV) in cell-free DNA (cfDNA) associated with an epidermal growth factor receptor (*EGFR*) mutation. These assays demonstrate how the unique features of base gap switches can be leveraged to develop functional biosensing assays.

## Results

### Base gap switches

Base gap switches comprise three essential features (Fig. 1a). First, the target binding site (TBS), a single-stranded sequence where the target DNA binds. Second, a promoter site to enable binding of RNAP and subsequent transcription. Finally, a ribosome binding site (RBS) to facilitate translation. Using these principles, we designed a base gap switch with a 50-nucleotide TBS and attached it to an SGC coding for mNeonGreen (Fig. 1b). This SGC was generated by ligating the base gap switch to an mNeonGreen-encoding gene (Fig. 1b(i),(ii) and Supplementary Fig. 1). Since T7 RNAP can actively transcribe single-stranded templates^35,36^, leading to increased leakage (Supplementary Fig. 2), we installed the base gap into the noncoding strand to ensure disruption of the transcription template and reduce leakage.

As a proof-of-concept, we measured the expression of mNeonGreen from this base gap switch-controlled gene and observed target-dependent expression (Fig. 1b(iii)). Notably, expression in the presence of the target was lower than in an unmodified control gene, which we attributed to nicks in the template strand (Supplementary Fig. 3). These nicks are not completely deleterious to the function of the switch. We attribute this to the template-switching ability of RNAP, which enables it to traverse, or ‘jump’, nicks and gaps in the template strand^37^. This could be mitigated by ligating the nick between the T7 promoter and the TBS (Fig. 1c, column 8). Intriguingly, the presence of a nick on the distal end of the trigger–switch interface, between the TBS and the RBS, did not impact expression, and sealing this nick is not necessary (Fig. 1c, column 9). Extended pre-incubation with T4 ligase did not substantially improve expression yields (Supplementary Fig. 4(i)), nor did increasing the ligase concentration (Supplementary Fig. 4(ii)). Accordingly, moving forwards, we performed ligation alongside IVTT (‘one pot’ ligation/IVTT) to reduce the number of assay steps. These optimized base gap switch-controlled genes remain stable at both 21 °C and 4 °C for at least 8 weeks (Supplementary Fig. 5), although it is worth noting that other components (PURExpress and Quick Ligase) were stored at –20 °C until use. We are conducting further experiments to determine the stability of lyophilized mixtures.

Next, we evaluated base gap switch actuation by double-stranded DNA (dsDNA). We incubated a base gap switch-controlled SGC with complementary single-stranded (ssDNA) and dsDNA targets. In the case of the double-stranded target, an excess of the non-target complement was employed to ensure no complementary ssDNA remained. Concentration-dependent mNeonGreen expression was observed in both cases, although it was reduced for the dsDNA target (Fig. 1d). We hypothesize that the TBS sites of base gap switches can engage in strand displacement with dsDNA targets, thus facilitating hybridization with their cognate ssDNA. Since DNA is primarily double-stranded, this result bolsters the utility of base gap switches.

Finally, we studied how length mismatches between a switch and its trigger impact protein expression (Fig. 1e). Truncation from the 3′ terminus completely muted protein expression. This was expected, given the previously established necessity of ligation between the promoter region and the trigger DNA. Interestingly, extensions to the 3′ terminus were better tolerated, suggesting that ligation between the trigger and switch is possible even with non-optimal base pairing. Truncation at the 5′ terminus had less of an impact on mNeonGreen expression, suggesting that RNAP can bypass gaps at this position. Intriguingly, extension at the 5′ terminus did not impact expression yields. We attribute this to the aforementioned template switching^37^, which enables RNAP to jump from the mismatched template onto the template encoding for mNeonGreen. Extensions of up to 24 and 6 bases are tolerated at the 5′ and 3′ termini, respectively, though expression yields decrease as a function of extension length (Supplementary Fig. 6).

### Structure–function relationships

**Fig. 2:**
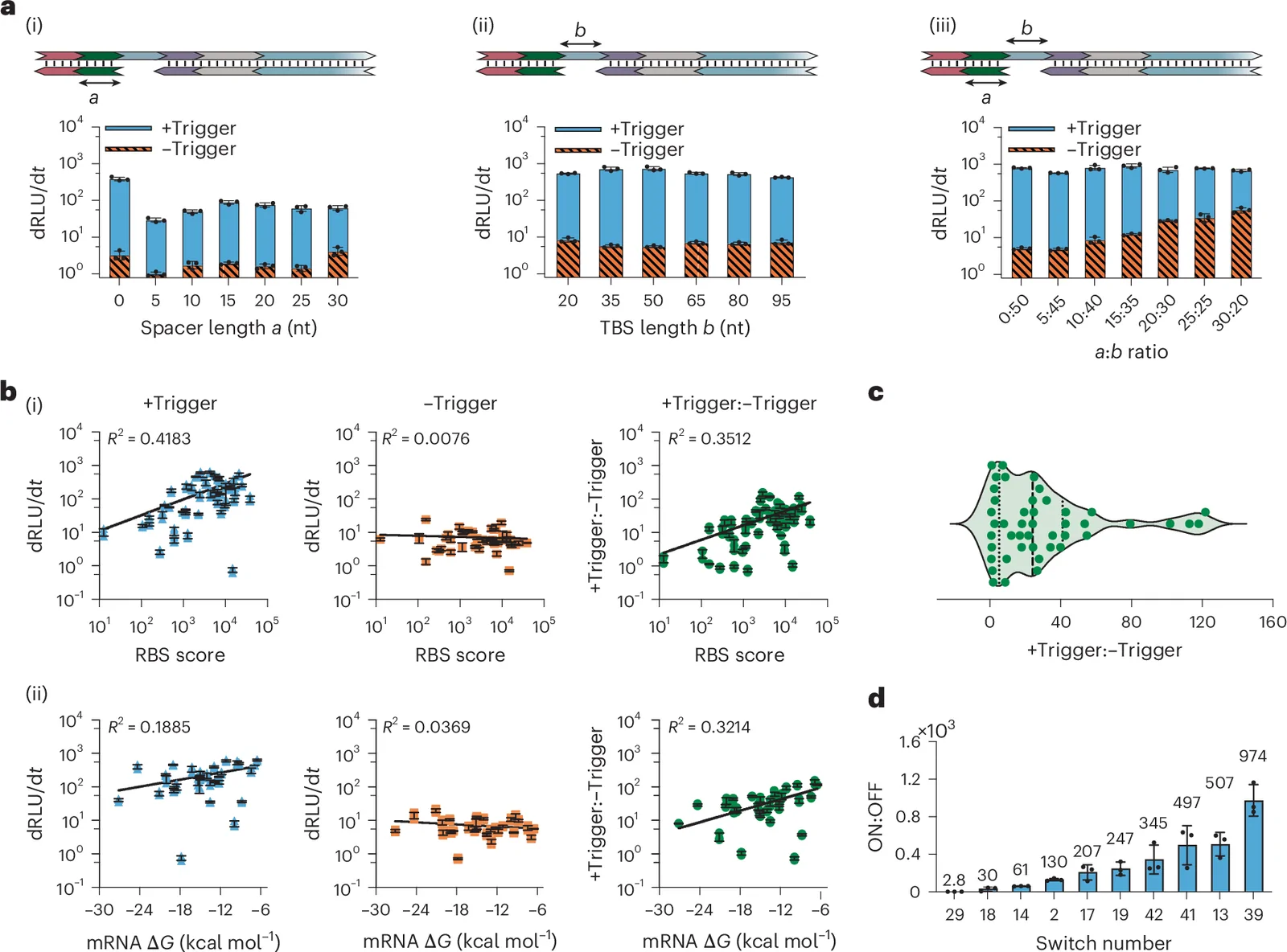
Structure–function relationships. **a**, Nluc expression as a function of (i) the number of basepairs between the promoter and the TBS (*a*), (ii) the TBS length (*b*) and (iii) the ratio of the the promoter–TBS gap length and the TBS length (*a*:*b*). A gap of five basepairs is optimal, combined with a high *a*:*b*, although expression yields are invariable to TBS length. **b**, Nluc expression from 45 base gap-switch-controlled SGCs with varying TBS compositions as a function of (i) RBS score and (ii) the Gibbs free energy of formation for mRNA hairpins. The y-axis indicates the maximum slope of the first derivative curve of the relative luminescence units (RLU) as a function of time. Data shows expression in the presence (‘+Trigger’, blue triangles) and absence (‘−Trigger’, orange squares) of triggers, as well as the ratio of the two (‘+Trigger:−Trigger’, green circles). Switch performance is positively correlated with increasing RBS scores and decreasing hairpin stability. Switches with an RBS score <1000 were not included in subsequent analyses. **c**, A violin plot of the +Trigger:−Trigger values. **d**, mNeonGreen expression from a selection of switches, from low to high (‘ON:OFF’). The numbers above the bars represent the ON:OFF ratio. All plots with error bars represent the mean of three measurements (*n* = 3) ±1 standard deviation. The *R*^2^ values were obtained from a semi-log fit of the data. Source data

To inform the rational design of base gap switches, we explored how their structure impacts their ability to control expression (Fig. 2). Given the low levels of leakage observed with base gap switch-controlled mNeonGreen genes, we performed these studies using a NanoLuc Luciferase (Nluc) reporter to ensure an observable signal.

We first optimized the spacer between the T7 promoter and the TBS. To minimize any bias in relative expression rates^35^, we kept the five bases (GGGCC)^38^ immediately after the promoter and the composition of the TBS constant (Fig. 2a(i)). We achieved the best performance with a five basepair spacer and observed no apparent relationship between spacer length and expression levels. Using this optimized spacer, we varied the TBS length, extending in the middle to avoid altering the composition near the sensitive promoter–TBS or TBS–RBS junctions (Fig. 2a(ii)). No meaningful relationship between TBS length and expression levels was apparent, suggesting base gap switches can detect targets of various lengths. Increasing the length of the spacer while decreasing the length of the TBS led to an increase in switch leakage (‘−Trigger’) (Fig. 2a(iii)). This suggests that when short TBS lengths are employed, shorter spacers are necessary to decrease leakage and improve performance.

To better guide the selection of TBSs, we created a library of 50 nt base gap switches with varied TBS compositions and monitored their control over Nluc expression when activated by ssDNA triggers (Fig. 2b,c). We correlated the performance of these switches against multiple structural and thermodynamic parameters (Supplementary Fig. 7), ultimately finding the best predictors of performance were RBS Score (*R*^2^ = 0.4183) (Fig. 2b(i)), calculated using the Salis Lab RBS calculator^39,40^, and the Gibbs free energy of mRNA hairpin formation (*R*^2^ = 0.3214) (Fig. 2b(ii)). Interestingly, we observed no discernible trends when studying expression in the absence of trigger (‘−Trigger’). Furthermore, we found no correlation between switch performance and relative promoter strength, Gibbs free energy of trigger–TBS complementation or basepair composition at the ligation or RBS junctions. The lack of correlation with basepair composition is particularly noteworthy, since prior reports suggest that the capacity of T7 RNAP to traverse gaps or nicks, is highly sequence-dependent^37^. This suggests that the differences in expression control across base gap switches are largely attributable to their relative impacts on translation, not transcription. This concurs with previous findings that translation is the rate-limiting step in IVTT^41^, and has a greater impact on relative protein expression^42^.

Owing to the rapid conversion of the furimazine substrate at high trigger concentrations, causing signal saturation, we were unable to determine true ON:OFF ratios for the base gap switches in the Nluc library. To address this, we selected ten switches, ranging between low-performing and high-performing, and installed them onto an mNeonGreen gene. These switches exerted strong control over mNeonGreen expression, with ON:OFF ratios ranging from 2.8 ± 0.25 to 973 ± 167 (Fig. 2d). The highest of these ratios are substantially greater than toehold switches coupled to green fluorescent protein reporters, which have not exceeded ~150:1 in IVTT systems^6^, and typically remain below 50^4–6^. Only a weak positive correlation was observed between switch performance on Nluc and mNeonGreen (Supplementary Fig. 8). This is unsurprising, given that the coding DNA sequences (CDSs) of genes can impact translation rates^42^ and thus RBS scores. This indicates that the CDS of the attached genes must be considered when designing base gap switches.

### Coupling to nucleic acid amplification

**Fig. 3:**
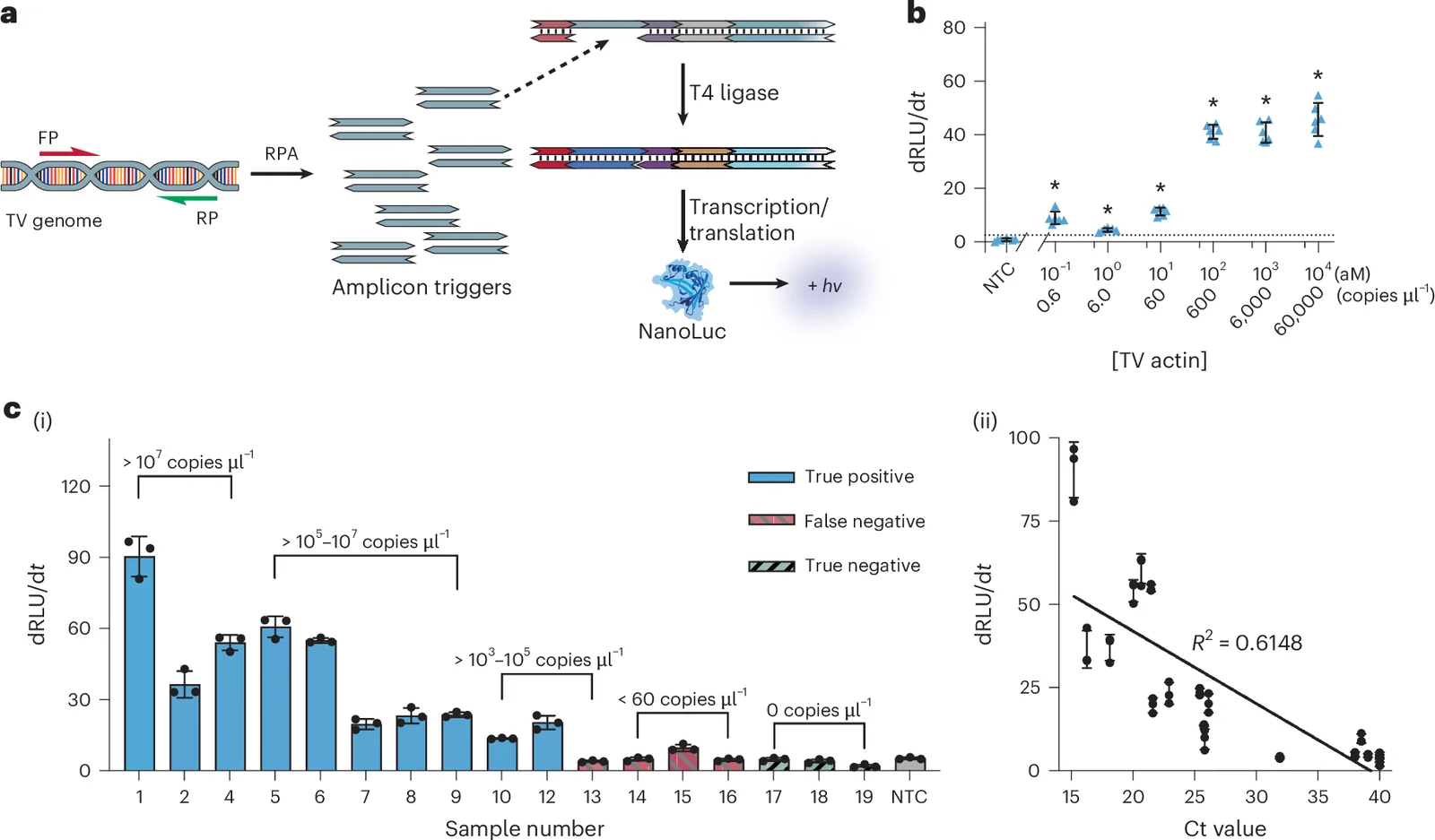
Combining base gap switch-controlled SGCs with nucleic acid amplification. **a**, A schematic representation of the two-step RPA–IVTT assay. Amplicons produced by RPA actuate the base gap switch, leading to expression of Nluc. ‘FP’ and ‘RP’ refer to the forward and reverse primers, respectively. **b**, Luminescence as a function of TV genome concentration. A positive dose–response relationship is observed, with a LoD of 0.1 aM (0.6 copies µl^−1^). Error bars represent the mean of six measurements (*n* = 6) ±1 standard deviation. Statistical significance between all positive measurements and the NTC was determined using a two-sided Mann–Whitney test (Asterisks denote significance; *P* = 0.0022). **c**, (i) The relative luminescence resulting from RPA–IVTT analysis of patient-derived samples. A comparison with the S-DiaMGTV qPCR assay (shown in ii) showed good correlation. Error bars represent the mean of three measurements (*n* = 3) ±1 standard deviation. Source data

The exponential nature of nucleic acid amplification confers unparalleled sensitivity, and primer-directed amplification enables precise control of amplicon composition to perfectly match the TBS of a base gap switch. To explore the combination of nucleic acid amplification and base gap-controlled SGCs, we designed a two-step recombinase polymerase amplification (RPA)–IVTT assay (Fig. 3a). We targeted the actin gene of *Trichomonas vaginalis* (TV), the world’s most prevalent non-viral sexually transmitted infection^43^. To maximize sensitivity, we installed our TV-specific base gap switch onto a gene encoding for Nluc. We observed dose-dependent luminescence (Fig. 3b and Supplementary Fig. 9) using genomic DNA, with a limit of detection (LoD) of 0.6 gene copies µl^−1^. No dose-dependent expression was observed when incubating the circuit with primer dimers (Supplementary Fig. 10a), ruling out non-specific amplification. While non-matched triggers containing complementarity regions at the termini can activate a base gap switch (Supplementary Fig. 10b), this is only observed with a high degree of shared identity and expression rates are reduced. Together, these experiments demonstrate that non-specific base gap switch actuation can be avoided with appropriate primer design.

We next analysed 17 cervical swab samples (Fig. 3c(i) and Supplementary Fig. 11). This selection was strategically chosen to contain high (>10^7^ copies µl^−1^), medium (10^5^–10^7^ copies µl^−1^), low (10^3^–10^5^ copies µl^−1^) and ultralow concentrations of TV (<10^2^ copies µl^−1^), as determined using a commercial quantitative PCR (qPCR) test (S-DiaMGTV, Diagenode) (Supplementary Table 6). Our assay correctly identified samples containing low-to-high TV titres, showing good agreement with the qPCR standard (Fig. 3c(ii)), although it was unable to classify samples containing fewer than 60 copies µl^−1^ of the gene. We attribute this to differences in sample volumes used during the assays (qPCR uses 5 µl of sample, our assay uses 2.5 µl), differences in copy numbers in the gene target or assay inhibition by residual matrix components. Despite the small cohort size, this study demonstrates the potential of base gap switch-controlled SGCs for highly sensitive clinical target detection.

### Base gap switch orthogonality

**Fig. 4:**
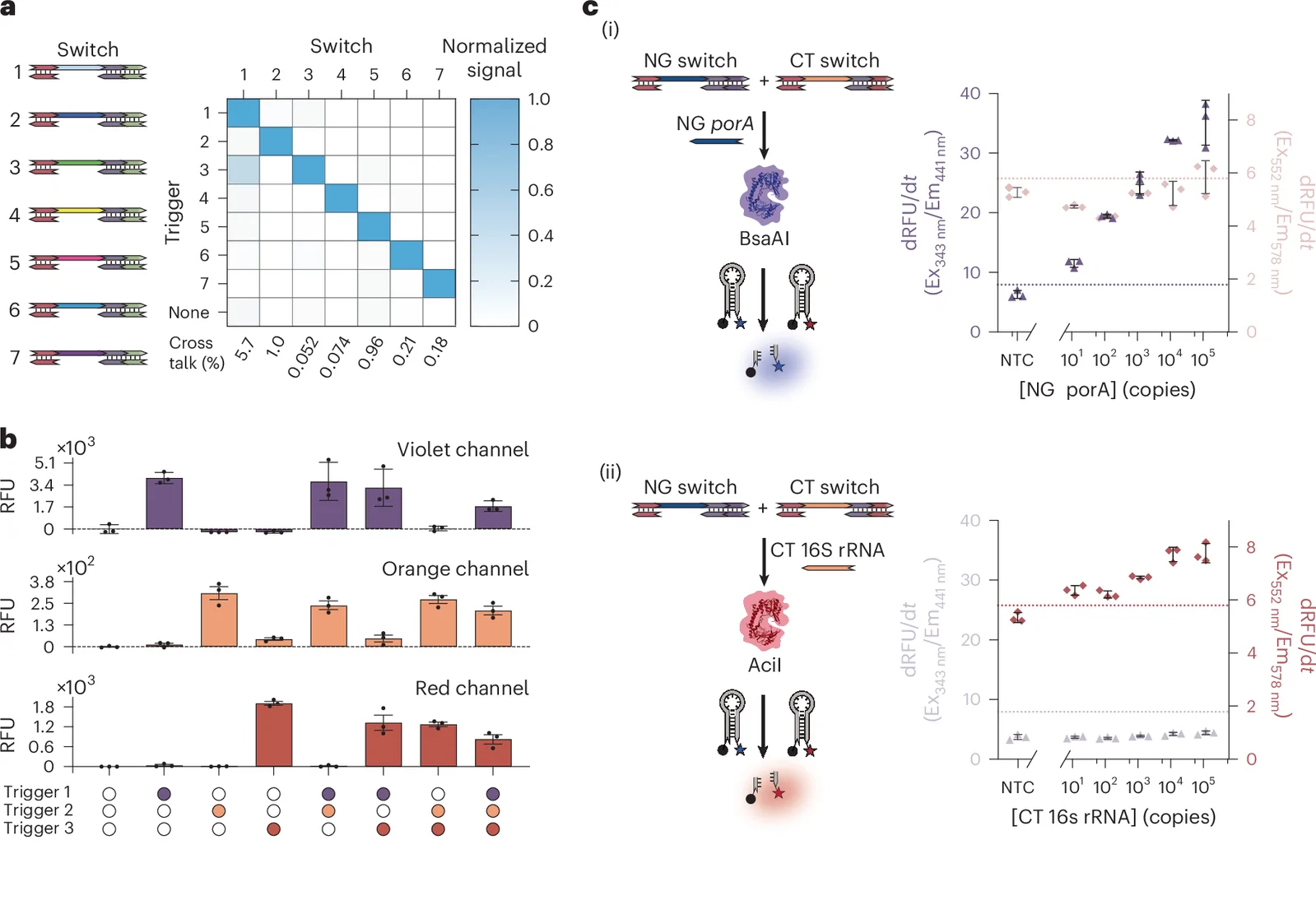
Demonstrating the orthogonality of base gap switches. **a**, The normalized mNeonGreen fluorescence from seven pairwise combinations of base gap switch-controlled genes and triggers. Data are normalized for each base gap switch and cognate pair (1, matched trigger; 0, no trigger). Crosstalk is calculated as the average relative signal obtained from actuation by all non-cognate triggers. Substantial fluorescence is only observed for cognate pairs. **b**, The relative Sirius, mPapaya and mNeptune fluorescence from a mixture of base gap-controlled genes actuated by orthogonal triggers. Signal is only observed in each channel (violet, orange and red) when the cognate trigger is present. **c**, The relative AciI and BsaAI expression as a function of NG *porA* (i) and CT 16S rRNA (ii) DNA concentration. RPA was performed before incubation with the gene circuit. Fluorescence is generated from the cleavage of quenched molecular beacons. Data for each graph were obtained from a mixture of the gene circuits in a single reaction vessel. The dotted violet and red lines represent the LoD for NG *porA* and CT 16S rRNA, respectively, defined as the mean of three negatives (no target) + two standard deviations. The base gap switches maintained their orthogonality, even at high concentrations of the non-target DNA. All plots with error bars represent the mean of three measurements (*n* = 3) ±1 standard deviation. Source data

To demonstrate the orthogonality of base gap switches, we placed an mNeonGreen gene under the control of seven orthogonal base gap switches. Cross-validation revealed excellent orthogonality (Fig. 4a), with appreciable crosstalk observed only between switches 1 and 3 (Fig. 4a and Supplementary Fig. 12a,b). This is probably caused by the intentionally high degree of sequence identity between these switches (90% similarity) (Supplementary Fig. 12c). We observed no decrease in sensitivity or selectivity when incubating a gene under the control of switch 2 with an equimolar mixture of the other triggers (Supplementary Fig. 12d). This suggests that base gap switches maintain a high degree of orthogonality, even in complex mixtures of targets.

We installed the best-performing switches (5, 6 and 7) onto genes encoding for the fluorescent proteins Sirius, mPapaya and mNeptune, and determined the relative levels of protein expression in a single tube (Fig. 4b). In each case, orthogonality was maintained. We attribute the small signal in the orange channel when expressing mNeptune to signal bleed-through when exciting the protein at 510 nm (the mPapaya excitation wavelength) (Supplementary Fig. 13).

To demonstrate the clinical potential of this orthogonality, we placed genes encoding for the restriction enzymes BsaAI and AciI under the control of base gap switches specific to *Neisseria gonorrhoeae* (NG) and *Chlamydia trachomatis* (CT). NG and CT are responsible for an estimated 82.4 and 128.5 million annual global infections, respectively^44^. Furthermore, NG and CT infections typically present with similar physical symptoms^45^. Accordingly, they offer a perfect use case for orthogonal, multiplexed biosensing. We used RPA to amplify gene sections corresponding to the Porin protein A (*porA*) pseudogene for NG and the 16S ribosomal RNA (16S rRNA) gene in CT^46,47^. Incubation of the resulting amplicons with the SGC mixture resulted in selective expression of the encoded restriction enzymes. (Fig. 4c(i),(ii) and Supplementary Fig. 14). We observed a reduced dynamic range in the assay for CT 16S rRNA, which we attribute to leakage from the base gap switch controlling AciI expression. Despite this, we were still able to detect 12 copies µl^−1^ of both targets and observed no crosstalk. These data demonstrate the potential of base gap switches for multiplexed biosensing.

### SNV mismatch detection

**Fig. 5:**
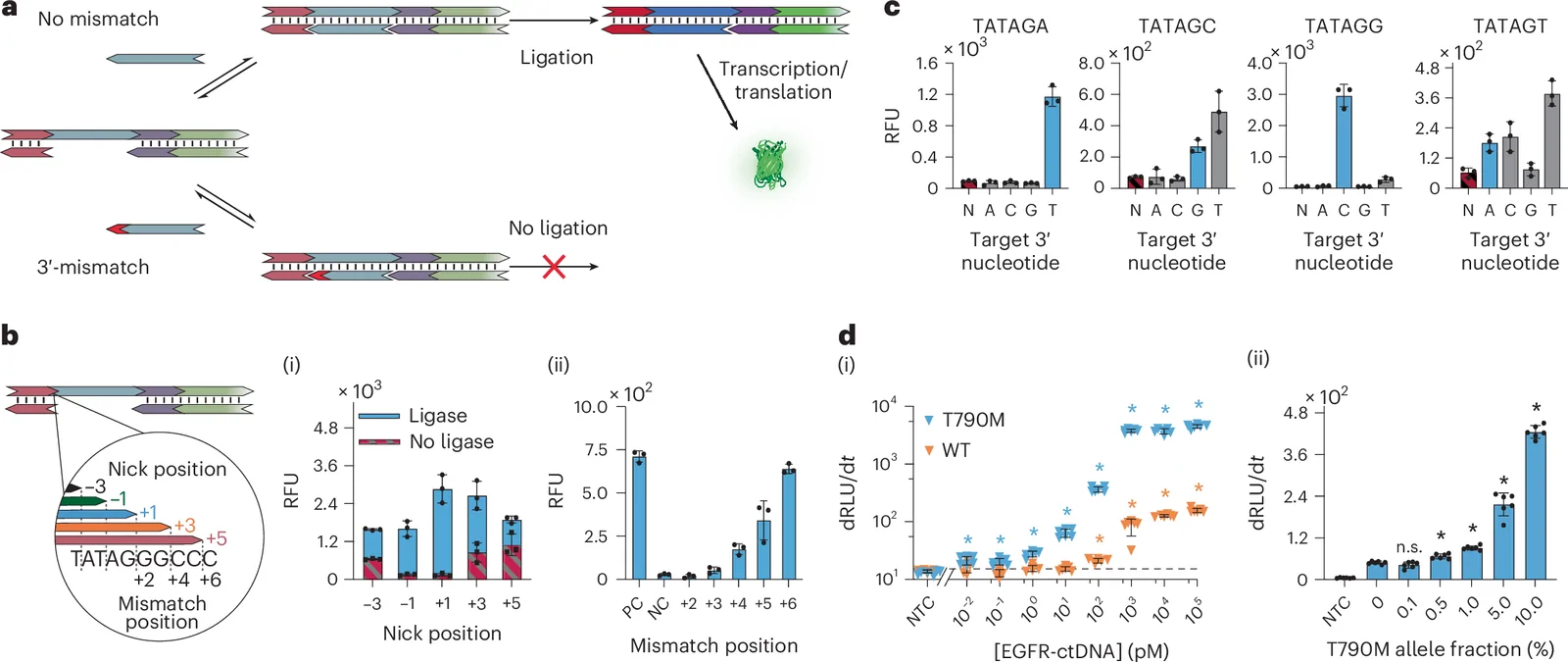
SNV detection using base gap switches. **a**, A schematic describing base mismatch discrimination using base gap switches. Mismatches at the 3′ end of the target prevents ligation to the promoter region (red). In the absence of ligation, RNAP cannot bypass the base gap and the expression cascade is muted. **b**, (i) mNeonGreen fluorescence as a function of ligation (nick) position, with and without ligase. The positions are numbered relative to the transcriptional start position for the T7 promoter (G,+1). (ii) mNeonGreen fluorescence as a function of mismatch position. A polyguanine (GGGGG) region was introduced directly following the nick site, with triggers containing a T mismatch in the assigned position. The positive control (PC) is a fully matched trigger. The negative control (NC) is no trigger. The greatest ligase dependence was observed with the nick after the +1 position, and high mismatch fidelity was observed with the mismatch placed in the +2 or +3 positions. Error bars represent the mean of three measurements (*n* = 3) ±1 standard deviation. **c**, The relative mNeonGreen fluorescence values obtained with different basepair mismatches between the switch and the trigger. The letters above indicate the nucleotide at the +2 position of the base gap switch. The *x* axis indicates the nucleotide at the 3′ end of the trigger. ‘N’ represents ‘negative’ (no target). For each graph, the bar representing the complementary base is highlighted in blue. Switches with A and G nucleotides at the +2 position were highly sensitive to basepair mismatches. Error bars represent the mean of three measurements (*n* = 3) ±1 standard deviation. **d**, (i) Nluc luminescence as a function of mutant *EGFR* cfDNA concentration. The base gap switch was specific to the T790M (C–T) mutation of *EGFR*. Overall, 10 fM of T790M DNA could be detected, whereas 100 pM of WT DNA was required to achieve significance. The dotted line represents the +2 standard deviation above the mean of the non-template control (negative). (ii) Relative Nluc luminescence as a function of T790M allele fraction (fraction of T790M cfDNA spiked into WT cfDNA). The total DNA trigger concentration was 1 nM. We detected allele fractions as low as 0.5%. Error bars represent the mean of six measurements (*n* = 6) ±1 standard deviation. Statistical significance between all positive measurements and the NTC was determined using a two-sided Mann–Whitney test (asterisks denote significance; *P* = 0.0022. n.s., not significant; *P* = 0.1797). All data were obtained using synthetic analogues of the mutants described by Ma et al.^56^. Source data

Inspired by ligase-mediated amplification methods, such as the ligase detection reaction and ligase chain reaction^48–50^, we hypothesized that the ligase dependence of base gap switches could be exploited to detect SNVs (Fig. 5a). To begin, we explored how the position of the ligation junction, controlled by the position of the nick on the template strand, impacts expression with and without ligase (Fig. 5b(i)). We observed the greatest difference in relative protein expression with and without ligation when the nick was introduced after the +1 position. Furthermore, we observed that the muting capacity of G–T mismatches decreases as they move further from the ligation junction (Fig. 5b(ii)). Accordingly, optimum mismatch discrimination is obtained with a nick after the +1 position, with the mismatch in the adjacent +2 position.

Next, we designed four base gap switches differentiated only by the nucleotide at the +2 position and incubated each with the four cognate triggers (Fig. 5c). With adenosine (A) and guanine (G) nucleotides at the +2 position, protein expression only occurred upon incubation with the cognate triggers. The same fidelity was not observed with cytosine (C) and thymine (T) nucleotides. Importantly, a ‘high-fidelity’ ligase (Taq DNA ligase) was essential for specificity; the lower fidelity T4 ligase granted no selectivity (Supplementary Fig. 15). This supports previous reports of promiscuous ligation with T4 ligase^51,52^. Single basepair mismatches at other positions within the TBS did not meaningfully impact protein expression (Supplementary Fig. 16). This suggests that selective ligation is important for mismatch discrimination. Notably, we observed that protein expression is reduced when cytosine and thymine residues are in the +2 position (Supplementary Fig. 17).

To demonstrate potential applications in SNV detection, we designed a base gap switch specific to an SNV in cfDNA associated with *EGFR*. A T790M mutation (C–T, A**C**G to A**T**G) in *EGFR* has been strongly linked to resistance to tyrosine kinase inhibitors^53–55^, an important class of therapeutics for metastatic non-small cell lung cancers. Ma et al. recently reported a cfDNA specific to *EGFR*, with a T790M mutation conferred through a C–T mutation (T–M)^56^. We designed a base gap switch complementary to this sequence and installed it on a gene encoding for Nluc. Titration of synthetic analogues of both mutant (T790M) and wild-type (WT) triggers against this SGC confirmed the specificity of the switch (Fig. 5d(i) and Supplementary Fig. 18). To further demonstrate the potential of this method, we incubated the SGC with varying fractions (0–10%) of mutant trigger spiked into the WT trigger and observed significance (*P* < 0.01) at variant allele fractions at or above 0.5% (Fig. 5d(ii) and Supplementary Fig. 19). Although the links between variant allele fraction and clinical outcomes are not fully understood, fractions greater than 5% have been associated with negative disease progression^57^. This suggests that base gap switch-based assays may hold clinical utility for detecting *EGFR* mutations.

## Discussion

By selectively modulating transcription, base gap switches can exert control over the expression of functionally distinct proteins in response to both ssDNA and dsDNA. This feature, along with their high specificity and orthogonality, makes them powerful components within gene circuits, particularly for biosensing applications.

A key feature of base gap switches is their sensitivity, resulting from their low leakage. Within our small library of base gap switches, we observed ON:OFF ratios as high as 973:1. This feature enables detection of ssDNA targets at concentrations as low as 10 fM when coupled to an Nluc reporter protein (Fig. 5d). Such amplification-free detection limits are comparable to INSPECTR (approximately 1 fM)^29^ and superior to TASER (approximately 5 pM)^31^ and toehold switches (approximately 30–300 nM)^5,6,26,58,59^. We attribute this to differences in their design when compared with traditional riboswitches. Toehold switch-based sensors typically utilize TBS lengths between 12 and 24 nucleotides, leading to relatively low Gibbs free energy values for trigger binding. Longer TBS lengths are typically avoided owing to concerns over increased off-target binding^60,61^, or binding to internal sequences due to secondary structures^4,61^, causing spontaneous actuation. Conversely, base gap switches contain TBS lengths between 25 and 100 nucleotides, granting comparatively higher trigger binding affinities (although potentially slower hybridization kinetics^62^). Unlike toehold switches, off-target binding or internal secondary structures have a lower chance of spontaneously actuating base gap switches, since ligation between the trigger and the switch is essential for maximizing expression. These features enable base gap switches to more effectively detect lower target titres.

Although leakage is low in base gap switches, it is not zero. It is interesting to consider the mechanistic reasons for this. Actuation in base gap switches relies on the inability of RNAP to traverse gaps in dsDNA and the capacity for complementary hybridization to fill these gaps. This leaves few mechanisms for spontaneous actuation. The most likely explanation is that RNAP can traverse the gap through template switching^37^. Indeed, it has been reported that RNAP can bypass template strand gaps as large as 24 bases, although transcription efficiency is reduced^63,64^. In our structure–function study, we found that coupling longer spaces between the T7 promoter and shorter base gaps led to increased leakage (Fig. 2a(iii)). This suggests that RNAP may be better able to traverse smaller gaps when given more opportunity to form stable transcription complexes. Elucidating the source of the spontaneous actuation in this system remains an ongoing area of study.

Despite their low leakage, base gap switches alone cannot detect ultralow target titres (less than femtomolar concentrations). This is a common drawback of transcriptional/translation switches and is typically compensated for by coupling to nucleic acid amplification. Here, we used RPA to achieve single-digit gene copy detection of the TV actin gene. Whilst impressive, the additional complexity, costs and runtimes associated with nucleic acid amplification are non-ideal. We are currently exploring ways to modify base gap switches to enable amplification-free assays. Arguably, the best way to approach this is to further reduce background signals. Multilevel switches, which require several simultaneous inputs for actuation, are particularly promising in this regard^27^.

An important feature of transcriptional/translational switches is their orthogonality. In our orthogonality test of seven base gap switches, we observed an average crosstalk of 0.89% and a maximum crosstalk of 31.26% (Fig. 4a and Supplementary Fig. 12). These values drop to 0.16% and 3.1%, respectively, when we eliminate the combination of switch 1 and trigger 3, which was specifically designed to have a high degree of paired identity (90%). In their original library of toehold switches (26 switches), Green et al. reported a maximum crosstalk of 12%^4^. More recently, McSweeney et al. tested the orthogonality of 17 triggers against a single toehold switch, reporting a maximum crosstalk of 85%, albeit with a high degree of paired identity (87%)^60^. Importantly, we demonstrated that triggers with a high degree of paired identity at the termini (63%) did not cause notable activation of base gap switches (Supplementary Fig. 10). This suggests that primer dimers will not lead to increased assay background, provided they are designed well and the amplicons are of sufficient length to maintain an overall paired identity.

The ability to detect SNVs massively expands the potential applications of base gap switch-controlled SGCs. SNVs are major drivers of antimicrobial resistance, contribute to drug resistance in cancers and are implicated in multiple genetic disorders. Interestingly, base gap switches showed the greatest SNV specificity for cytosine and thymine mutations in the triggers. This could be explained by comparatively lower ligation efficiencies between the cytosine at the 5′ termini of the promoter region and guanine and adenosine nucleotides at the 3′ termini of the trigger. However, a previous study reported that ligation efficiencies are indifferent to the nucleoside at the 3′ termini^51^. An alternative explanation is that transcription rates are slower when cytosine and thymine residues are in the +2 position^38^. Thus, even if ligation were more efficient, the relative expression rates could still be slower than genes containing a mismatch. An experiment using control genes containing the same sequences but without the nicks supports this theory (Supplementary Fig. 17). One notable limitation of our system is the requirement for the SNV to be placed adjacent to the ligation junction. This precludes the use of many nucleic acid amplification techniques, since the SNV would be at the 5′ termini of the primer. Finding ways to couple base gap switch-mediated SNV detection with nucleic acid amplification would greatly improve sensitivity; this is something we are actively investigating.

On the basis of our results, we can propose a basic design strategy for base gap switches. First, TBSs should have high RBS scores (<1,000 should be discarded) and low Gibbs free energy of mRNA hairpin formation. Second, for general targets, a short (five nucleotide) spacer should be included between the promoter and the TBS. Third, for SNV detection, no spacer should be employed, with the mismatch placed directly after the nick in the +2 position.

In summary, base gap switches are simple, adaptable and highly orthogonal tools for regulating in vitro protein expression in response to specific DNA triggers. They can be readily installed onto genes encoding for various proteins and integrated into biosensing assays for multiple diseases. We anticipate that the simple design principles of these switches, coupled with their high signal-to-noise ratio and orthogonality, will catalyse a broader adoption of protein expression-based biosensors.

## Methods

### General

#### Instrumentation

Synthetic DNA concentrations were determined using a NanoDrop Lite (Thermo Fisher Scientific) or a BioPhotometer Plus (Eppendorf). PCR was performed on a Q-Cycler 96 (Hain Lifescience) or a Proflex PCR system (Thermo Fisher Scientific). Fluorescence and luminescence measurements were performed on a Synergy H1 plate reader (Biotek), with data collected using the BioTek Gen5 software (v 3.12.08).

#### Statistical analysis

All replicates were measured using distinct samples. All error bars are reported as the mean ± 1 standard deviation. LoDs are reported as the mean of the negative plus two standard deviations, with LoD lines on plots corresponding to the two standard deviations. For statistical analyses, unpaired, two-sided Mann–Whitney tests were performed on six independent replicates. All data were analysed and plots generated using GraphPad Prism V10.

#### Slope-based luminescence analysis

For Nluc readouts, we employed a slope-based analysis. The first derivative of the raw luminescence curves was calculated, and the point of the maximum slope was used as the readout for expression.

### Gene circuit synthesis

#### PCR

Specific forward and reverse primers (20 µl, 20 µM in Tris–HCl, 10 µM, pH 7.6) (Supplementary Table 1) (Microsynth) were added to 200 µl of a solution of Q5 High-Fidelity 2× Master Mix (New England Biolabs) and chilled on ice. To this, 144 µl of UltraPure H_2_O (Thermo Fisher Scientific) was added, followed by the plasmid or linear gene (16 µl, 0.2 ng µl^−1^ in Tris–HCl, 5 mM, pH 8.5) (Supplementary Table 2) (Twist Biosciences). Thermal cycling was performed according to the manufacturer’s instructions. Samples were combined and purified using a NucleoSpin Gel and PCR Clean-up Kit (Macherey-Nagel).

#### Ligation

A generalized synthesis protocol for installing base gap switches onto genes is now described. Details of specific encoders and template/coding strand sequences are provided in Supplementary Tables 3 and 4. Unless specifically mentioned, all oligonucleotides were purchased from Microsynth.

To QuickLigase buffer (13 µl, 2×) (New England Biolabs), a solution of the encoder (8 µl, 0.25 µM in Tris–HCl, 5 mM, pH 7.6), the template or coding strand (3.2 µl, 100 µM in Ultrapure H_2_O, 160 equiv.) and a complementary coding or template strand (2.0 µl, 400 µM in Ultrapure H_2_O, 400 equiv.) were added. The reaction mixture was incubated at 4 °C for 30 min, after which, QuickLigase (0.5 µl, cooled to 4 °C) (New England Biolabs) was added and the reaction incubated at 4 °C overnight. The reaction was subsequently diluted to a total volume of 100 µl with Ultrapure H_2_O and purified using a DNA NucleoSpin Gel and PCR Clean-up Kit (Macherey-Nagel). The purification step was repeated three times. To ensure complete purity, agarose gel extraction was used to purify the *Nluc* genes used in the structure–function study (Fig. 2). In brief, the unstained bands of a 1% agarose gel were cut out and then purified once using a DNA NucleoSpin Gel and PCR Clean-up Kit (Macherey-Nagel).

#### Agarose gel electrophoresis

Agarose gels (1%) were prepared by dissolving UltraPure Agarose (Thermo Fisher Scientific) in Tris–acetate–EDTA buffer (1×) (Thermo Fisher Scientific). Samples (5 µl, 8 ng µl^−1^) were mixed with Purple Loading Dye (1.5 µl, 6×) (New England Biolabs) and heated at 65 °C for 5 min before being loaded onto the gel, which was run under constant voltage (80 V) for 100 min in Tris–acetate–EDTA buffer (1×). Gels were subsequently stained for 30 min using GelRed (50 ml, 3×) (Biotium) and imaged using a GelDoc (Bio-Rad).

### In vitro transcription/translation

Below are generalized and optimized protocols for protein expression from base gap-controlled SGCs. Concentrations of circuits, targets and ligases were adjusted during optimization, as described in each figure. Details of specific circuit and trigger sequences are provided in Supplementary Tables 4, 5 and 6. For fluorescent proteins, the following filter settings were employed: Ex_485 ± 20 nm_/Em_528 ± 20 nm_ (mNeonGreen), Ex_510 ± 14 nm_/Em_540 ± 14 nm_ (mPapaya), Ex_600 ± 20 nm_/Em_660 ± 20 nm_ (mNeptune) and Ex_358 ± 20 nm_/Em_423 ± 20 nm_ (Sirius). For experiments employing Nluc, emission was monitored at 460 ± 40 nm. In each experiment, a negative control (no target) and blank (no gene, no target) measurement were performed. The blank was subtracted from the positive and negative controls. Unless otherwise stated, all experiments were performed in triplicate.

#### Expression from base gap switch-controlled SGCs

##### Expression of fluorescent proteins

To a solution of the gene circuit (10 µl, 38.5 nM in Tris–HCl, 5 mM, pH 7.6), 1 µl of QuickLigase was added. Separately, the template promoter (5.0 µl, 700 nM in 2× QuickLigase buffer) and target (5.0 µl, 700 nM in Ultrapure H_2_O) were mixed. The gene circuit and template promoter/target solutions were then mixed in a 1:1 ratio. To a 1-µl aliquot, PURExpress Mastermix (2.5 µl, 7:3:1 solution A:solution B:Ultrapure H_2_O) was added. The solutions were covered with mineral oil (5 µl, Sigma-Aldrich) and incubated at 37 °C for between 20 and 240 min. Subsequently, samples were diluted into Tris–HCl (45 µl) and the fluorescence measured.

##### Orthogonal expression of multiple fluorescent proteins

To a mixture of gene circuits encoding for mNeptune, mPapaya and mSirius (40 µl, 44 nM of each circuit, in Tris–HCl, 5 mM, pH 7.6), 4 µl QuickLigase was added. Separately, mixtures containing different combinations of targets five, six and seven (5.0 µl, 10.8 µM of each target in 2× QuickLigase buffer) were prepared and mixed 1:1 with the template promoter (5.0 µl, 3,600 nM in 2× QuickLigase buffer). The gene circuit and template promoter/target solutions were then mixed in a 9:1 ratio. To a 1-µl aliquot, PURExpress Mastermix (2.5 µl, 7:3:1 solution A:solution B:Ultrapure H_2_O) was added. The solutions were incubated at 37 °C for 180 min. After this time, samples were diluted into Tris–HCl (45 µl) and fluorescence measured.

##### Expression of NanoLuciferase

Experiments were performed as described above (see ‘Expression of fluorescent proteins’ section), with the addition of 1% Nano-Glo Luciferase Assay Reagent (Promega) included in each sample. Experiments were performed on a 1,536-well plate at 37 °C, with a total incubation time of 180 min and luminescence recorded every 3 min.

##### Basepair mismatch discrimination

Experiments were performed as described above (see ‘Expression of fluorescent proteins’ and ‘Expression of NanoLuciferase’ sections), with QuickLigase replaced by HiFi Taq ligase (New England Biolabs) and QuickLigase buffer 2× replaced with HiFi Taq buffer 2× (New England Biolabs). Volumes remained the same.

#### Computational parameter prediction

RBS scores were calculated using the Salis lab RBS calculator version 2020^39,40^, using *Escherichia coli* NZ_CP053601 as a proxy for PURExpress. Each input mRNA started at the +1 position (TATA**G**) and included the entire Nluc CDS. The RBS scores for both start positions encoding a functional protein (+85, +88) were summed and used in the subsequent analyses. Gibbs free energies of hairpin formation were calculated using RNAstructure version 6.5^65^. In brief, mRNA sequences starting at the +1 position (TATA**G**) and terminating at the end of the TBS region of the switch were folded into hairpins, and the Gibbs free energies calculated

#### RPA–IVTT assays

##### TV genomic DNA

RPA of the target (*Trichomonas vaginalis* G3 strain genomic DNA; ATCC PRA-98) was performed using the TwistAmp Basic kit (TwistDX) according to the manufacturer’s instructions. In brief, for an *n* = 6 titration curve of 6 samples and 1 negative (63 samples), forward and reverse primers (76.8 µl), (10 µM in Ultrapure H_2_O) were mixed with 944.0 µl of primer dehydration buffer, followed by 262.4 µl of d.d. H_2_O and 32 TwistAmp pellets. This MasterMix was split into 21.25-µl aliquots, and to each aliquot the target (2.5 µl, 10 fM–0.1 aM in d.d. H_2_O) was added, followed by MgOAc (1.25 µl, 280 mM). Non-template controls (NTCs; d.d H_2_O) were prepared similarly. All samples were then incubated at 37 °C for 20 min. The samples were subsequently heat-inactivated at 75 °C for 20 min and mixed 1:1 with template promoter (14 nM, QuickLigase buffer). The IVTT experiments were performed as described above (see ‘Expression of NanoLuciferase’ section); however, PUREfrex 2.0 (GeneFrontier Corporation) was replaced as the reconstituted cell-free protein synthesis reagent. In brief, a PUREfrex MasterMix was prepared (10:1:2 solution 1:solution 2:solution 3), and diluted into an equal volume of d.d. H_2_O. For each replicate, a 3-µl aliquot of this MasterMix was prepared and mixed with 1 µl of the deactivated sample. The solutions were covered with mineral oil (5 µl) and incubated in a 1,536-well plate at 37 °C, with a total incubation time of 180 min and luminescence recorded every 3 min.

##### TV patient samples

The collection and processing of swab samples are described in the AVEONS study^66^. Clinical specimens were obtained from asymptomatic pregnant women in the Democratic Republic of the Congo^66^. Cervicovaginal lavage samples were collected by rinsing the cervical mucosa with 5 ml of sterile physiological water. Sample collection, handling and storage procedures have been described previously^67^. In brief, DNA was extracted from cervicovaginal lavage samples using the RNeasy PowerMicrobiome Kit (Qiagen) according to the manufacturer’s instructions, with minor modifications (omission of the DNase treatment step). Detection and quantification of *T. vaginalis* DNA were performed using the S-DiaMGTV qPCR kit (Diagenode), following the manufacturer’s protocol as previously described^68^. This assay provides quantitative estimates of target DNA concentration (copies per microlitre), which were used to stratify samples into predefined categories (high, medium, low and ultralow target loads). The resulting qPCR data (Supplementary Table 7) served as the reference method for subsequent comparison with the base gap switch-based assay, for which amplification of the target and protein expression were carried out as described above.

##### NG and CT

RPA amplification of the target (C*hlamydia trachomatis* 16S rRNA and *Neisseria gonorrhoeae porA*) was performed as described above (for CT 16S rRNA, the MgOAc concentration was 560 nM) and mixed 1:1 with template promoter (800 nM, rCutSmart buffer, 1×) (New England Biolabs). To a solution of restriction-enzyme encoding genes (15 µl, 88 nM in rCutSmart buffer, 1×), Quick Ligase (4 µl) was added, and the resulting solution mixed 1:1 with the RPA sample/template promoter mixtures. Then, 2.5 µl of the resulting solutions were subsequently mixed with 7.5 µl of PUREfrex 2.0 MasterMix containing 658 nM of each molecular beacon (Supplementary Table 8). The solutions were transferred to a 384-well plate, covered with mineral oil (5 µl) and incubated at 37 °C for 360 min. Fluorescence (Ex_343 ± 10 nm_/Em_441 ± 10 nm_ and Ex_552 ± 10 nm_/Em_578 ± 10 nm_) was measured every 2 min. Values on the *y* axes of the graphs are reported as the gradient of the resulting curves.

### Reporting summary

Further information on research design is available in the Nature Portfolio Reporting Summary linked to this article.

## Supplementary information

Supplementary Information
Supplementary Figs. 1–19.

Reporting Summary

Peer Review File

Supplementary Tables 1–8

## Source data

Source Data Fig. 1
Unprocessed agarose gel.

Source Data Fig. 1
Source data for Fig. 1.

Source Data Fig. 2
Source data for Fig. 2.

Source Data Fig. 3
Source data for Fig. 3.

Source Data Fig. 4
Source data for Fig. 4.

Source Data Fig. 5
Source data for Fig. 5.

## Data Availability

Source data are provided with this paper. These data are also available available via Zenodo at https://doi.org/10.5281/zenodo.21079229 (ref. ^69^).
