# Supplementary material for: Base gap switches as transcriptional regulators and their applications as biosensors
Source: Nat Sens. 2026 Sep 14;1(10):956–66. doi: 10.1038/s44460-026-00134-z (PMC13642041; doi:10.1038/s44460-026-00134-z)

nature sensors

Supplementary information https://doi.org/10.1038/s44460-026-00134-z

Base gap switches as transcriptional regulators and their applications as biosensors

In the format provided by the authors and unedited

DATA **2**
SUPPLEMENTARY FIGURE 1 2
SUPPLEMENTARY FIGURE 2 3
SUPPLEMENTARY FIGURE 3 4
SUPPLEMENTARY FIGURE 4 5
SUPPLEMENTARY FIGURE 5 6
SUPPLEMENTARY FIGURE 6 7
SUPPLEMENTARY FIGURE 7 8
SUPPLEMENTARY FIGURE 8 10
SUPPLEMENTARY FIGURE 9 11
SUPPLEMENTARY FIGURE 10 12
SUPPLEMENTARY FIGURE 11 13
SUPPLEMENTARY FIGURE 12 14
SUPPLEMENTARY FIGURE 13 16
SUPPLEMENTARY FIGURE 14 17
SUPPLEMENTARY FIGURE 15 18
SUPPLEMENTARY FIGURE 16 19
SUPPLEMENTARY FIGURE 17 20
SUPPLEMENTARY FIGURE 18 21
SUPPLEMENTARY FIGURE 19 22
BIBLIOGRAPHY **23**
SOURCE DATA **23**

Data

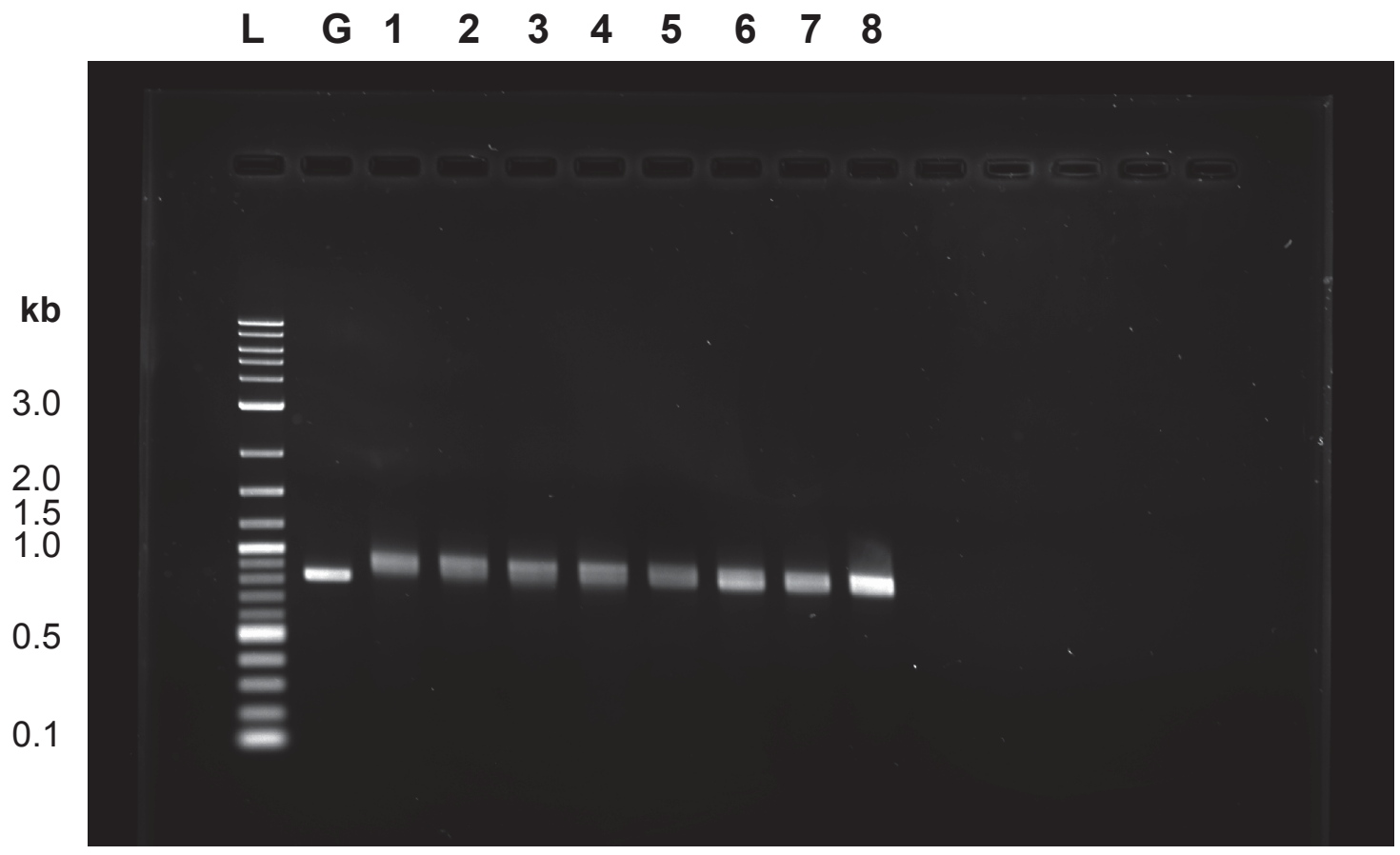

Supplementary Figure 1. Agarose gel characterisation of a Base Gap switch installed onto an mNeonGreen gene. L = DNA ladder, G = starting mNeonGreen gene, 1 – 8 = mNeonGreen gene (G) attached to Base Gap switches, 2, 13, 14, 17, 18, 19, 29, and 42.

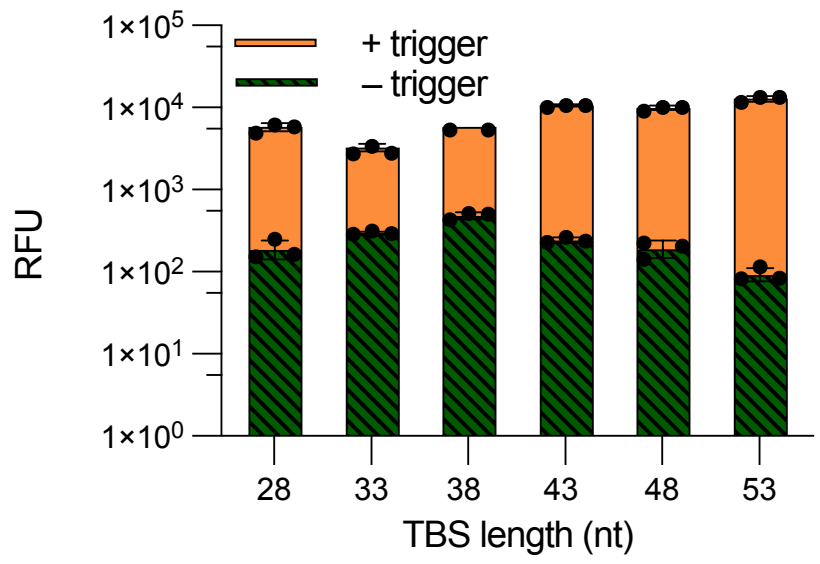

Supplementary Figure 2. Relative mNeonGreen fluorescence from a gene controlled by Base Gap switches with gaps on the coding strand. Error bars represent the mean of three individual measurements (n = 3) ± one standard deviation.

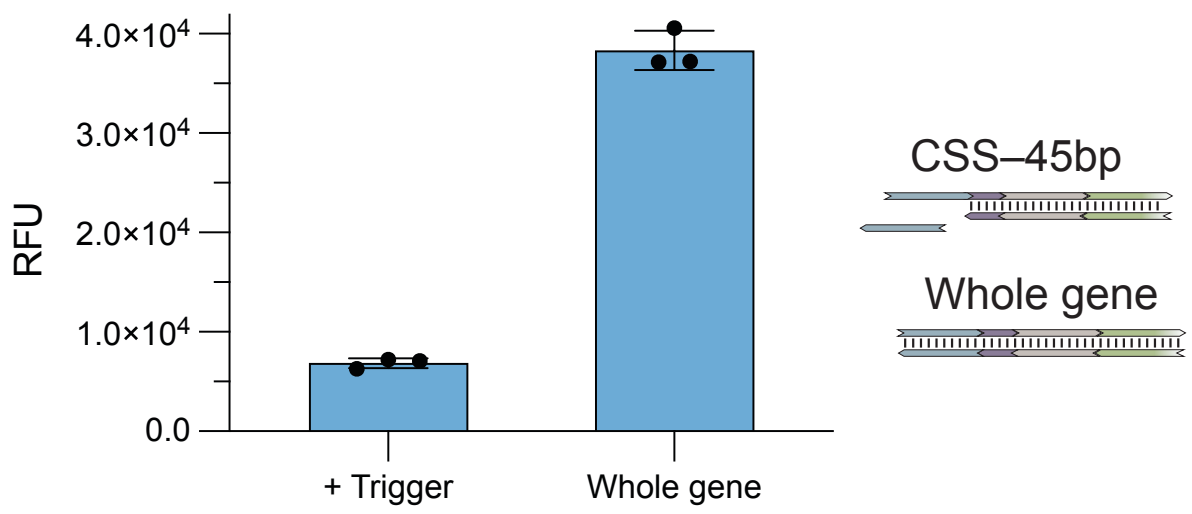

Supplementary Figure 3. Comparison of relative mNeonGreen fluorescence from a gene controlled by a Base Gap switch and an unmodified gene. The nucleotide sequence is identical. Introducing the Base Gap switch decreases expression compared to the control gene. Error bars represent the mean of three individual measurements (n = 3) ± one standard deviation.

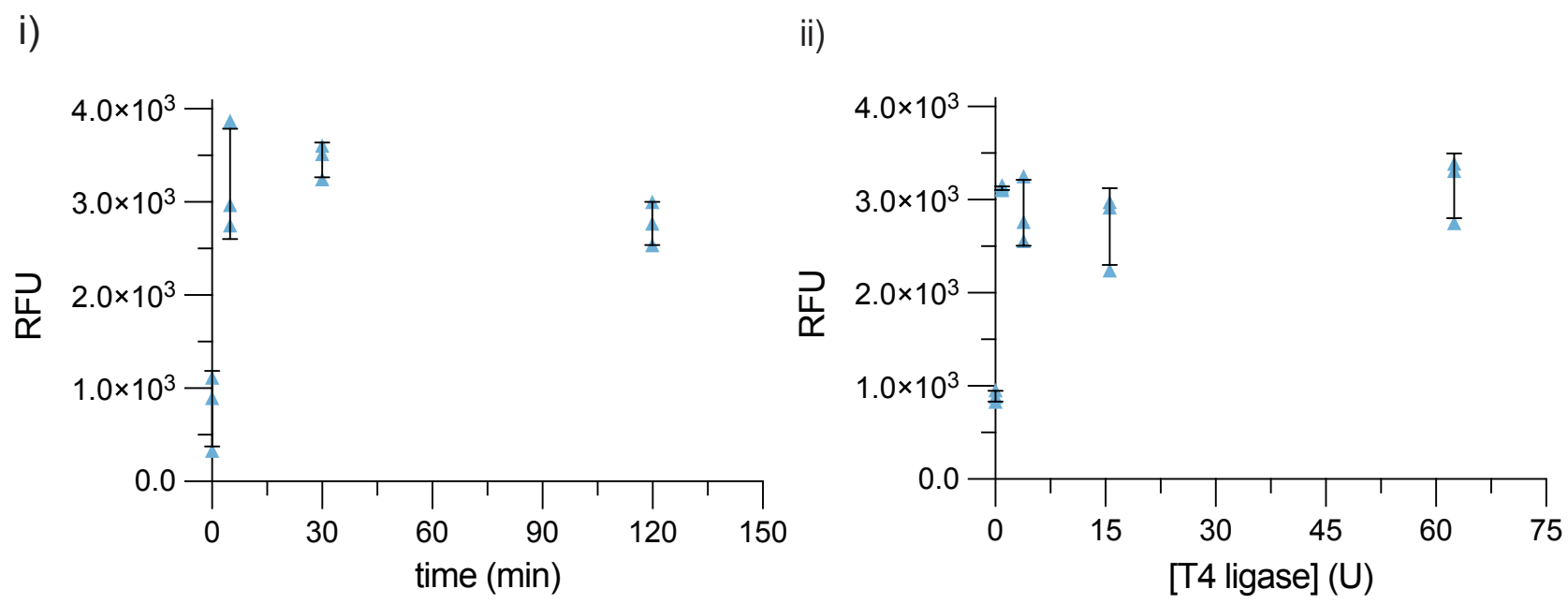

Supplementary Figure 4. mNeonGreen fluorescence as a function of (i) pre-ligation time and (ii) ligase concentration. Extended incubation times or high ligase concentrations do not significantly improve protein expression. Error bars represent the mean of three individual measurements (n = 3) ± one standard deviation.

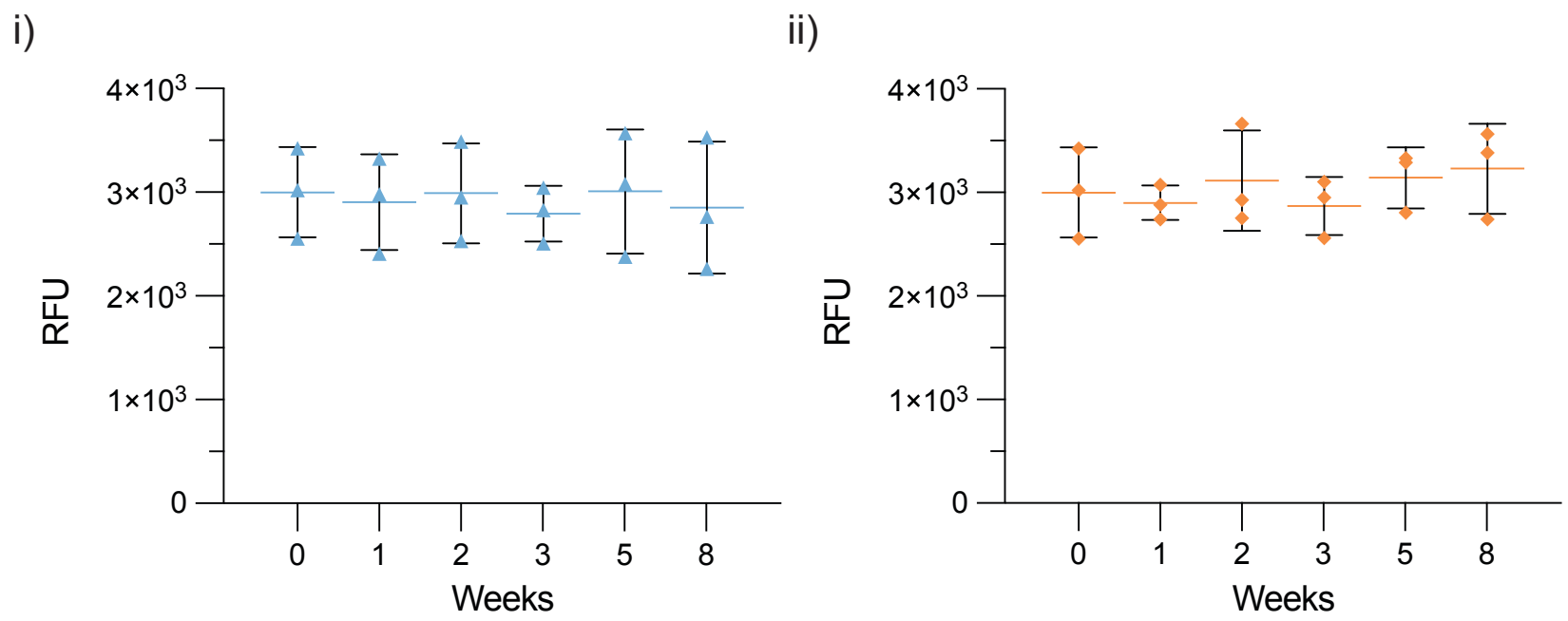

Supplementary Figure 5. Relative mNeonGreen fluorescence from a Base Gap-controlled gene after storage at (i) 4°C and (ii) 21°C. The performance remained stable. Incubation times relate only to the DNA circuits; all other components (enzymes, PURExpress, etc) were stored at -20 °C. Error bars represent the mean of three individual measurements (n = 3) ± one standard deviation.

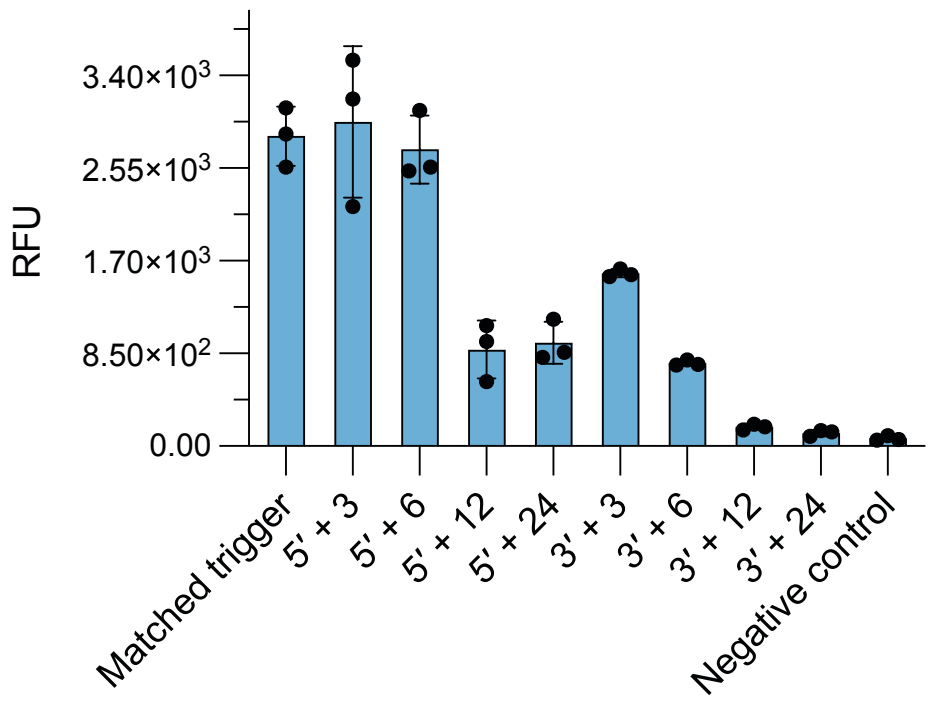

Supplementary Figure 6. Relative mNeonGreen fluorescence as a function of extended trigger length. Extensions at the 5′ end of the trigger are better tolerated, though longer extensions at both termini decrease protein expression. Error bars represent the mean of three individual measurements (n = 3) ± one standard deviation.

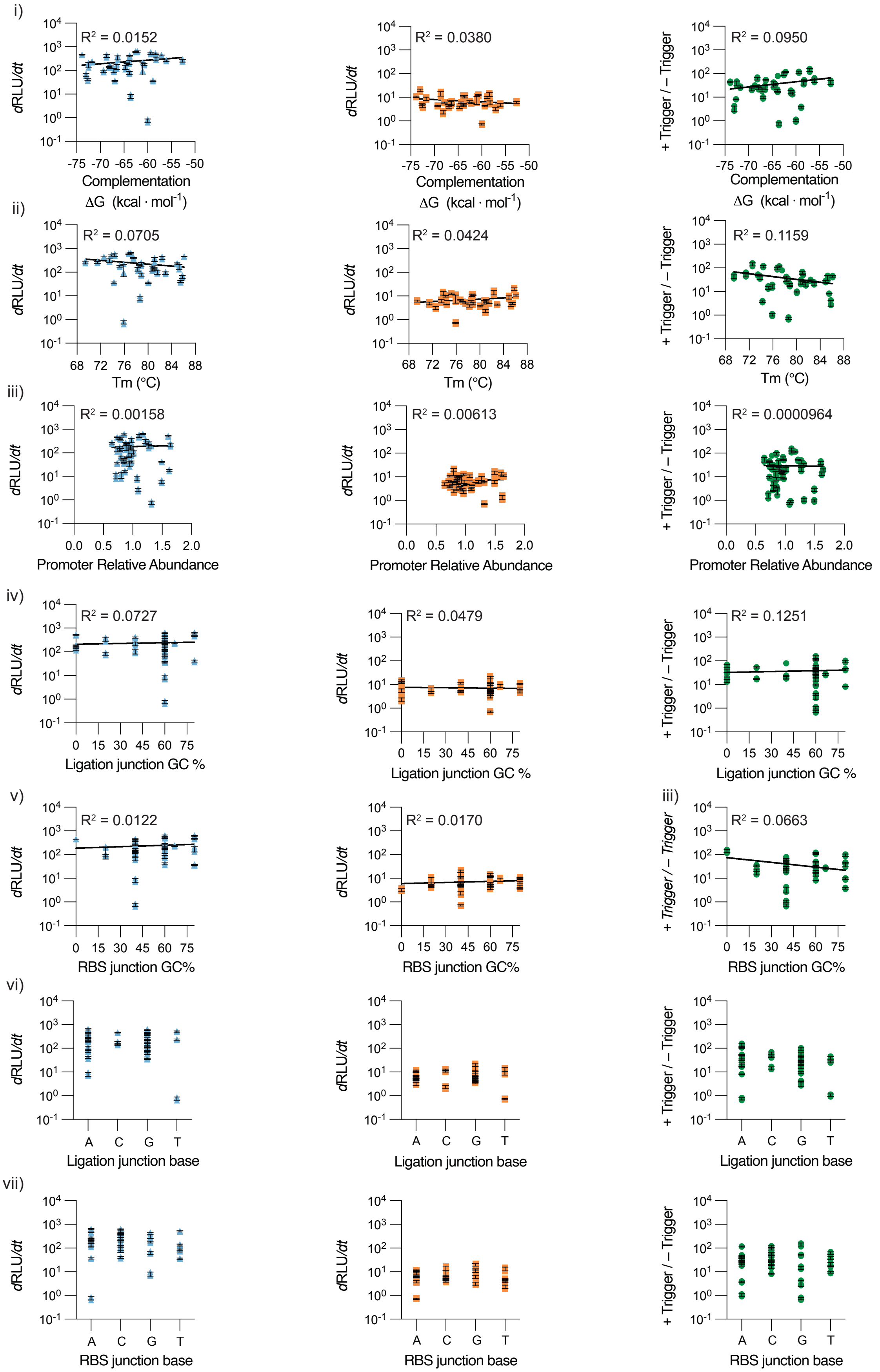

Supplementary Figure 7. NanoLuc Luciferase (Nluc) expression from 45 Base Gap-switch controlled SGCs with varying TBS compositions as a function of (i) the Gibbs free energy of complementation, (ii), the melting

temperature (TM) of complementation, (iii) relative promoter strength, as determined by Conrad *et al.*, [1](iv) GC% of the five bases after the ligation junction, (v) GC% of the five bases before the RBS junction, (vi) the nucleotide at the ligation junction, and (vii) the nucleotide at the RBS junction Data shows expression in the presence (+ Trigger, blue triangles) and absence (– Trigger, orange squares) of triggers, as well as the ratio of the two (+ Trigger : – Trigger, green circles). Error bars represent the mean of three individual measurements (n = 3) ± one standard deviation.

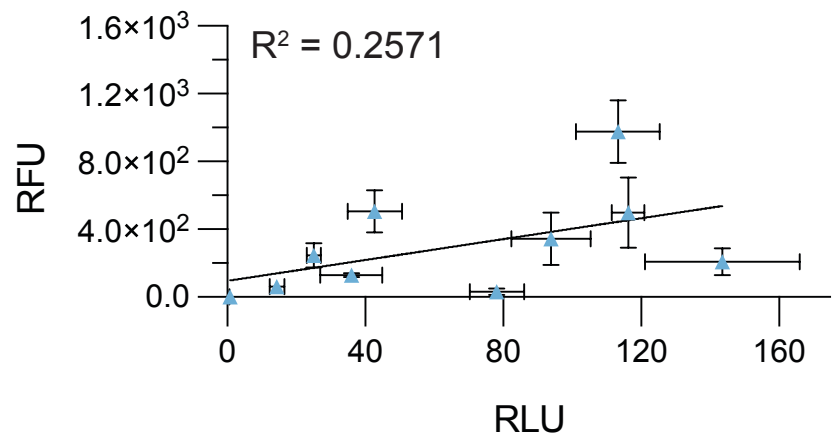

Supplementary Figure 8. Correlation between NanoLuc Luciferase (Nluc) and mNeonGreen expression when the same Base Gap switches are employed. Error bars represent the mean of three individual measurements (n = 3) ± one standard deviation.

A

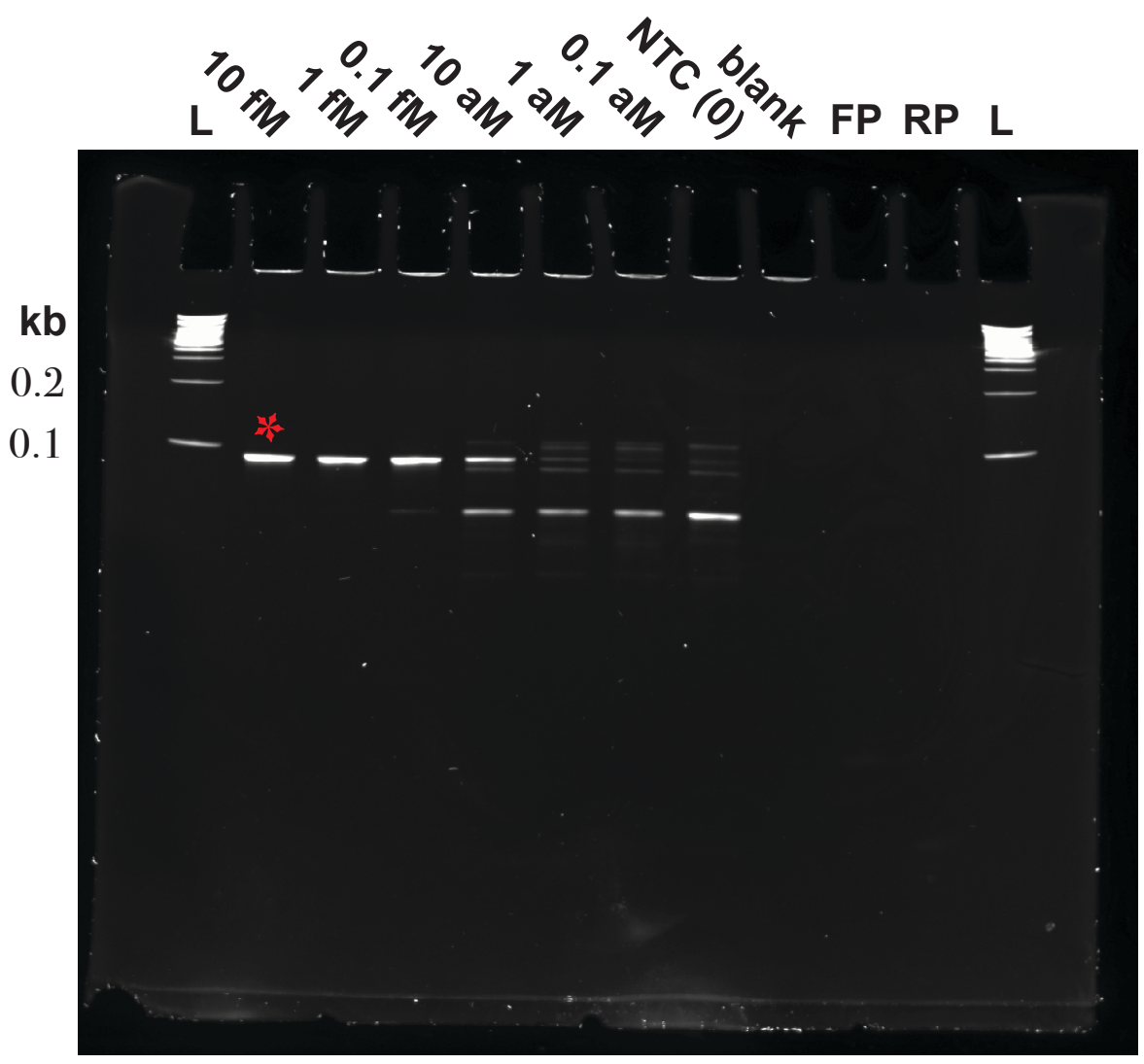

B

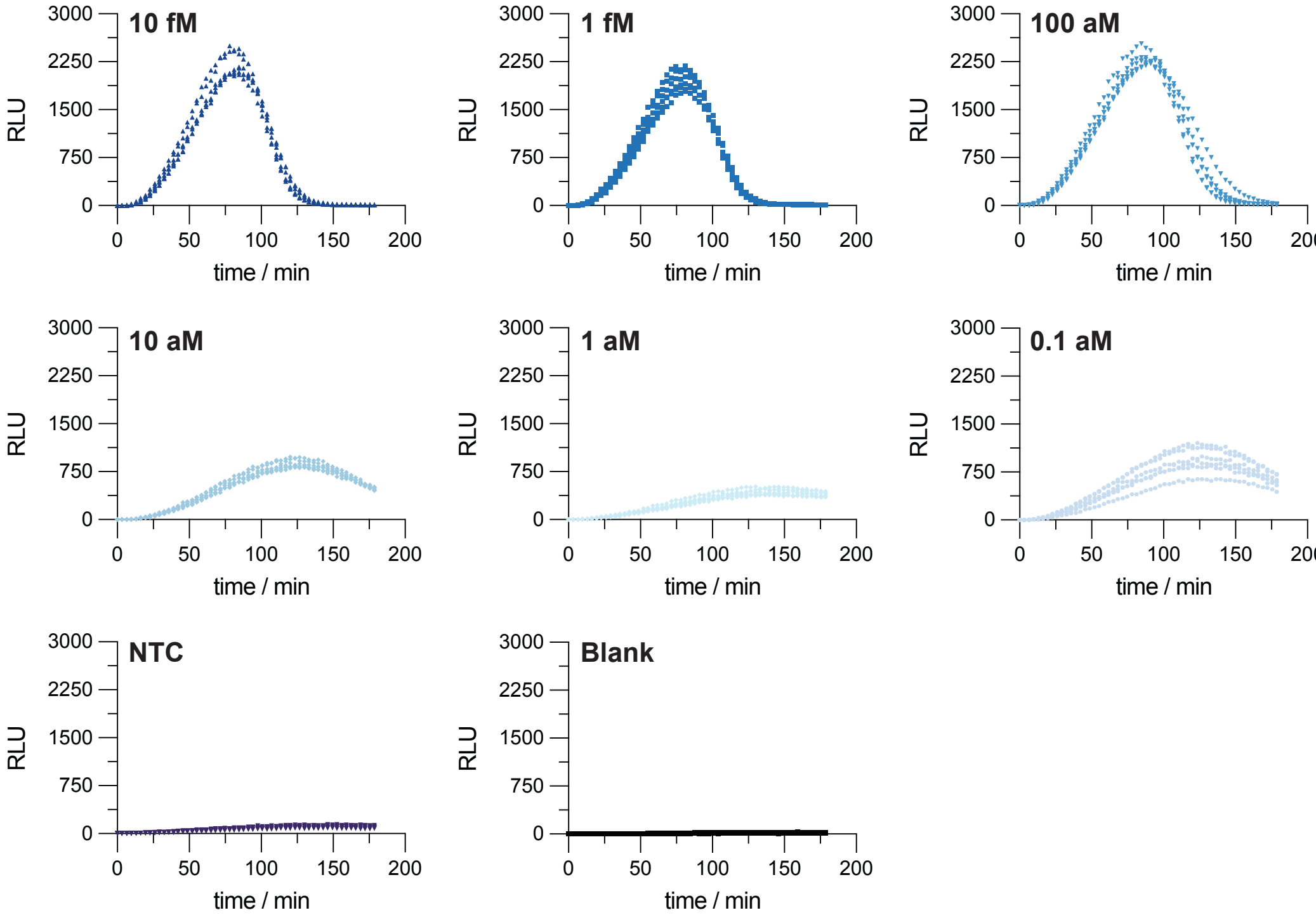

Supplementary Figure 9. RPA–IVTT assay for genomic TV DNA. **(A)** Agarose gel electrophoresis of the products of the RPA. The red asterix (*) marks the expected product. **(B)** Relative NanoLuc Luciferase (Nluc) luminescence as a function of time and genomic DNA concentration. These are the raw curves used to plot the data in **Figure 3B.**

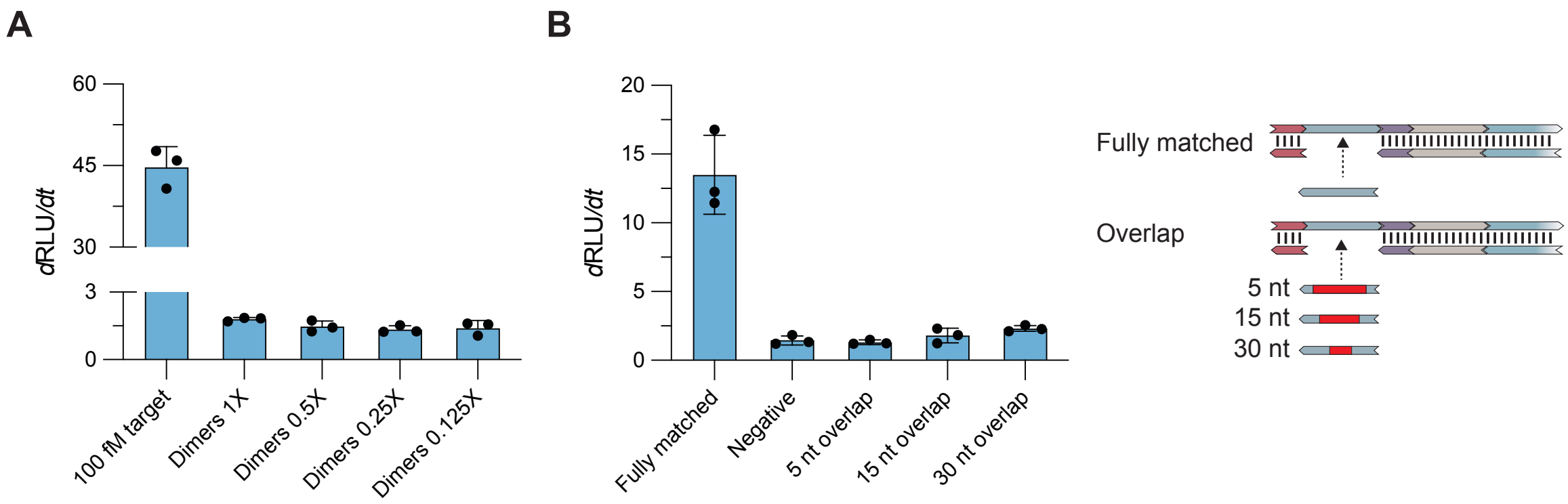

Supplementary Figure 10. NanoLuc Luciferase (Nluc) expression as a function of amplicon dimers. **A)** Dilutions of primer dimers (obtained from RPA in the absence of a target) were incubated with the Nluc circuit with a Base Gap gap switch specific to TV actin. No dose-response was observed. Error bars represent the mean ± one standard deviation. **B)** Relative Nluc luminescence values obtained when only the last 5, 15, or 30 bases at the 5′ and 3′ termini of the trigger match with the TBS. Significant expression over the negative is only observed with a 30 nt overlap. Error bars represent the mean of three individual measurements (n = 3) ± one standard deviation.

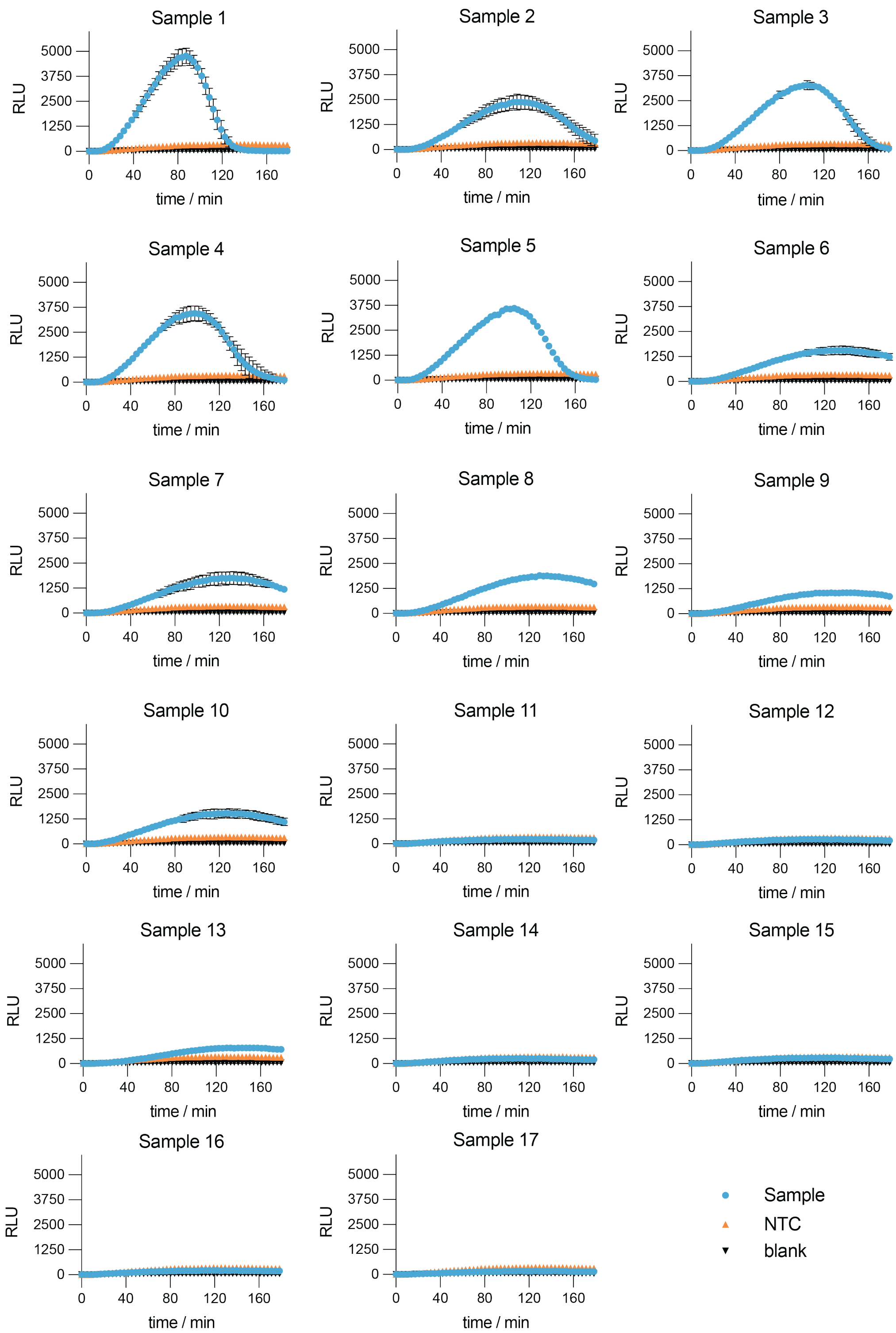

Supplementary Figure 11. Relative NanoLuc Luciferase (Nluc) luminescence *vs* time plots obtained from the TV RPA–IVTT assay for individual patient-derived cervical swabs. Error bars represent the mean ± one standard deviation. Individual points were plotted for clarity.

A

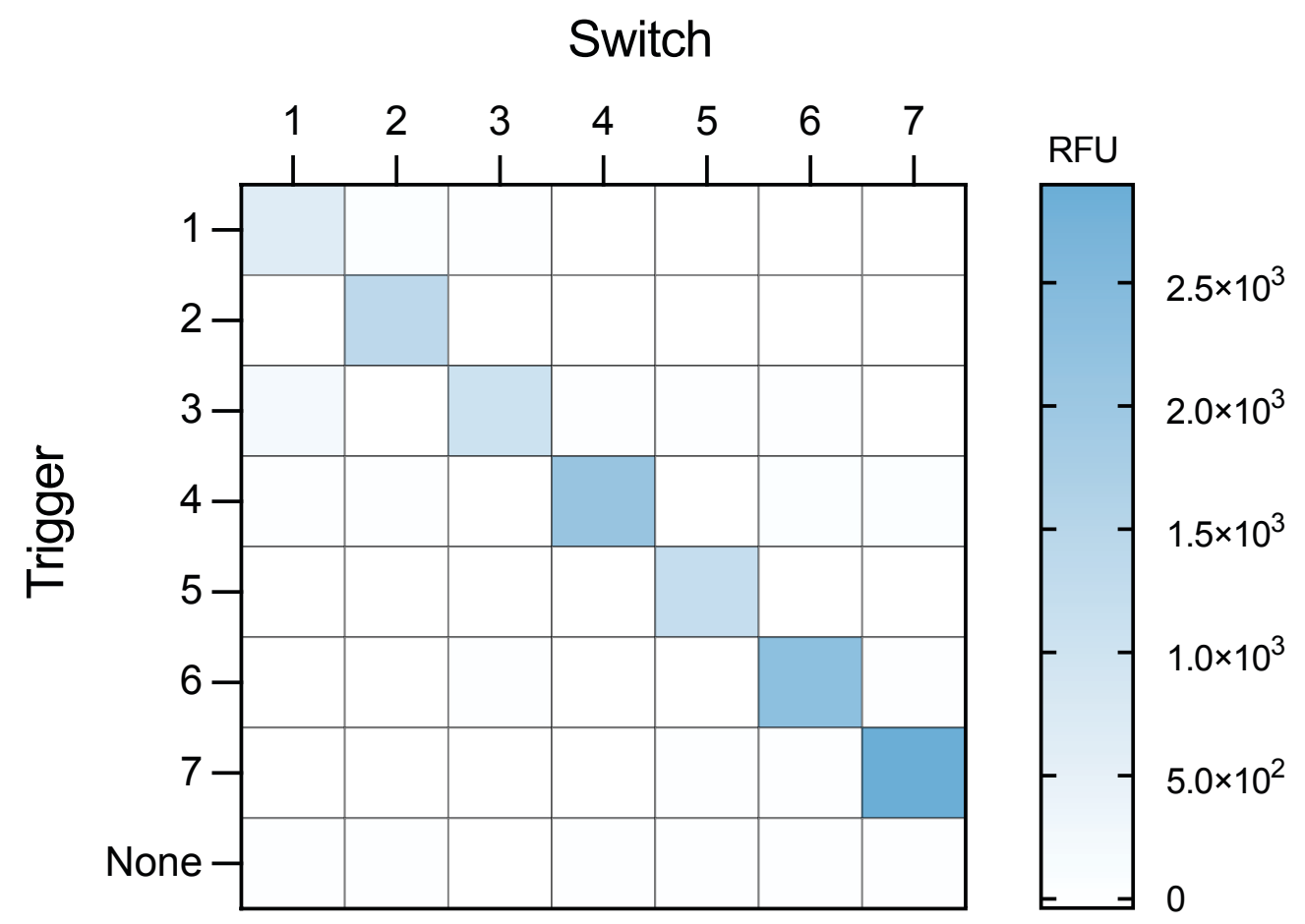

B

Switch (columns) / Trigger (rows)

Trigger	1	2	3	4	5	6	7
1	100.00	3.10	2.38	0.25	-1.71	-0.25	0.30
2	1.10	100.00	-2.16	-0.54	-2.01	-1.21	-0.92
3	31.26	0.40	100.00	0.72	1.01	0.61	0.06
4	3.09	1.64	-0.88	100.00	-3.52	1.37	1.31
5	1.54	0.45	-0.22	-0.35	100.00	0.07	-0.21
6	0.45	0.48	1.50	0.27	-0.62	100.00	0.54
7	-3.33	-0.07	-0.31	0.10	1.06	0.66	100.00

C

Template trigger (columns) / Paired trigger (rows)

Paired trigger	1	2	3	4	5	6	7
1	100.00	26.00	90.00	34.00	34.00	26.00	40.00
2	24.50	100.00	30.20	26.40	32.10	28.30	28.30
3	90.00	32.00	100.00	30.00	38.00	28.00	42.00
4	32.70	26.90	28.90	100.00	25.00	50.00	25.90
5	33.30	29.40	37.30	25.50	100.00	19.60	29.40
6	25.00	28.90	26.90	50.00	19.20	100.00	26.90
7	40.00	30.00	42.00	28.00	30.00	28.00	100.00

D

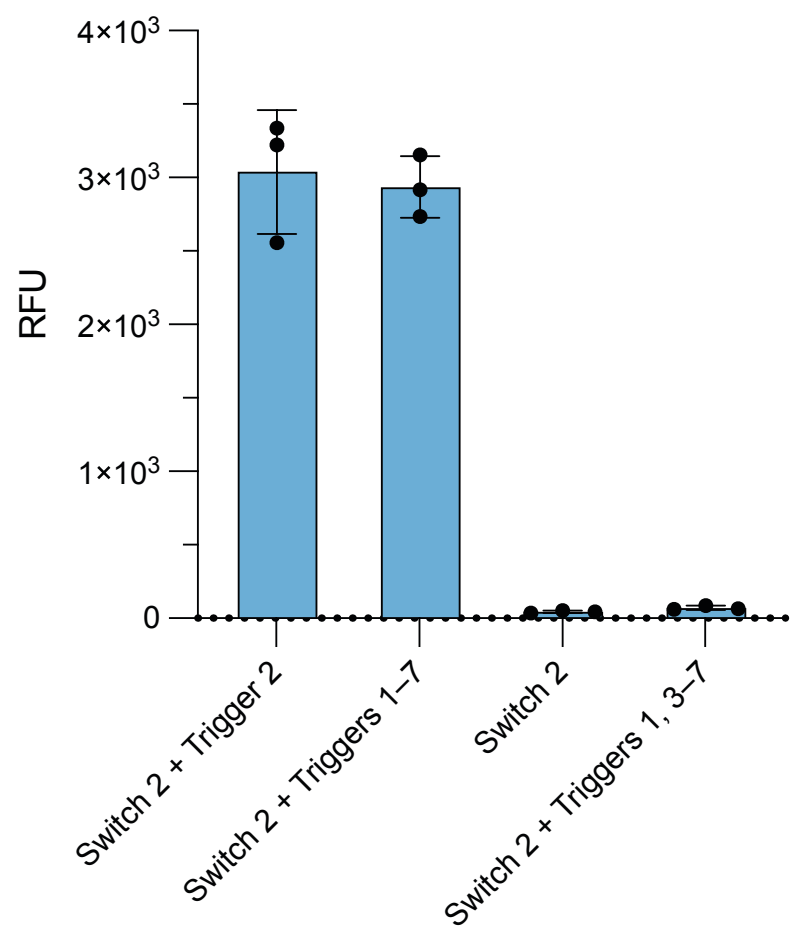

Supplementary Figure 12. Relative mNeonGreen fluorescence from the orthogonal combination of Base Gap switches and triggers. **A)** Relative mNeonGreen fluorescence from 7 pairwise combinations of Base Gap switch-controlled genes and triggers (not-normalised). **B)** Normalised fluorescence values showing the cross-talk from the 7 pairwise combinations of Base Gap switch-controlled genes and triggers.

C) Paired identity between different triggers, determined using Benchling (San Francisco, USA). **D)** Relative mNeonGreen fluorescence from a gene controlled by Switch 2, incubated with a mixture of triggers with and without the cognate trigger 2. Expression rates are not impacted by the presence of additional triggers, and trigger 2 is required for switch activation. Error bars represent the mean of three individual measurements (n = 3) ± one standard deviation.v

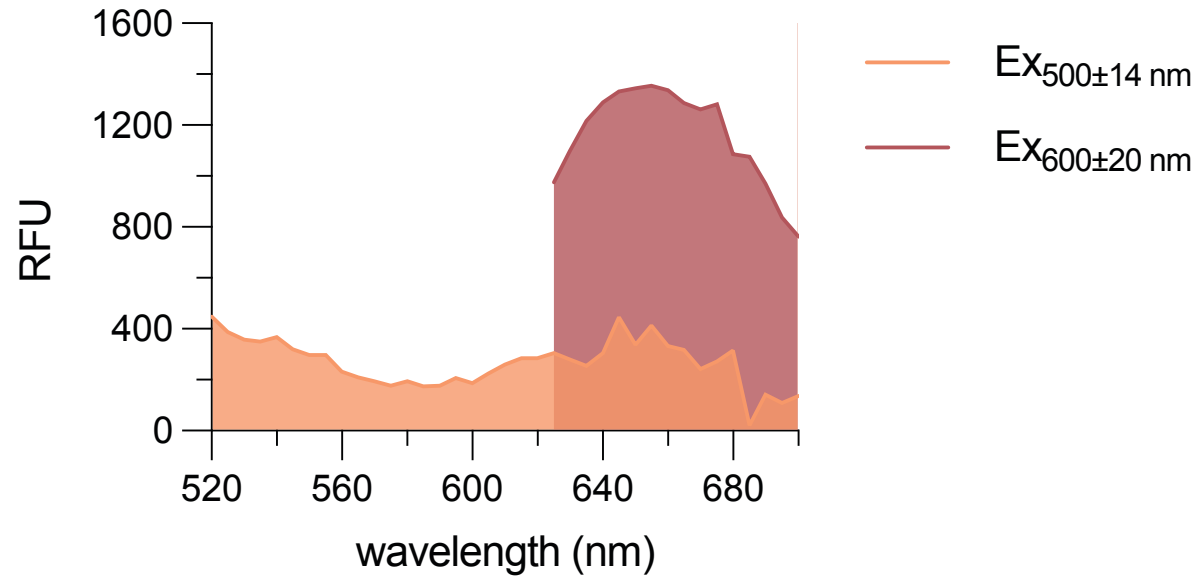

Supplementary Figure 13. Emission spectra for mNeptune obtained at different excitation wavelengths. The data is split into orange (Ex_{500} nm) and red (Ex_{600} nm) channels.

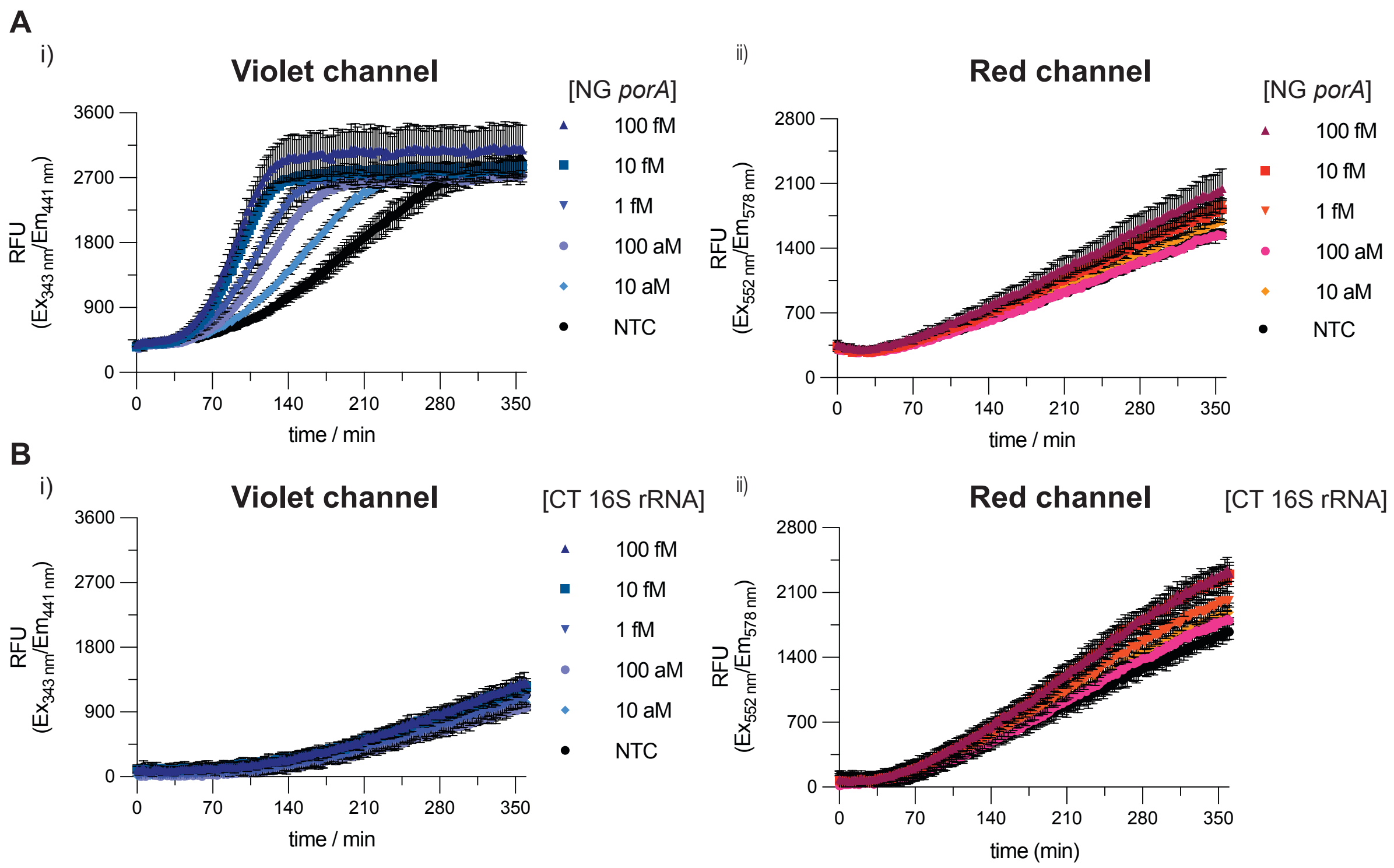

Supplementary Figure 14. Orthogonal expression of restriction enzymes in response to NG and CT DNA RPA amplicons. Fluorescence *vs* time plots as a function of **A)** NG *porA* and **B)** CT 16S rRNA DNA concentration. The data is split into violet (i) and red (ii) channels. Error bars represent the mean of three individual measurements (n = 3) ± one standard deviation. Individual points are not plotted to improve interpretability.

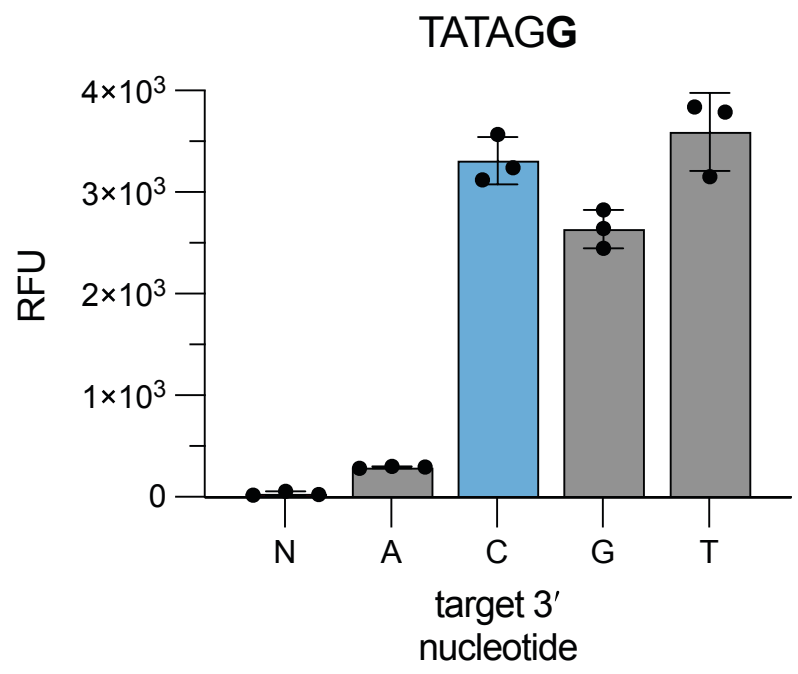

Supplementary Figure 15. Relative mNeonGreen fluorescence values obtained when using T4 ligase to repair nicks between the switch and the trigger, located at the ligation junction. The bolded letters above indicate the nucleotide at the +2 position of the Base Gap switch. The x-axis indicates the nucleotide at the 3′ end of the trigger. *N* = negative (no target). Error bars represent the mean of three individual measurements (n = 3) ± one standard deviation. The complementary base is highlighted in blue.

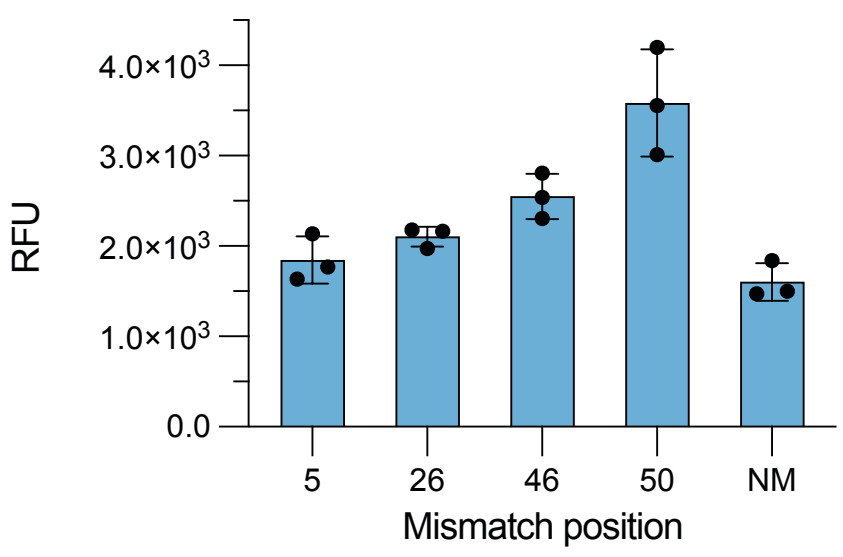

Supplementary Figure 16. Relative mNeonGreen fluorescence values obtained with different base pair mismatches between the switch and the trigger, positioned distal from the ligation junction. NM = no mismatch. Mismatches in the middle of the trigger have no negative impact on expression, and even increase it when placed in certain positions. Error bars represent the mean of three individual measurements (n = 3) ± one standard deviation.

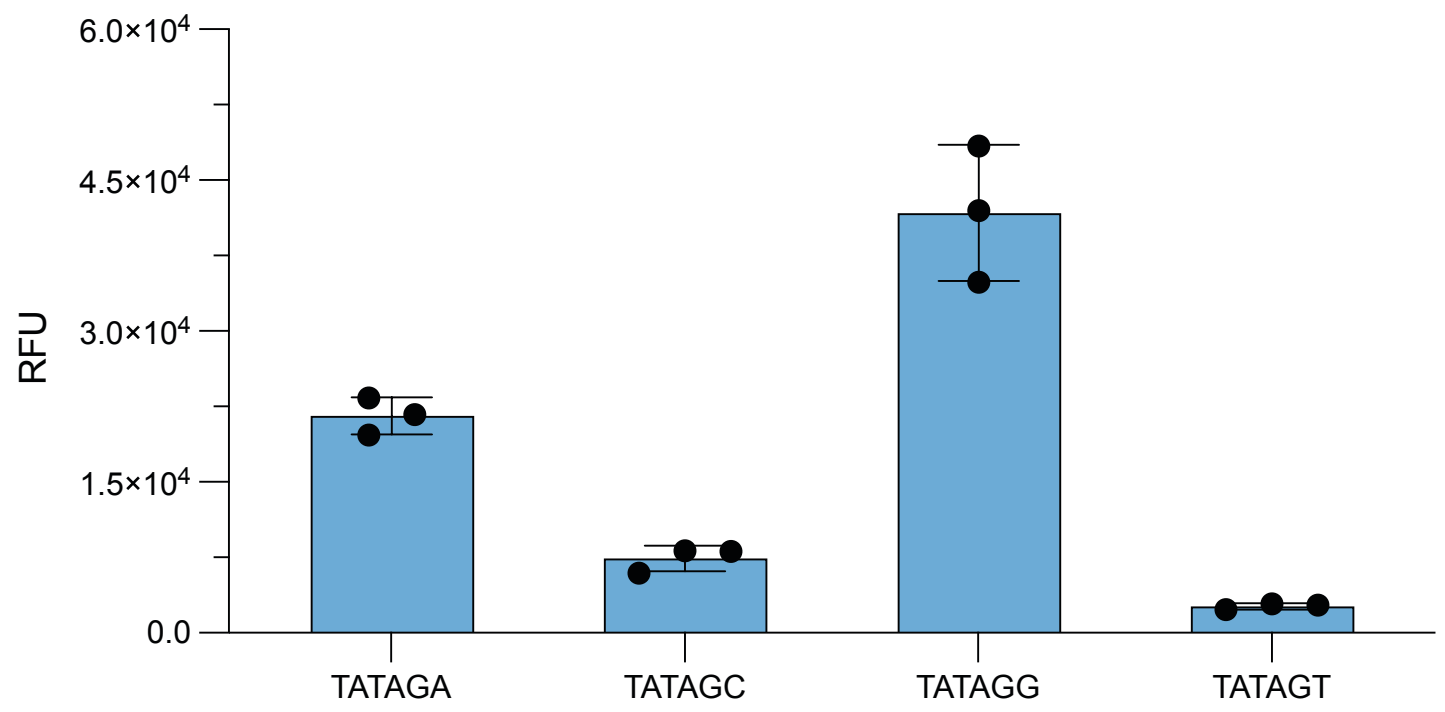

Supplementary Figure 17. Relative mNeonGreen fluorescence as a function of the nucleotide in the +1 position. Expression is greatest with A and G nucleotides in the +2 position. Error bars represent the mean of three individual measurements (n = 3) ± one standard deviation.

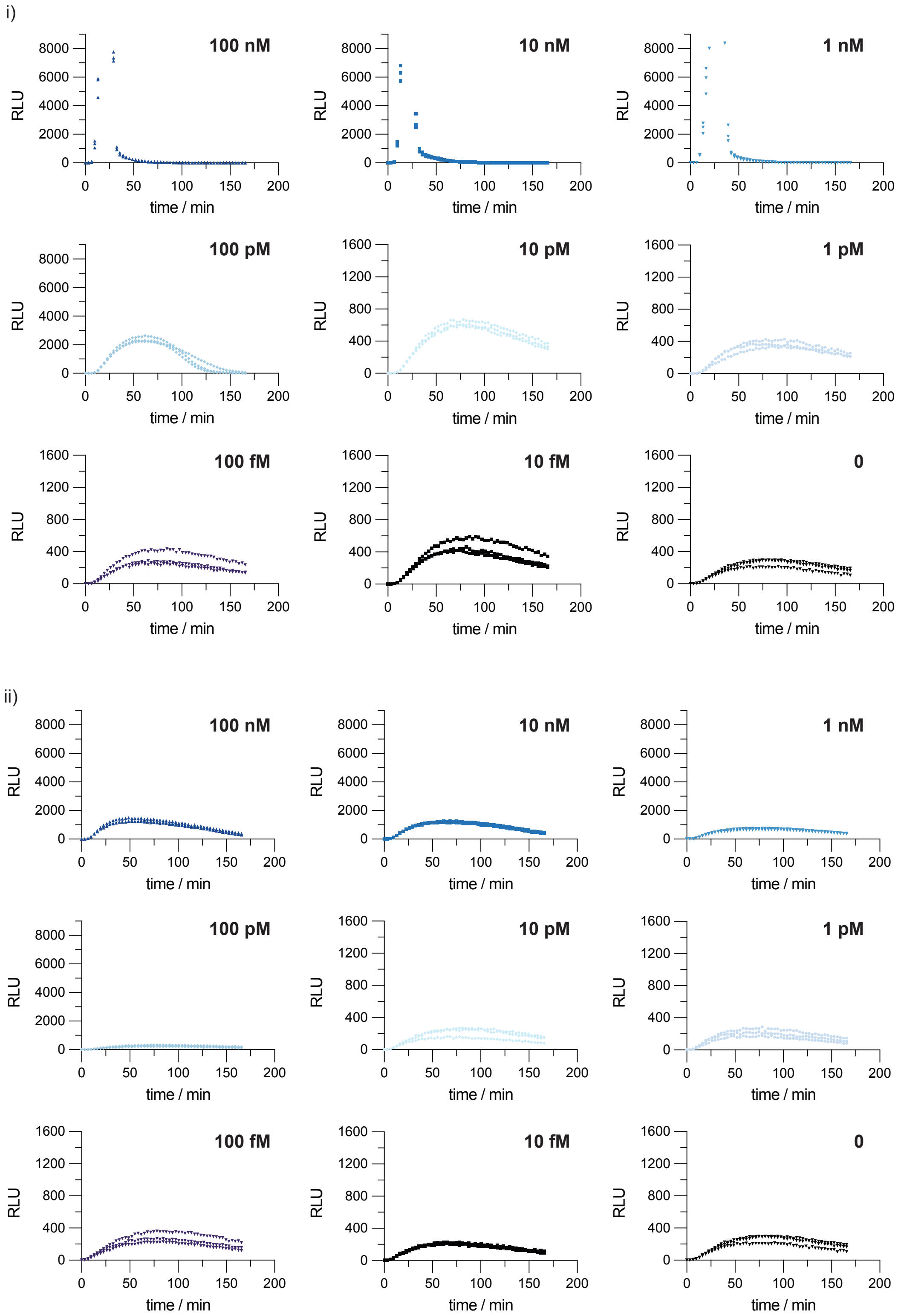

Supplementary Figure 18. Relative NanoLuc Luciferase (Nluc) luminescence as a function of time and (i) EGFR T790M cfDNA concentration or (ii) EGFR wildtype cfDNA. These are the raw curves used to plot the data in **Figure 5Di**. Note the difference in scale from 10 pM to 0.

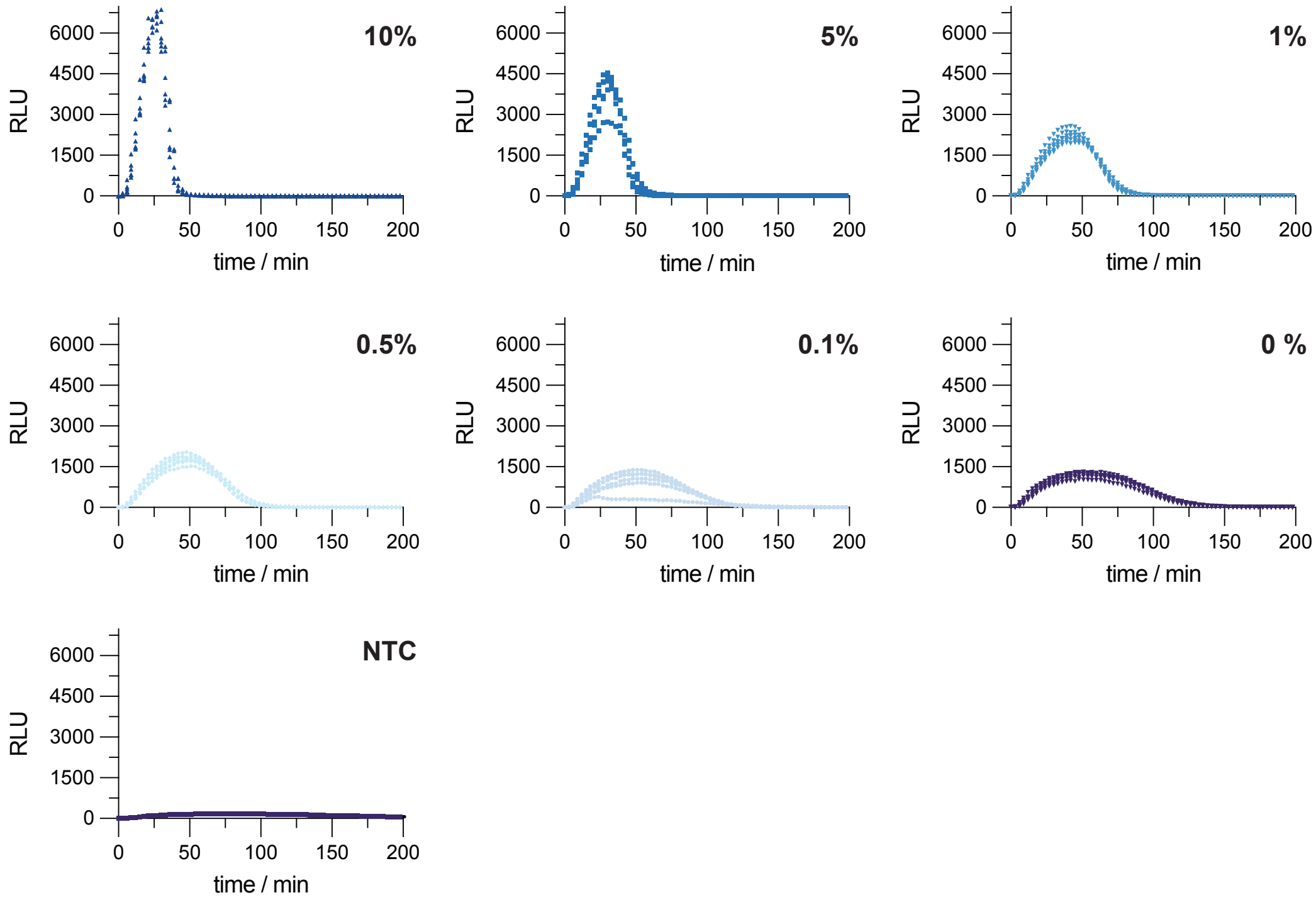

Supplementary Figure 19. Relative NanoLuc Luciferase (Nluc) luminescence as a function of time and EGFR T790M allele fraction. These are the raw curves used to plot the data in **Figure 5D$_{\mathbf{ii}}$**.

Bibliography

[1] T. Conrad, I. Plumbom, M. Alcobendas, R. Vidal, S. Sauer, “Maximizing transcription of nucleic acids with efficient T7 promoters” *Commun. Biol.* **2020**, *3*, 439.

Source data

Agarose gel for Supplementary Figure 1

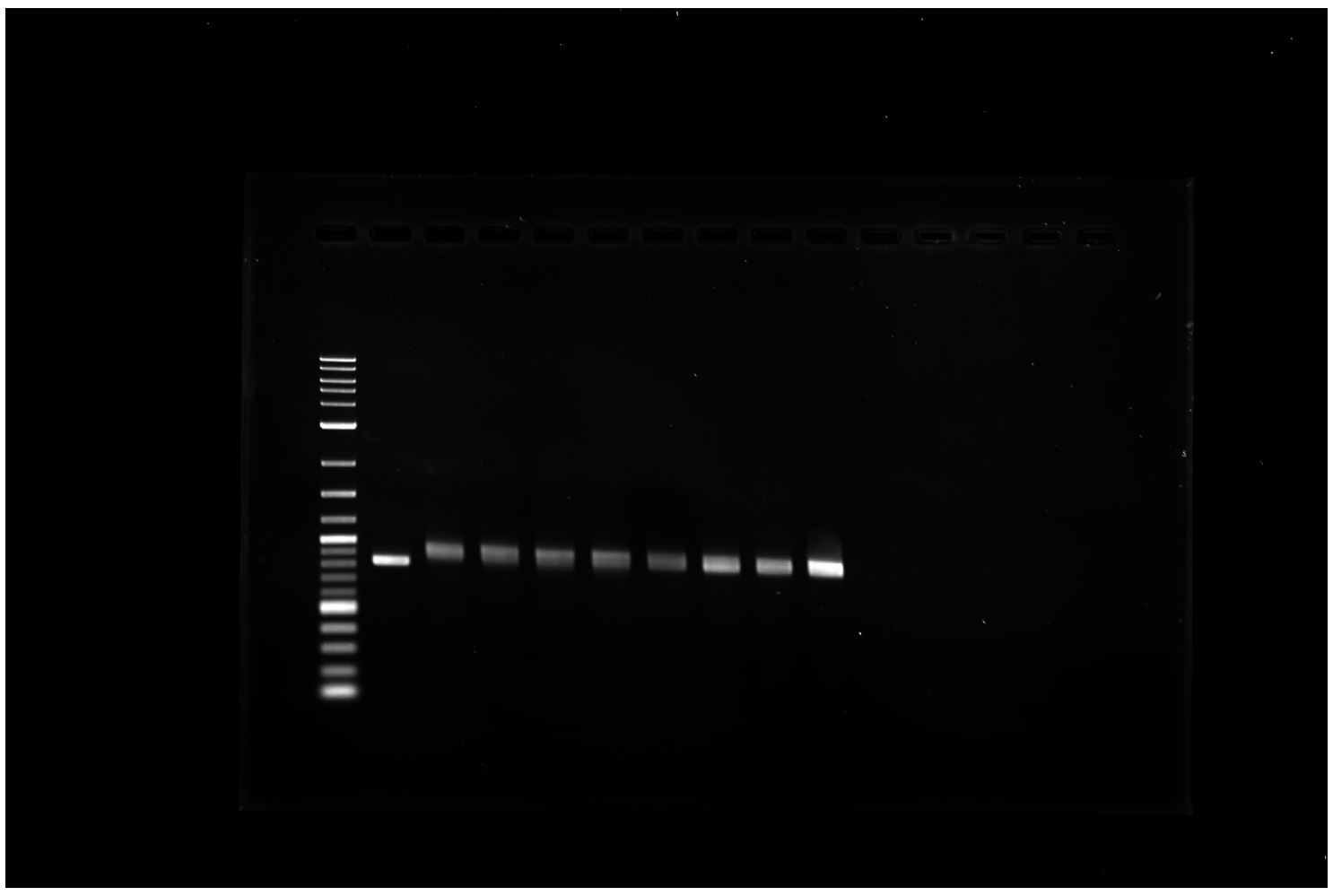

Agarose gel for Supplementary Figure 9A

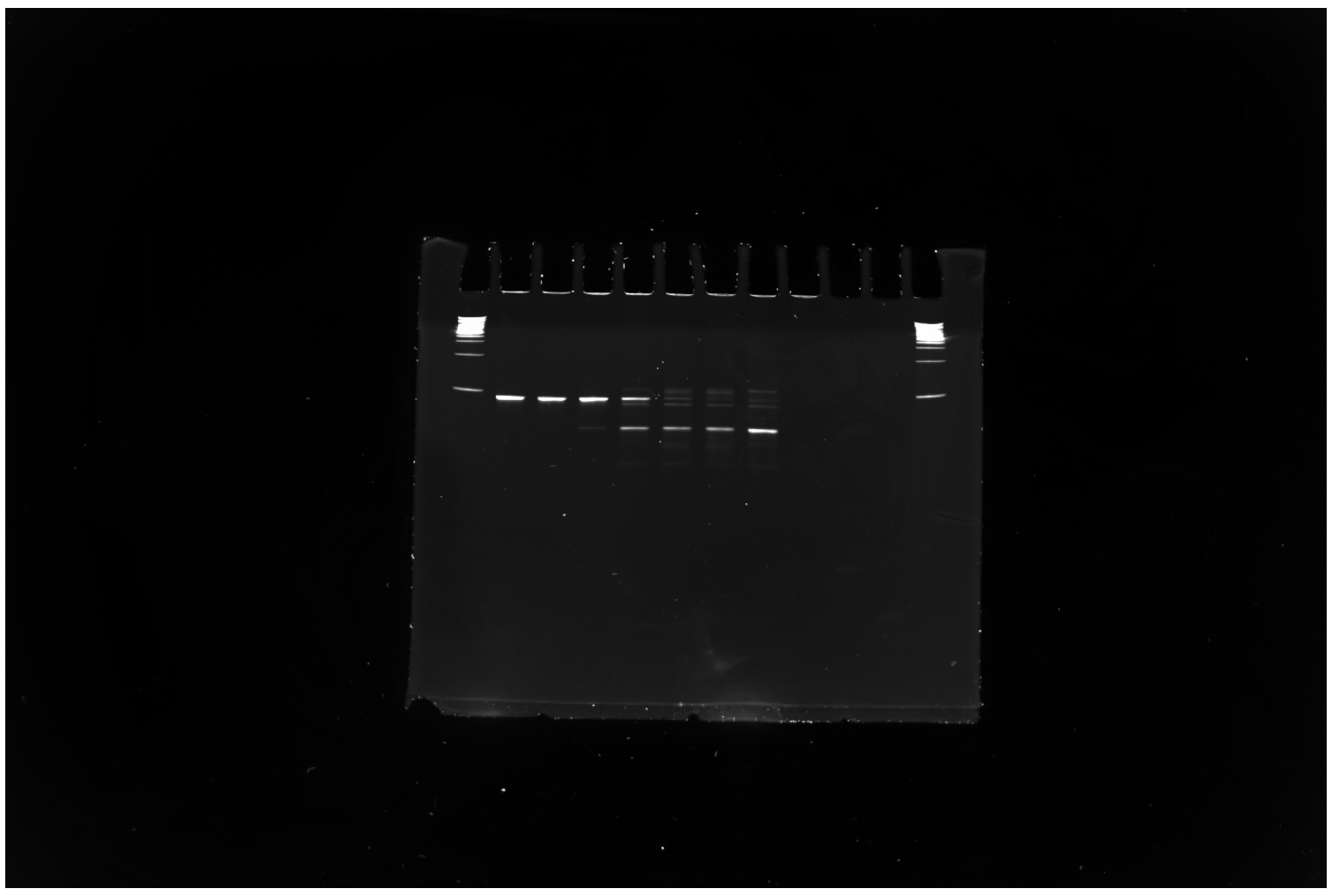

Supplement: Supplementary file 1 [file 44460_2026_134_MOESM1_ESM.pdf]
