# Supplementary material for: Base gap switches as transcriptional regulators and their applications as biosensors
Source: Nat Sens. 2026 Sep 14;1(10):956–66. doi: 10.1038/s44460-026-00134-z (PMC13642041; doi:10.1038/s44460-026-00134-z)

nature portfolio

Peer Review File

Base Gap Switches as Transcriptional Regulators and Their Applications as Biosensors

Corresponding Author: Dr Daniel Richards

Version 1:

Reviewer comments:

Reviewer #1

(Remarks to the Author)
The manuscript presents the development of a novel transcription-based biosensor, termed the Base Gap Switch, which operates via a distinct mechanism involving a deliberately inserted transcriptional “gap” that can be sealed in the presence of a target nucleic acid. The system leverages DNA ligase–mediated gap closure to enable RNA polymerase transcription, resulting in robust protein expression. The approach is positioned as an alternative to toehold switches and riboswitches, with key advantages including minimal background leakage, high specificity, adaptability to multiple targets, and compatibility with both ssDNA and dsDNA triggers. The authors demonstrate the versatility of the Base Gap Switch across three proof-of-concept diagnostic applications: (1) RPA-IVTT–based ultrasensitive detection of Trichomonas vaginalis. (2) One-pot RPA format for simultaneous detection of Neisseria gonorrhoeae and Chlamydia trachomatis. (3) SNV detection of EGFR T790M down to 0.5% variant allele frequency, exploiting ligase selectivity. The concept is innovative, and the data support the potential for translation into low-cost, high-specificity nucleic acid diagnostics. However, several technical and mechanistic issues require clarification or further experimental support. I therefore recommend a major revision to address the concerns outlined below before the manuscript can be considered for publication.

1. Mechanistic Basis of Background Leakage
While Figures 1C–E suggest that RNAP cannot bypass the gap in the absence of target, some datasets—particularly with TSS-type constructs—display measurable background signal. The manuscript does not clarify under what conditions RNAP might traverse the gap. A systematic study examining the influence of gap length, position, internal sequence composition, and potential secondary structures on background leakage would substantially strengthen the mechanistic claims.
2. Reduced Efficiency with dsDNA Targets
In Figure 2D, dsDNA triggers elicit lower reporter expression compared to ssDNA triggers. Although the authors frame this as evidence of dsDNA compatibility, the observed lower efficiency is not explained. As many diagnostic targets exist in dsDNA form, reduced sensitivity could limit practical application. Potential factors such as target-binding site accessibility, hybridization kinetics, or sequence stability should be investigated to identify and potentially mitigate this limitation.
3. Clinical Validation of the One-Pot Format
For translational relevance, the one-pot (amplification + IVTT) approach should be evaluated using actual clinical samples and benchmarked against a standard NAAT. A side-by-side comparison of one-pot versus two-step formats with statistical analysis of diagnostic performance (sensitivity, specificity, LOD) would significantly strengthen the manuscript’s clinical applicability claims.
4. Background in Multiplex Detection (Figure 4C)
The CT channel in the NG/CT multiplex assay exhibits elevated background, potentially impacting multi-target assays involving three or more analytes. While the authors attribute this to leakage from the AciI-regulated Base Gap Switch, the specific cause—whether nonspecific activation or another factor—remains undefined. Additional experiments to pinpoint the leakage mechanism and demonstrate an effective mitigation strategy with improved performance are warranted.
5. Specificity Limitations in SNV Detection (Figure 5C)
While the system discriminates A and G variants effectively, C and T variants show partial activation with non-complementary triggers, reducing specificity. The authors speculate on Taq DNA ligase selectivity and transcription efficiency differences but do not provide systematic analysis. For the technology to be broadly applicable to SNV detection, a generalized design framework addressing mismatch position, type, and ligase behavior should be developed and experimentally validated.
6. Trigger Length Effects (Supplementary Figure 4)
The reported trend of decreased performance with shorter triggers is not quantitatively analyzed. Specifically, 3′ truncations abolish activity (presumably due to ligation failure), while 5′ truncations are more tolerated. Without quantitative correlation between trigger length, binding affinity, and expression efficiency—including consideration of melting temperature and mismatch position—these observations cannot serve as a robust design guideline.

7. Orthogonality Testing in Multiplex Contexts (Supplementary Figure 7)
Orthogonality experiments were conducted in pairwise conditions only, which does not fully replicate competitive environments in true multiplex assays. Additional experiments involving three or more targets are needed to assess possible competition, trigger interference, or false positives in complex mixtures. Quantitative analysis under such conditions would provide greater confidence in the system's multiplexing capability.

Reviewer #2

(Remarks to the Author)
The authors report on a novel class of programmable transcriptional regulators, which can be selectively actuated by DNA, terming these regulators Base Gap switches. The authors exemplify the switches to specifically control expression with an ability to discriminate single nucleotide mismatches. The orthogonality of the approach is shown via the expression of the mNeptune, mPapaya, and mSirius fuorescent proteins and further including the expression of Nanoluciferase. The authors demonstrate the potential of these gene-circuit based biosensors with several practical and diverse applications. Combining the Base Gap switches with isothermal amplification, the authors achieved the ultrasensitive detection of Trichomonas vaginalis, validating the approach with real clinical samples, highlighting the true life application of the developed assay. Furthermore, the authors demonstrated the approach for the detection of Chlamydia trachomatis 16s rRNA and Neisseria gonorrhoeae porA.

Very minor revisions are required. The introduction is extremely well written and very well positions the novelty of the work reported. The experimental is easy to follow and very well detailed.
However, non-scientific phrases such as "Gratifyingly, orthogonality was maintained" should be removed from the text. I personally find some of the figures too loaded with information and would suggest moving some of the content of the figures to the SI so that the details can be better seen, but I do not insist on this – it is a personal choice.
Some questions:
Can the authors suggest why the mNeonGreen expression levels are reduced for dsDNA?
What was the rationale for targeting the actin gene of Trichomonas vaginalis and not a gene specific for TV?

Can the authors offer an explanation as to why in the Chlamydia trachomatis and Neisseria gonorrhoeae work leakage from the Base Gap switch was observed and why this would result in a a lowering of the expression of AciI? I know the authors offer a general discussion as to the reasons for leakage but further discussion regarding the expression of AciI would be useful.

Why were no real samples analysed for Chlamydia trachomatis and Neisseria gonorrhoeae?

Are the authors confident about the claim "To the best of our knowledge, these results represent the first demonstration of SNV discrimination using a strictly transcription-based biosensor." This is a sweeping statement.

Overall, an excellent piece of work – I congratulate the authors, very innovative, with multiple applications demonstrated. Extremely well written manuscript and a piece of work that will significantly contribute to the state of the art. I thoroughly enjoyed reading this manuscript and believe it will be appreciated by the readers of Nature Sensors and highly recommend it for publication.

Reviewer #3

(Remarks to the Author)
Partington and colleagues report a useful new strategy to control transcription of a downstream gene using molecular sensors that they call base gap switches. Base gap switches detect the presence of a target DNA by having it hybridize to a region in the 3' portion or just downstream of a T7 promoter sequence, enabling ligation of the target to the promoter fragment to activate transcription of the downstream gene. Partington et al. make use of cell-free transcription-translation systems to read out status of their sensor template, employing either fluorescence or luminescence signals for assay output. By making transcription and downstream translation dependent on the ligation state of the DNA template rather than the folding state of RNA or DNA, the base gap switches provide relatively low leakage signals in their reactions. The authors integrate their switches with RPA isothermal amplification to reach clinically relevant limits of detection. The base gap switches can also be used to detect point mutations in certain conditions.

Overall, this system represents a nice advance for cell-free sensor diagnostic technologes. The mechanism where T7 RNA polymerase ostensibly transcribes through a nick in the template strand with good efficiency is also unexpected and could lead to other novel sensor designs. However, the manuscript as it stands has a number of critical weaknesses that should be resolved before publication. These issues range from problems with the asserted limit of detection to multiple exaggerations comparing the technology to others. The system also has several major limitations that should be discussed in the manuscript. Specific points are below:

1. The template switching reference (Ref. 37) shows a decrease in downstream transcription through a nick site and sequence-dependent template-switching efficiency; however, there is no mention of these effects in the manuscript. How did

these affect base gap switch performance? Were particular sequence rules identified for optimal targets?

2. The paper is almost completely lacking in p-value calculations for statistical significance and the graphs do not show individual data points. This information should be added to the manuscript.

3. The ability of base gap switches to detect down to 10 fM of target without pre-amplification is cited as a key feature of the system. However, Figure 5D(i) where this parameter was determined indicates that the analysis was flawed. The measurements at 100 fM and possibly 1 pM indicate that the differences between the SNV and the mutant are not statistically significant (plotting individual points would help with this assessment). Consequently, it is unlikely that the measurement at 10 fM is correct and the true limit of detection is 2 to 3 orders of magnitude higher. This LoD analysis should be redone with p-value calculations and plotting of the individual data points.

4. The manuscript transitions into systems with luciferase outputs and reports a rate of change of the luminescence signal but little detail is given. What are the units of Maximum dRLU/dT? Why is T capitalized, if it is presumably a time derivative? At what time is each Maximum dRLU/dT value determined? Are the Maximum dRLU/dT for each target concentration replicate taken at the same time? The RLU vs. time plots should be provided as supplementary information for the limit of detection experiments.

5. A major limitation of the system is its combination of transcription-based readout and use of exposed target binding site. Based on the system design, an off-target sequence that has 5' and 3' ends that match the target sequence with a sufficiently high melting temperature and without a terminator sequence will probably activate the base gap switch. The authors should report in the paper the output of their system using an off-target sequence that has 5' and 3' ends generated from the RPA primers used in their assays, and using the appropriate amplicon strand polarity to bind to the TBS in the switch. This test case is a critical one to include in the paper since isothermal amplification techniques are known to have non-specific amplification. These spurious amplicons will often have 5' and 3' ends generated from the amplification primers, which could thus activate the base gap switches.

6. The ability of the base gap switches to detect dsDNA as described by the authors is unexpected. Complete strand invasion of the single-stranded TBS into a fully double-stranded target would be a very slow reaction without thermodynamic favorability. Have the authors confirmed that the signal is not due to free single-stranded amplicons generated during RPA or PCR?

7. The SNV detection scheme has major limitations that will constrain practical utility. Since the system requires the SNV to be located near the ligation site, many amplification methods will not be compatible (since the mutation will often be in the primer binding site) and the amplicon will probably have to be treated in some way to ensure the mutation is near the end. The authors should demonstrate how their SNV detection method can be made compatible with a general amplification scheme, or they should at least present a putative application where an amplification-free assay with a putative LoD of 10 pM could be valuable.

8. The authors have at least three novelty statements in the paper that are incorrect:

a) “To the best of our knowledge, these results represent the first demonstration of SNV discrimination using a strictly transcription-based biosensor." However, as a counterexample, Yoon et al., Biosensors and Bioelectronics 208, 114221 (2022) reports a transcription-based sensor with SNV specificity. Furthermore, the category of "transcription-based biosensor" could encompass many other assays that rely on the availability of a functional transcription template. For instance, SHERLOCK assays rely on a transcription template to produce an RNA for the reaction and they also provide SNV specificity.

b) The "first SGC-based assay to achieve an LOD lower than 100 copies without digital discretisation (e.g., CATCH28)." However, in Ref. 53, Figure 1 shows detection down to 1 molecule per µL, which corresponds to an LOD below 100. Ref. 53 is not the only paper that reports SGC-based assays with <100 copies either.

c) “In our orthogonality test of seven Base Gap switches, we observed an average crosstalk of just 0.89% and a maximum crosstalk of 31.26% (Figure 4A, Supplementary Figure 7). These values drop to just 0.16 and 3.1%, respectively, when we eliminate the combination of CSS-1 and Trigger 3, which was specifically designed with a high degree of paired identity (90%)..... Thus, our data suggest that Base Gap switches may be more orthogonal than traditional toehold switches." The original Green et al. paper describing toehold switches states the following: "For example, the set of 18 switches with less than 2% crosstalk has a reciprocal crosstalk threshold of 50. This metric corresponds to the minimum output dynamic range to expect in a network employing this library of 18 switches." Partington et al. are thus showing a higher maximum crosstalk level of 3.1% vs. 2% using only 6 switches compared to 18 for Green et al. Thus, this novelty claim also does not hold.

9. The Methods section describes the base gap switch as being run three times through a DNA NucleoSpin Gel and PCR Clean-up Kit. How important is this repeated procedure? Did the number of purification times affect the leakage levels of the sensors? Were the sensors purified through gel extraction? If so, what was the yield of the final purified base gap switches?

10. More information needs to be provided for the stability testing shown in Supplementary Figure 3. Did the reaction subjected to long-term storage contain the enzymes required for the assay or only the DNA components? Since DNA should be very stable at room temperature over 8 weeks, the stability test is only really noteworthy if it is done with the ligase present.

11. The manuscript should also report the number of copies of target per µL the system can detect. The current version of the paper lists the total number of target copies detected, but that metric is not very informative without the volume used. Volumes used in the RPA reactions should also be provided since the manufacturer instructions may change.

12. In Figure 1C, it is not possible to see the — trigger bars. The graph should be updated to make sure these can be seen.

13. Figure 1 should be updated to make it clear that the region where the trigger binds contains a promoter, otherwise the mechanism of the system is unclear.

14. Line 51: "assa y" should be "assay"

Version 2:

Reviewer comments:

Reviewer #1

(Remarks to the Author)
As the authors have adequately addressed all reviewers' comments, I recommend that this manuscript be accepted after minor revision.
The manuscript would benefit from the inclusion of additional relevant and recent references to further strengthen its scientific context and impact.
In particular, the authors are encouraged to consider incorporating recent studies in this field, such as those reported in doi.org/10.1016/j.bios.2024.116517, doi.org/10.1016/j.cej.2023.142614, doi.org/10.1016/j.bios.2022.114221, as well as other closely related recent works, to further enhance the discussion and positioning of the work.

Reviewer #2

(Remarks to the Author)
I have now thoroughly read the extensive response to all reviewers, and really appreciate the immense effort the authors have made to address the queries and comments of the reviewers. The revised manuscript is very much improved and many questions have been resolved - of course, with such highly innovative work, there are some open questions that will form the basis of future work. I was very impressed by the original manuscript - but I do believe the issues raised by all reviewers were valid, and responding to the and incorporating the suggested changes, and adding further information, has truly contributed to a significant enhancement in the quality of the manuscript. I happily recommend the manuscript be accepted for publication and congratulate the authors on an excellent piece of highly innovative, though-provoking and interesting work, that will certainly notable contribute to the state of the art.

Reviewer #3

(Remarks to the Author)
Partington et al. have addressed some of the concerns raised in the previous version of the manuscript. However, there are still problems with the paper in terms of data analysis, description of experimental procedures, and seemingly contradictory results. I do not recommend publication of the paper in its current form:

1. RPA–IVTT assays: More information should be provided on the reactions rather than referring readers to the manufacturer's instructions, which may change or become unavailable. What concentration of the RPA primers was used? What was the volume of the other components? The current TwistAmp Basic Kit does not use 25 µL as the recommended volume. What was the volume of the PUREfrex reactions and the volumes of the different components?

2. The section "Trichomonas Vaginalis patient samples" does not provide an acceptable description of the methods used. "The collection and processing of swab samples are described in the AVEONS study": I have checked the referenced paper and the main text does not describe any methods for PCR sample preparation. How were samples recovered off the swabs? Were the samples subjected to any DNA/RNA extraction prior to use? When were these samples obtained from patients?

3. For the paragraph starting on line 319, were the complementary strands of the dsDNA pre-annealed before adding to the reaction?

4. The authors should describe exactly what was calculated for the "Gibbs free energy of mRNA hairpin formation".

5. For Figure 3B, the LoD determination should be better explained/justified. The authors seem to be basing the LoD determination on the separation from the dotted line. The dotted line is probably the mean of the max. dRLU/dt measurement for the 0-copy control experiment (I cannot find a description of it in the caption or main text); however, that control measurement should have its own standard deviation, which is not included in the plot. The Methods section states that "limits-of-detection (LoD) are reported as the mean of the negative ± two standard deviations", but it is unclear which standard deviations are used. The LoD determination must include the variation in the measurements of both the sample and the negative control, but the authors do not appear to be doing that in the paper. The 6 copies/µL measurement seems

to have multiple measurements that fall very close to the 0-copy control line, which suggests that the effective LoD could be higher than 6 copies/µL if the variance in negative control was properly taken into account.

6. The authors on line 414 state that "we were still able to detect 12 copies / µL of both targets and observed no significant crosstalk." However, this statement does not match well with the experimental data in Figure 4C. The leakage signal from the CT switch in Figure 4C(i) appears quite significant and increases with the concentration of the non-cognate NG porA target. In particular, the 10^5 copies of NG porA generates a signal that overlaps with that of the dotted line, which the authors state represents the LoD of NG porA "defined as the mean of three negatives (no target) ± two standard deviations" (+ two standard deviations would be more appropriate since there is only 1 line). It seems quite likely that this crosstalk signal could lead to false positives at higher NG porA levels. Can the authors also explain why the LoD line from the negatives is significantly higher than the signals from the lower NG porA levels (≤ ~10^3 copies)? Why would lower concentrations of NG porA cause suppression of output of AciI?

7. For line 316: "These optimised Base Gap switch-controlled genes remain stable in solution at both 21 °C and 4 °C for at least eight weeks (Supplementary Figure 5)": The authors should explicitly state that this test was done using DNA alone and that any enzymes and in vitro transcription/translation components were added later, and presumably stored at low temperature prior to the test. The current language is somewhat ambiguous and could lead readers to assume the system was tested with components that are known to be more susceptible to degradation/denaturation at higher temperatures. It is not unexpected for DNA constructs/primers/plasmids to remain stable for weeks at room temperature.

8. For the cfDNA experiments, the authors should explain the source of the cfDNA samples and how they were obtained and processed for the assays.

9. For Figure 5D(i), the authors have chosen to retain their claim that the detection limit of the assay for T790M DNA is 10 fM. The 100 fM (0.1 pM) measurements clearly overlap with the LoD line on the plot. If the true LoD is 10 fM, then the 100 fM data should also be clearly above the LoD line. Also, following the earlier comment, the dotted line should not be used for determination of LoD because it does not seem to reflect the variance in the negative measurements.

10. For Figure 5D(ii), how much total DNA was added to the reactions?

11. For Supplementary Figure 10B, more information on the experiment should be provided. Were the triggers supplied as ssDNA or dsDNA? Did they have 5' phosphate groups? Was ligase used in the reactions? The absence of a strong signal seems to contradict other experimental data in the paper. In orthogonality study using 50-nt triggers, a non-cognate trigger with 90% sequence match to the cognate trigger demonstrated 30% crosstalk. Thus, two 30-nt regions with 100% sequence match should be able to hybridize to the template and be ligated. Moreover, in Figure 1C, 3' elongated and 5' elongated triggers can still generate gene products, which demonstrates that T7 RNAP can continue transcribing even when the templates are not base paired. The authors should describe in the Discussion how the results in Supplementary FIgure 10 can be reconciled with the potentially contradictory data from other experiments. Sequences for the experiments in Supplementary Figure 10B should also be provided. They do not seem to be present in the Supplementary Tables spreadsheet or on the chemRxiv version of the tables (https://doi.org/10.26434/chemrxiv.15001046/v1).

11. Methods section: How long does the ligation step before the IVTT reaction take?

12. Line 186: "The RBS scores for both start positions encoding a functional protein (+85, +87)": Are these positions correct?

13. In Supplementary Table 1, none of the RPA primers are shown as having 5' phosphate groups. Are these present in the primers? If not, how is transcription affected by the presence of a nick in the template strand?

Version 3:

Reviewer comments:

Reviewer #3

(Remarks to the Author)
The authors have addressed most of my previous concerns. However, I cannot confirm all the changes have been made since they did not upload the supplementary tables again and the supplementary information was also not uploaded. There are still issues with the manuscript that should be addressed before publication:

1. The author's use of the dashed line to make LOD determinations is not sufficiently transparent and does not allow readers to see how reasonable the LOD measurements are. To avoid this issue, the authors must include the data points they measured for their NTC experiments on their plots rather than having the dashed line on its own. Simply including this important information would remove the uncertainty surrounding their LOD determinations. Indeed, Figure 3C already includes the NTC measurements and it is clearly helpful in identifying the false-negative measurements. The NTC measurements should be included at minimum in Figure 3B, Figure 4C(i and ii), and Figure 5D(i). Also, individual data points should be spread out more laterally so that it is easier for readers to discern data variability. This is particularly a problem for Figure 5D(i) and Figure 3B, even though the points are spread out laterally for a very similar plot in Figure 4C.

2. Regarding the revision to the stability testing description (line 285-286), it is inappropriate to only mention in the caption of

the supplementary figure that enzymes were not involved in stability testing. Readers could assume from the main text that room-temperature stability was actually validated for the sensors. The main text must state that all other components (enzymes, PURExpress, etc) were stored at -20 °C prior to use in the experiment.

3. The use of the term "in situ" regarding the ligation step is unclear. In a biological context, this usually means in a biological sample or specimen. Language like a one-pot or all-in-one ligation/IVTT reaction would be clearer.

4. Use of positions +85 and +87 for RBS calculator translation start site is still perplexing. +85 and +87 only differ by 2 nt so they would not be in the same reading frame.

5. The results from Supplementary Figure 10B make more sense with the sequence information and knowing there is no 5' phosphate and there is a nick in the template. However, it seems unlikely that transcriptional termination is occurring because of mismatched base pairing since T7 RNAP can transcribe through DNA duplexes. Furthermore, it is well known that T7 RNAP can efficiently transcribe on ssDNA after transcription initiation from a dsDNA promoter, so termination in the mismatched region is unlikely. It seems more likely that the observed behavior is from the effect of the transcriptional bubble from the active RNAP. The bubble draws the distal portion (5' end) of the target away from the complementary portion of the sense strand. With the mismatched middle region of the target not bound to the sense strand, there will probably be a greater distance that the RNAP must jump for template switching, which lowers transcriptional efficiency into the downstream region.

6. It would be very helpful to readers if the authors could clarify their figures. Some marker to indicate if a strand is phosphorylated would be useful, and some difference in the diagram to indicate whether strands are ligated or nicked would avoid confusion. For instance, in Figure 3A, the diagram does not make it clear that the distal portion (5' end) of the target is not ligated while the other end would be. At present, readers would be forced to look into the supplementary tables to get most of this essential information.

7. In Figure 3C(i), the size of the error bars do not seem to match the spread of the individual data points (e.g. Samples 1-5, 8).

Version 4:

Reviewer comments:

Reviewer #3

(Remarks to the Author)
The authors have done a good job responding to my questions and critiques. Given the performance of the sensors and their novel/surprising operating mechanism, I support publication in Nature Sensors.

Open Access This Peer Review File is licensed under a Creative Commons Attribution 4.0 International License, which permits use, sharing, adaptation, distribution and reproduction in any medium or format, as long as you give appropriate credit to the original author(s) and the source, provide a link to the Creative Commons license, and indicate if changes were

made.
In cases where reviewers are anonymous, credit should be given to 'Anonymous Referee' and the source.
The images or other third party material in this Peer Review File are included in the article's Creative Commons license, unless indicated otherwise in a credit line to the material. If material is not included in the article's Creative Commons license and your intended use is not permitted by statutory regulation or exceeds the permitted use, you will need to obtain permission directly from the copyright holder.
To view a copy of this license, visit https://creativecommons.org/licenses/by/4.0/

Eidgenössische Technische Hochschule Zürich
Swiss Federal Institute of Technology Zurich

Dr Daniel A. Richards
Senior Scientist
daniel.richards@chem.ethz.ch
+41 44 633 41 92

ETH Hönggerberg HCI F 111
Vladimir Prelog Weg 1
CH-8093 Zurich
Switzerland

Dr Olga Bubnova, Dr Frank Sun, Ms Roman Doumbia
Nature Sensors

20.03.26

Dear Dr Bubnova, Dr Sun, and Ms Doumbia,

First and foremost, I would like to thank you for granting us the opportunity to resubmit our manuscript "Base Gap Switches as Transcriptional Regulators and Their Applications as Biosensors" to *Nature Sensors*. While the three Reviewers for this manuscript were broadly positive about the innovative nature of our work, two requested revisions, and you asked that we resubmit after we had made substantial efforts to address their concerns. **To achieve this, we have performed an extensive campaign of experiments, comprising over 30 additional experiments. This has resulted in one entirely new manuscript Figure, changes to three other manuscript Figures, and eight new Supplementary Figures.**

A primary concern of the Reviewers was the absence of guidelines for designing Base Gap switches. This stemmed from a lack of understanding of the structure–function relationships. To remedy this, we have performed an extensive structure–function analysis, systematically varying the structural features of 65 Base Gap switches and studying their ability to modulate expression (**Figure 2, Supplementary Figures 7 & 8**). From this, we have developed a set of rules for designing Base Gap switches for specific targets: "*.......high RBS scores (<1000 should be discarded) and 2) low Gibbs free energy of mRNA hairpin formation. 3) For general targets, a short (five nucleotide) spacer should be included between the promoter and the TBS (target-binding site)*".

Another concern shared by the Reviewers was that we did not fully elucidate how the structural features of Base Gap switches affected single-nucleotide variant (SNV) detection. We have now expanded our study into SNV detection to include how mismatch position (**Figure 5B**) and mismatch type (**Figure 5C**) impact SNV discrimination, and also determined that this is both ligase (**Supplementary Figure 15**) and transcription (**Supplementary Figure 19**) dependent. From this, we now have a more complete picture of SNV discrimination using Base Gap switches, and can put forth more robust design rules:

"*For SNV detection, no spacer should be employed, with the mismatch placed directly after the nick in the +2 position.*"

In addition to these fundamental mechanistic studies, we have also repeated multiple analyses with larger *n* numbers to facilitate statistical analysis (**Figure 3B**, **Figure 5D$_{ii}$**), performed additional cross-reactivity studies of our switches to further demonstrate orthogonality and multiplexing potential (**Supplementary Figure 12D**), and performed control studies to rule out non-specific activation of the switches (**Supplementary Figure 10**). We have also removed specific novelty claims related to SNV detection, and thank the Reviewers for pointing these out.

To summarise, by conducting additional experiments and extensively revising the manuscript, we have addressed virtually all 36 of the Reviewers' comments. **In doing so, we have substantially improved the work.** We now have a more thorough understanding of how Base Gap switches work, which has enabled us to develop a set of design rules that will allow others to adopt this technology. This will greatly facilitate its spread into the community and bolster its impact.

A full point-by-point response to the Reviewers is included below. We hope that you will consider these changes sufficient to warrant sending our manuscript back to the Reviewers for a second evaluation. If you have any questions, please do not hesitate to contact me.

Yours sincerely

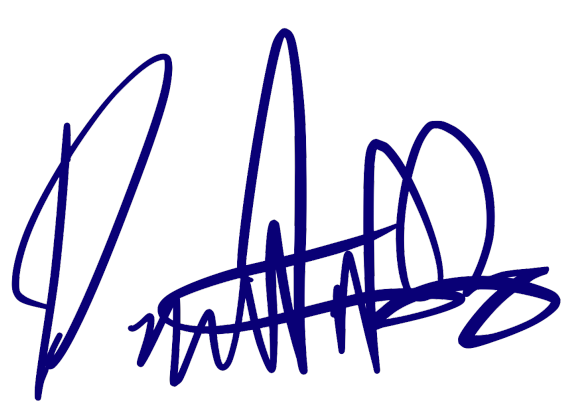

Dr Daniel A. Richards

Sent on behalf of Yukina Partington, Beatrice Dalla Via, Fabrice Abgottspon, Isaure de Geyer, Piet Cools, and Andrew J. DeMello

Response to Reviewer 1

Thank you for taking the time to review our manuscript and provide some feedback. We are pleased that you found the work innovative and could see the potential for impact. In addressing your constructive critiques and comments, we believe that we have significantly improved the quality of the work. We provide a point-by-point response below.

1. Mechanistic Basis of Background Leakage

While Figures 1C–E suggest that RNAP cannot bypass the gap in the absence of target, some datasets—particularly with TSS-type constructs—display measurable background signal. The manuscript does not clarify under what conditions RNAP might traverse the gap. A systematic study examining the influence of gap length, position, internal sequence composition, and potential secondary structures on background leakage would substantially strengthen the mechanistic claims.

This is certainly true; previously, we did not fully understand the structure–function relationships governing Base Gap switches. We have now reworked the manuscript to include a figure (and two supplementary figures), and an associated discussion, dedicated to this (**Figure 2, Supplementary Figure(s) 7 and 8**). Based on these results, we now understand that the performance differences between Base Gap switches are driven primarily by how their structure impacts translation rates. Stronger mRNA hairpins lower translation efficiency, leading to lower rates of expression. We also found that longer spacers between the T7 promoter and shorter Base Gaps led to increased leakage, suggesting that RNAP is better able to traverse smaller gaps when given more opportunity to form stable transcription complexes. We will continue to explore this in our lab, as we feel lowering the leakage is key to getting the most from these switches.

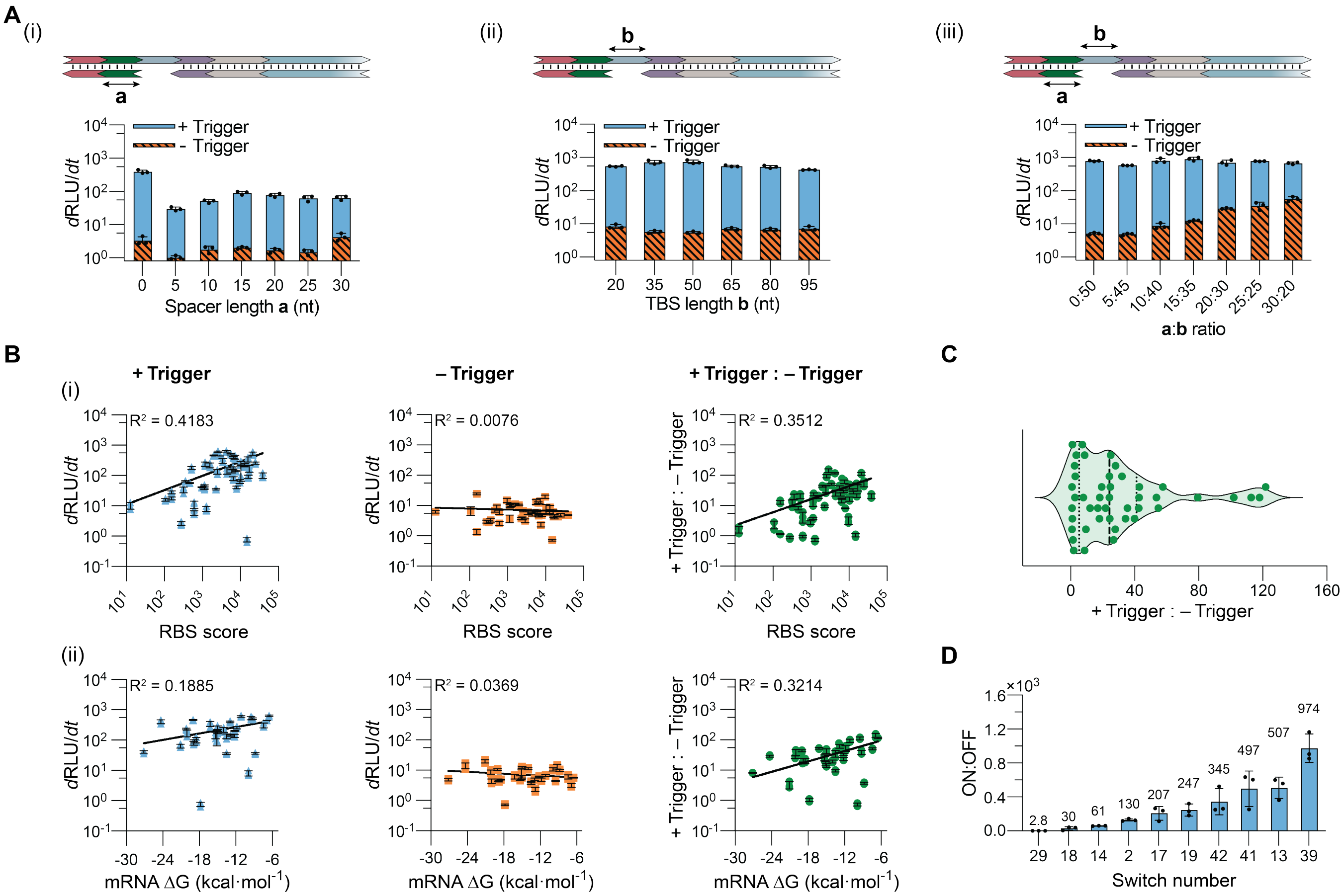

Figure 2. Structure–function relationships. A) NanoLuc Luciferase (Nluc) expression as a function of (i) the number of base pairs between the promoter and the TBS (a), (ii) the TBS length (b), and (iii) the ratio of the the promoter–TBS gap length and the TBS length (a:b) A gap of 5 basepairs is optimal, combined with a high a:b, though expression yields are invariable to TBS length. **B)** Nluc expression from 45 Base Gap-switch controlled SGCs with varying TBS compositions as a function of (i) RBS score and (ii) the Gibbs free energy of formation for mRNA hairpins. Data shows expression in the presence (+ Trigger, blue triangles) and absence (– Trigger, orange squares) of triggers, as well as the ratio of the two (+ Trigger : – Trigger, green circles). Switch performance is positively correlated with increasing RBS scores and decreasing hairpin stability. Switches with an RBS score <1000 (red triangles) were not included in subsequent analyses. **C)** A violin plot of the + Trigger : – Trigger values. **D)** mNeonGreen expression from a selection of switches, from low to high ON:OFF. The numbers above the bars represent the ON:OFF ratio. All plots with error bars represent the mean ± standard deviation. The numbers represent R^2 values were obtained from a semi-log fit of the data.

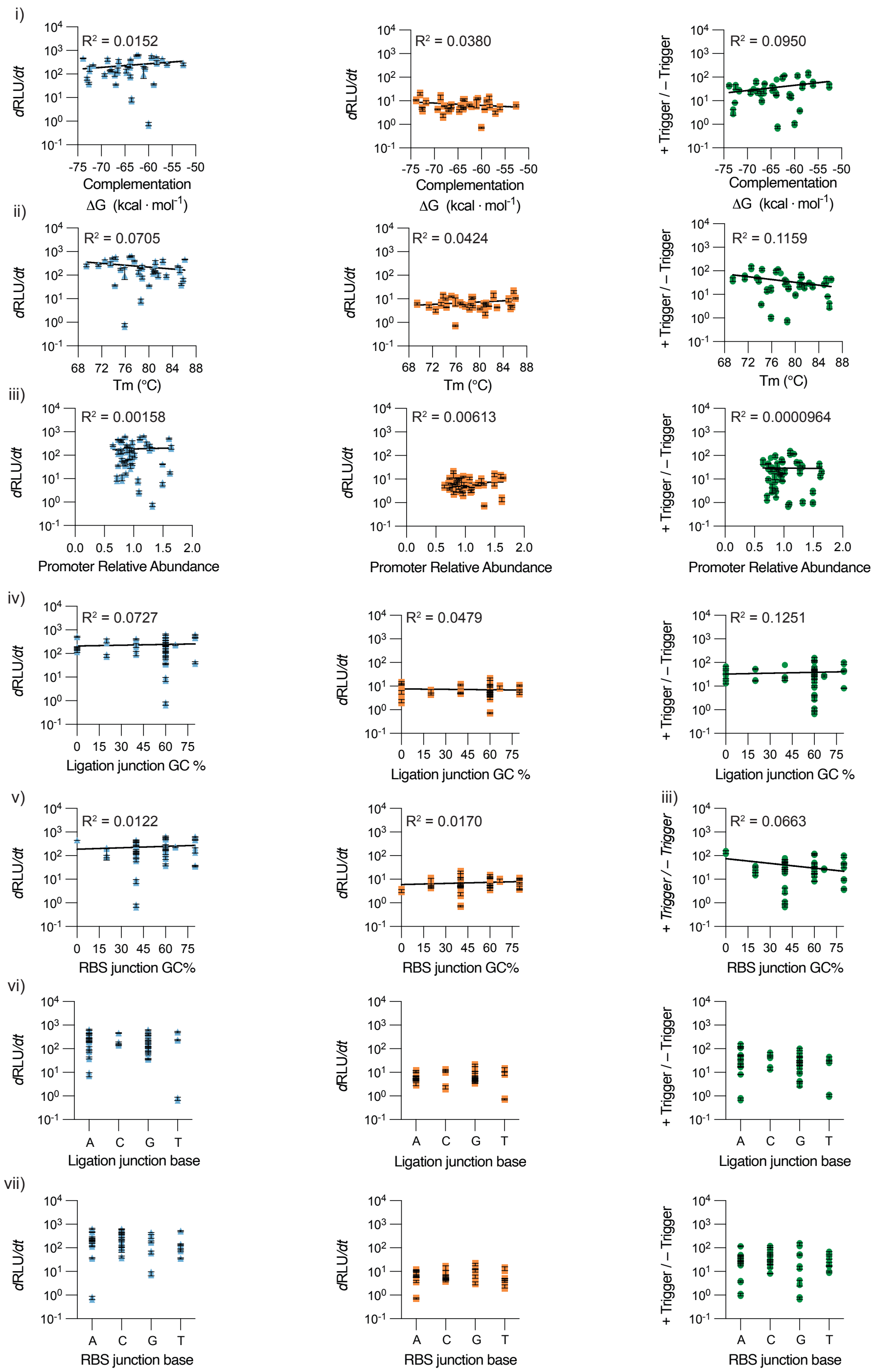

Supplementary Figure 7. NanoLuc Luciferase (Nluc) expression from 45 Base Gap-switch controlled SGCs with varying TBS compositions as a function of (i) the Gibbs free energy of complementation, (ii), the melting temperature (TM) of complementation, (iii) relative promoter strength, as determined by Conrad *et al.*, [1](iv) GC% of the five bases after the ligation junction, (v) GC% of the five bases before the RBS junction, (vi) the nucleotide at the ligation junction, and (vii) the nucleotide at the RBS junction Data shows expression in the presence (+ Trigger, blue triangles) and absence (– Trigger, orange squares) of triggers, as well as the ratio of the two (+ Trigger : – Trigger, green circles). Error bars represent the mean ± one standard deviation.

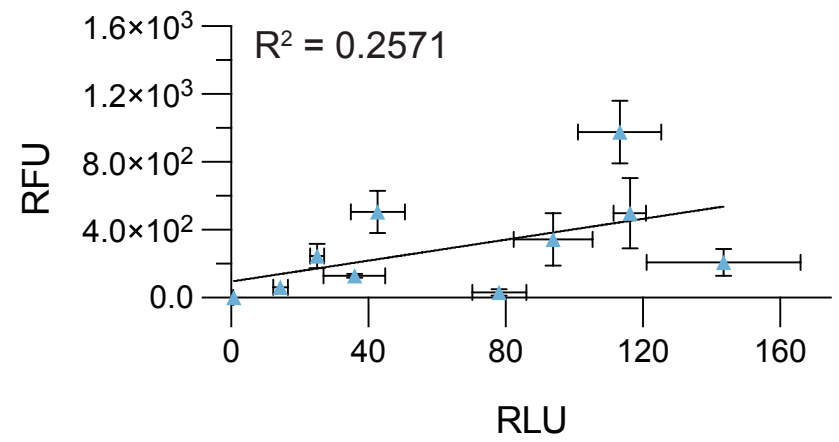

Supplementary Figure 8. Correlation between NanoLuc Luciferase (Nluc) and mNeonGreen expression when the same Base Gap switches are employed. Error bars represent the mean ± one standard deviation.

We would like to note that we have decided to omit detailed discussion of template strand switches (TSSs) in this manuscript. The majority of the manuscript focuses on CSS switches (even more so with the additional figures), so we felt the discussion of the TSS switches distracted from the message. A single comparison is now made in **Supplementary Figure 2**.

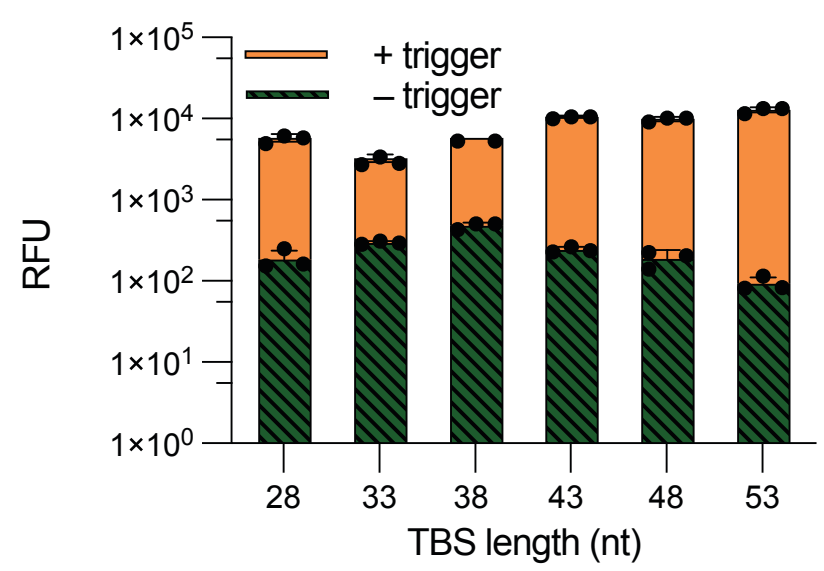

Supplementary Figure 2. Relative mNeonGreen fluorescence from a gene controlled by Base Gap switches with gaps on the coding strand. Error bars represent the mean ± one standard deviation

We have added the following text to the Results section:

"To inform the rational design of Base Gap switches, we explored how their structure impacts their ability to control expression (**Figure 2**). Given the low levels of leakage observed with Base Gap switch-controlled mNeonGreen genes, we performed these studies using a NanoLuc Luciferase (Nluc) reporter to ensure an observable signal.

We first optimised the spacer between the T7 promoter and the TBS. To minimise any bias in relative expression rates,[43] we kept the five bases (GGGCC) immediately after the promoter and the composition of the TBS constant (**Figure 2A$_{i}$**). This sequence was chosen due to its positive influence on transcription rates.[43] We achieved the best performance with a five basepair spacer and observed no apparent relationship between spacer length and expression levels. Using this optimised spacer, we varied the TBS length, extending at the 3′ termini to avoid altering the composition near the sensitive promoter–TBS junction (**Figure 2A$_{ii}$**). No significant relationship between TBS length and expression levels was apparent, suggesting Base Gap switches can detect targets of various lengths. Increasing

the length of the spacer while decreasing the length of the TBS led to a significant increase in switch leakage (– Trigger) (**Figure 2A$_{iii}$**). This suggests that when short TBS lengths are employed, shorter spacers decrease leakage and improve performance.

To better guide the selection of TBSs, we created a library of 50nt Base Gap switches with varied TBS compositions and monitored their control over Nluc expression (Figure 2B, Figure 2C). We correlated the performance of these switches against multiple structural and thermodynamic parameters (**Supplementary Figure 7**), ultimately finding the best predictors of performance were RBS Score (R^2 = 0.4183) (Figure 2B$_i$), as calculated using the Salis Lab RBS calculator,[34,35] and the Gibbs free energy of mRNA hairpin formation (R^2 = 0.3214) (**Figure 2B$_{ii}$**). Interestingly, we observed no discernible trends when studying expression in the absence of trigger (– Trigger). Furthermore, we found no correlation between switch performance and relative promoter strength, Gibbs free energy of trigger–TBS complementation, or basepair composition at the ligation or RBS junctions. The lack of correlation with basepair composition is particularly noteworthy, since prior reports suggest that the capacity of T7 RNAP to traverse gaps or nicks, is highly sequence-dependent.[41] This suggests that the differences in expression control across Base Gap switches are largely attributable to their relative impacts on translation, not transcription. This concurs with previous findings that translation is the rate-limiting step in IVTT,[42] and has a greater impact on relative protein expression.[44]

Due to the rapid conversion of the furimazine substrate at high trigger concentrations, causing signal saturation, we were unable to determine true ON:OFF ratios for the Base Gap switches in the Nluc library. To address this, we selected ten switches, ranging between low-performing to high-performing, and installed them onto an mNeonGreen gene. These switches exerted strong control over mNeonGreen expression, with ON:OFF ratios ranging from 2.8 ± 0.25 to 973 ± 167 (**Figure 2D**). The highest of these ratios are significantly greater than toehold switches coupled to GFP reporters, which have not exceeded ~150:1 in IVTT systems,[6] and typically remain below 50.[5,6,23] Only a weak positive correlation was observed between switch performance on Nluc and mNeonGreen (**Supplementary Figure 8**). This is unsurprising, given that the coding DNA sequences (CDSs) of genes can significantly impact translation rates, [44] and thus RBS scores. This suggests that the CDS of the attached genes must be considered when designing Base Gap switches."

We have added the following text to the Discussion section:

"In our structure–function study, we found that coupling longer spaces between the T7 promoter and shorter Base Gaps led to increased leakage. This suggests that RNAP may be better able to traverse smaller gaps when given more opportunity to form stable transcription complexes. Elucidating the source of the spontaneous actuation in this system remains an ongoing area of study."

“Based on our results, we can propose a basic design strategy for Base Gap switches. 1) TBSs should have high RBS scores (<1000 should be discarded) and 2) low Gibbs free energy of mRNA hairpin formation. 3) For general targets, a short (five nucleotide) spacer should be included between the promoter and the TBS. 4) For SNV detection, no spacer should be employed, with the mismatch placed directly after the nick in the +2 position.”

2. Reduced Efficiency with dsDNA Targets

In Figure 2D, dsDNA triggers elicit lower reporter expression compared to ssDNA triggers. Although the authors frame this as evidence of dsDNA compatibility, the observed lower efficiency is not explained. As many diagnostic targets exist in dsDNA form, reduced sensitivity could limit practical application. Potential factors such as target-binding site accessibility, hybridization kinetics, or sequence stability should be investigated to identify and potentially mitigate this limitation.

This is true, we do not fully understand the relationship between dsDNA sequence and their ability to activate Base Gap switches. In the paper, we offer this hypothesis

“We hypothesise that the TBS sites of Base Gap switches can engage in strand displacement with dsDNA targets, thus facilitating hybridisation with their cognate ssDNA.”

Previously, this statement preceded the data. We have now moved it to a discussion after the data, to improve clarity.

note This data has now moved to **Figure 1D**.

As noted (see response to Question 1), we have also performed a structure–function study to help elucidate the mechanisms governing Base Gap switches, though we admit that this does not include dsDNA targets. This is something we are actively working on, though we feel that it goes beyond the scope of this first paper. We plan to include this data in a follow-on study focused specifically on combining DNA amplification with Base Gap switches. We hope the Reviewer can understand our position on this.

3. Clinical Validation of the One-Pot Format

For translational relevance, the one-pot (amplification + IVTT) approach should be evaluated using actual clinical samples and benchmarked against a standard NAAT. A side-by-side comparison of one-pot versus two-step formats with statistical analysis of diagnostic performance (sensitivity, specificity, LOD) would significantly strengthen the manuscript’s clinical applicability claims.

There appears to have been some confusion here. Our NAAT–IVTT assays are all two step, with the RPA and IVTT assays performed sequentially. While we are currently developing a one-pot, one-step assay (RPA + IVTT), it is not yet ready for publication. Currently, we have included an analysis of clinical vaginal swabs for patients positive for *Trichomonas vaginalis* (TV) (**Figure 3C**), and also a discussion of the LoD of the assay (**Figure 3B**). To improve our

confidence in our result, we have repeated this analysis with a larger number of replicates (n = 6) and performed a Mann-Whitney test to show statistical significance (p < 0.01).

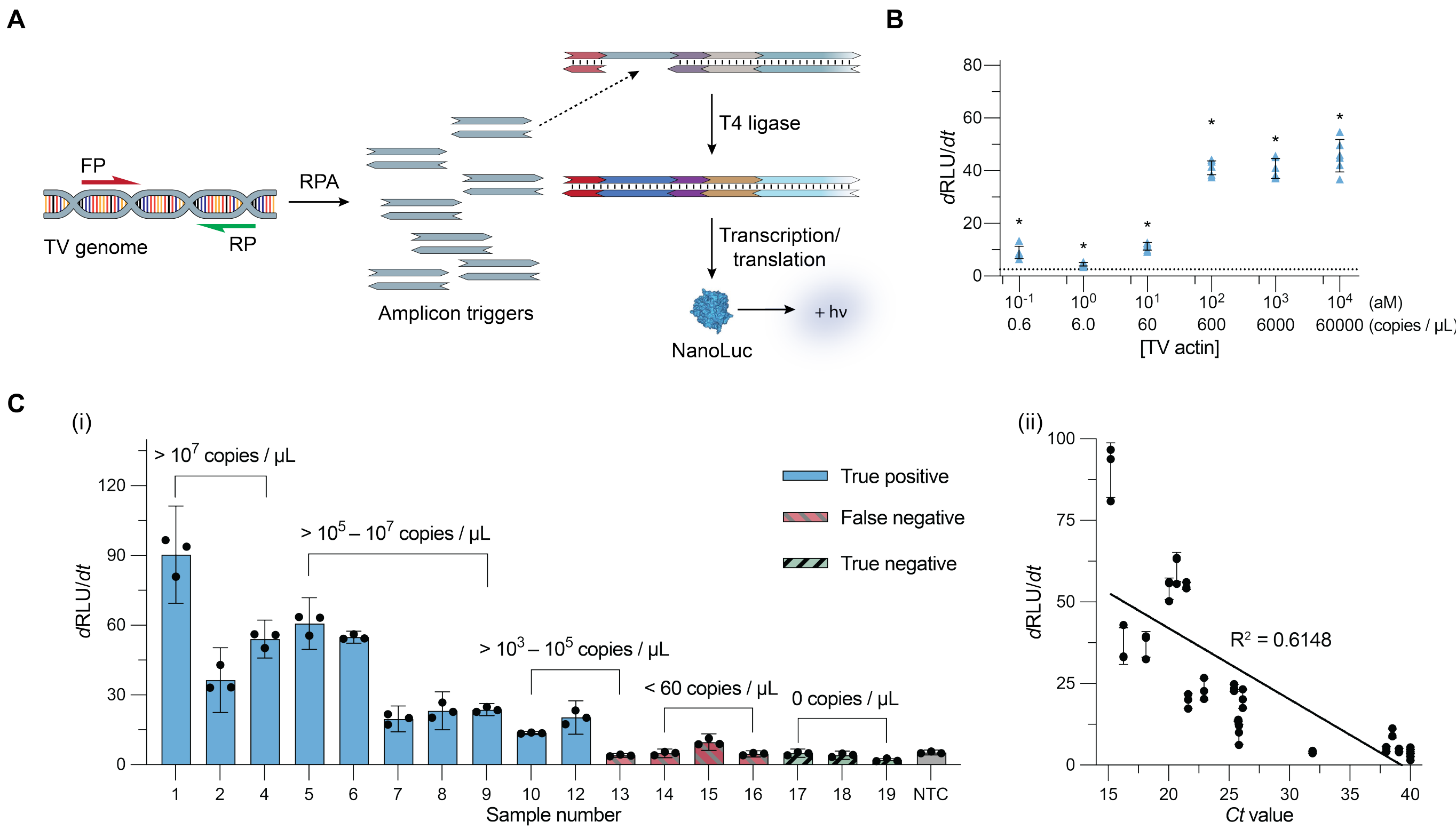

Figure 3. Combining Base Gap switch-controlled SGCs with nucleic acid amplification. A) Schematic representation of the two-step RPA–IVTT assay. Amplicons produced by RPA actuate the Base Gap switch, leading to expression of NanoLuc Luciferase (Nluc). FP and RP refer to the forward and reverse primers, respectively. **B)** Luminescence as a function of TV genome concentration. A positive dose-response relationship is observed, with a limit of detection (LoD) of 0.1 aM (0.6 copies / µL). The LoD is defined as the mean of three negatives (no target) ± three standard deviations. * = $p < 0.01$. **C)** (i) Relative luminescence resulting from RPA–IVTT analysis of patient-derived samples. A comparison with the S-DiaMGTV qPCR assay (ii) showed good correlation. All plots with error bars represent the mean ± standard deviation.

4. Background in Multiplex Detection (Figure 4C)

The CT channel in the NG/CT multiplex assay exhibits elevated background, potentially impacting multi-target assays involving three or more analytes. While the authors attribute this to leakage from the AciI-regulated Base Gap Switch, the specific cause—whether nonspecific activation or another factor—remains undefined. Additional experiments to pinpoint the leakage mechanism and demonstrate an effective mitigation strategy with improved performance are warranted.

This is something we were very intrigued by, though, sadly, were not able to fully unpick. We compared several restriction enzymes (AciI, BsaAI, and HincII) attached to the same Base Gap switch and noted significant leakage in the AciI switch compared to the others.

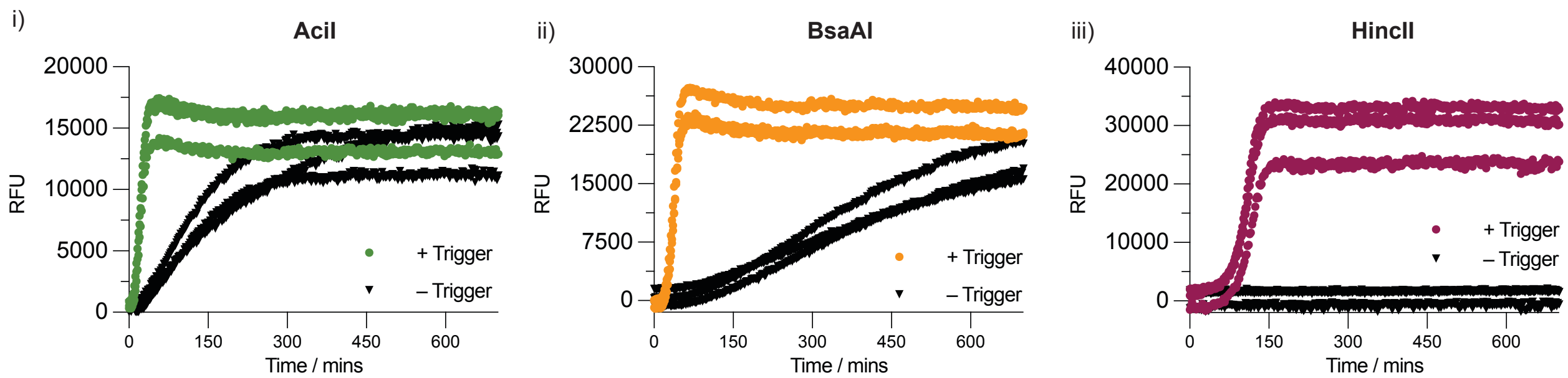

Response figure: Comparison of response curves when the same Base Gap switch is attached to genes encoding for (i) AciI, (ii) BsaAI, and (iii) HincII. Circles represent + Trigger, inverted triangles – Trigger. Despite using the same switch, the response curves differ dramatically.

However, when installing HincII onto the Base Gap switch for CT, we observed no dose-dependent expression of HincII. In fact, we found that spiking RPA amplicons into HincII-expressing IVTT solutions destroyed the activity entirely. This suggests some incompatibility between the components of the TwistAmp RPA kit and HincII. We are actively investigating this, but due to the time constraints, we have opted not to include these results in this publication. This is clearly an area that deserves further, detailed study, and we don't wish to delay publication of this foundational work while we do this. We hope you understand.

5. Specificity Limitations in SNV Detection (Figure 5C)

While the system discriminates A and G variants effectively, C and T variants show partial activation with non-complementary triggers, reducing specificity. The authors speculate on Taq DNA ligase selectivity and transcription efficiency differences but do not provide systematic analysis. For the technology to be broadly applicable to SNV detection, a generalized design framework addressing mismatch position, type, and ligase behavior should be developed and experimentally validated.

This is something we were keen to look into ourselves. We have performed additional experiments to alter the mismatch position, which (in addition to our previous experiments) now paint a more complete picture.

Figure 5B now shows that positioning the nick after the +1 position, with the mismatch directly adjacent in the +2 position, provides the best discrimination. However, this data also shows that mutations to the +3 and +4 positions dramatically decrease expression, suggesting that introducing wobble pairs could be beneficial. **Supplementary Figure 16** further confirms the need to place the mismatch at the ligation junction by showing that mismatches away from this position are well tolerated. **Figure 5C** shows that mutations to C and T residues in the target are best discriminated. **Supplementary Figure 19** confirms that this is likely due to differences in transcription rates when G and A residues are in the +2 position on coding strand of the Base Gap switch. **Supplementary Figure 15**

demonstrates the necessity for a high-fidelity ligase, by showing lower discrimination when a “low” fidelity ligase (T4 ligase) is employed.

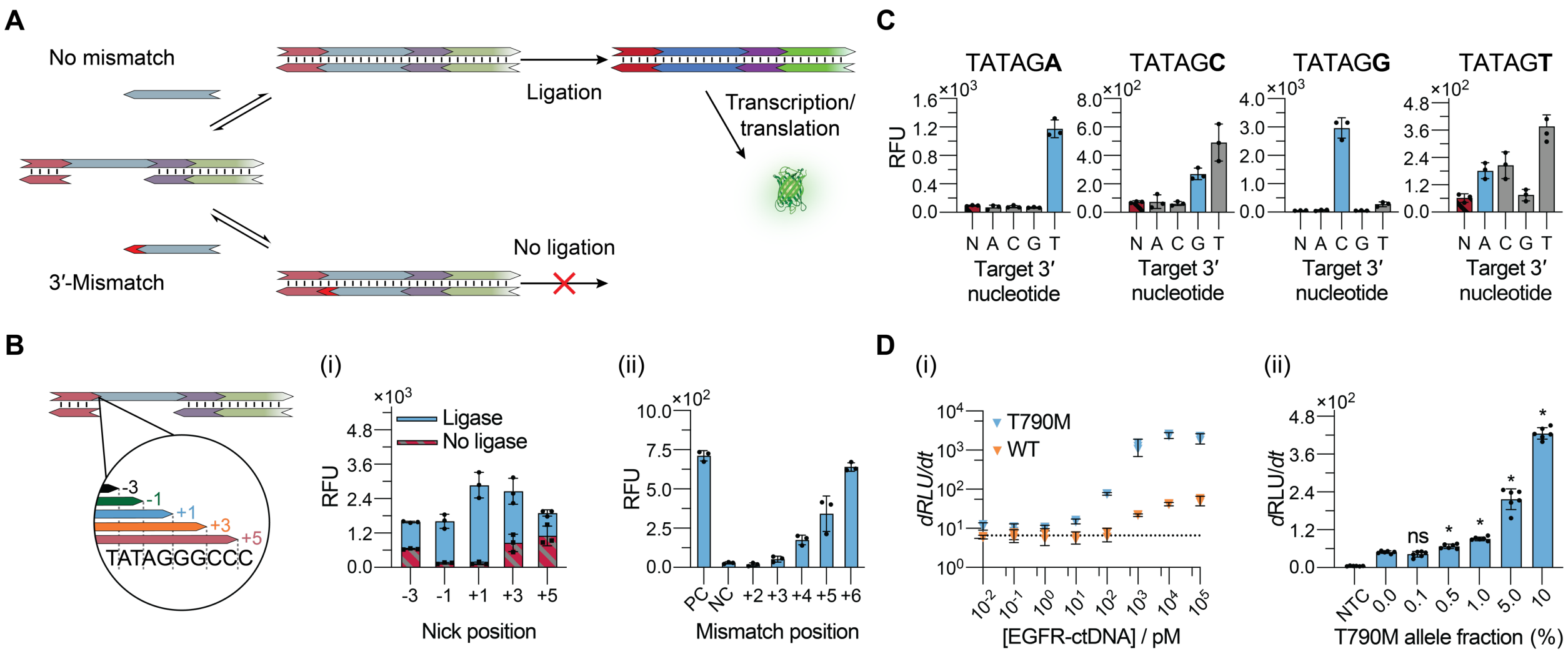

Figure 5. SNV detection using Base Gap switches. A) A schematic describing base mismatch discrimination using Base Gap switches. Mismatches at the 3′ end of the target prevent ligation to the promoter region (red). In the absence of ligation, RNAP cannot bypass the Base Gap and the expression cascade is muted. **B)** (i) mNeonGreen fluorescence as a function of ligation (nick) position, with and without ligase. The positions are numbered relative to the transcriptional start position for the T7 promoter (G,+1). (ii) mNeonGreen fluorescence as a function of mismatch position. A polyguanine (GGGGG) region was introduced directly following the nick site, with triggers containing a T mismatch in the assigned position. Positive control (PC) is a fully matched trigger. Negative control (NC) is no trigger. The greatest ligase dependence was observed with the nick at the +1 position, and high mismatch fidelity was observed with the mismatch placed in the 1-3 position. **C)** Relative mNeonGreen fluorescence values obtained with different base pair mismatches between the switch and the trigger. The bolded letters above indicate the nucleotide at the +2 position of the Base Gap switch. The x-axis group of each graph indicates the nucleotide at the 3′ end of the trigger. *N* = negative (no target). For each graph, the bar representing the complementary base is highlighted in blue. Switches with A and G nucleotides at the +2 position were highly sensitive to base pair mismatches. **D)** (i) NanoLuc Luciferase (Nluc) luminescence as a function of mutant EGFR cfDNA concentration. The Base Gap switch was specific to the T790M (C-T) mutation of EGFR. 10 fM of T790M DNA could be detected above the LoD (dotted line), whereas 1 nM of wildtype DNA was required to achieve significance. (ii) Relative Nluc luminescence as a function of T790M allele fraction (fraction of T790M cfDNA spiked into WT cfDNA). We detected allele fractions as low as 0.5%. All plots with error bars represent the mean ± standard deviation.* = $p < 0.01$, ns = not significant.

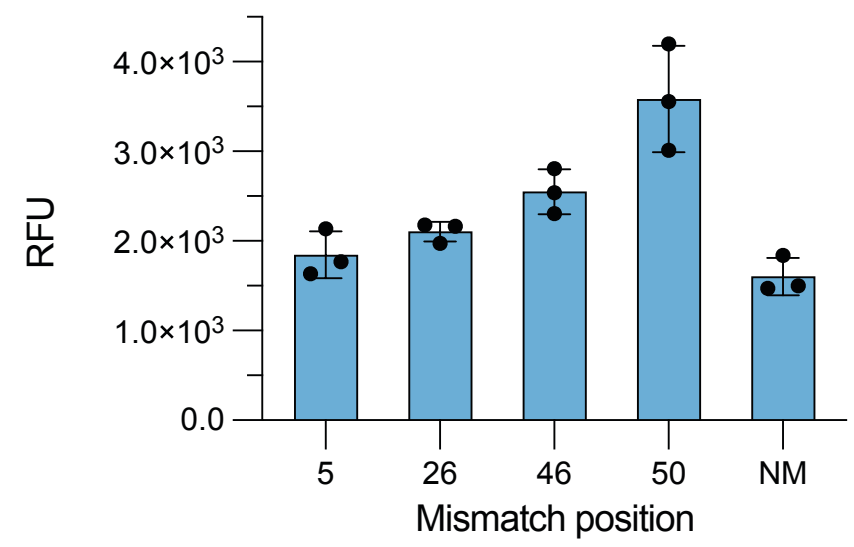

Supplementary Figure 16. Relative mNeonGreen fluorescence values obtained with different base pair mismatches between the switch and the trigger, positioned distal from the ligation junction. NM = no mismatch. Mismatches in the middle of the trigger have no negative impact on expression, and even increase it when placed in certain positions. Error bars represent the mean ± standard deviation

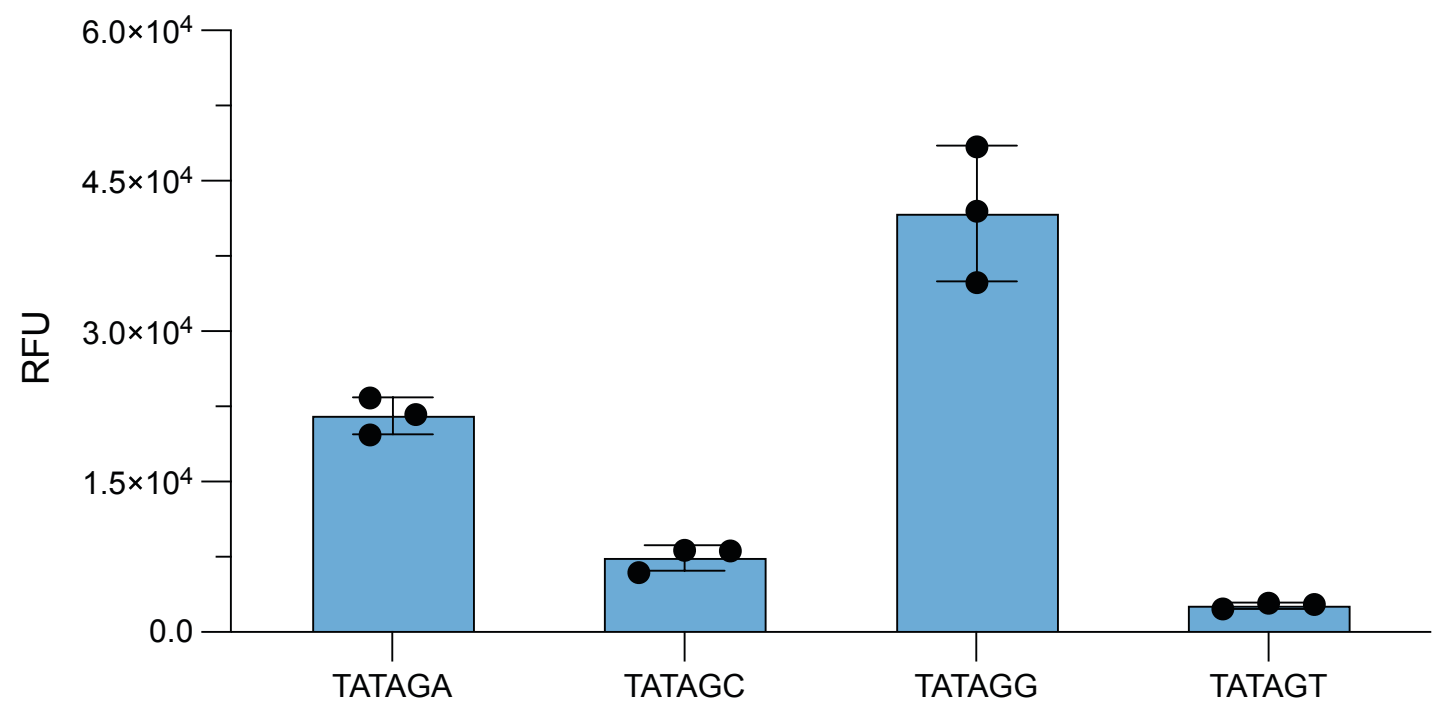

Supplementary Figure 19. Relative mNeonGreen fluorescence as a function of the nucleotide in the +1 position. Expression is greatest with A and G nucleotides in the +1 position. Error bars represent the mean ± one standard deviation.

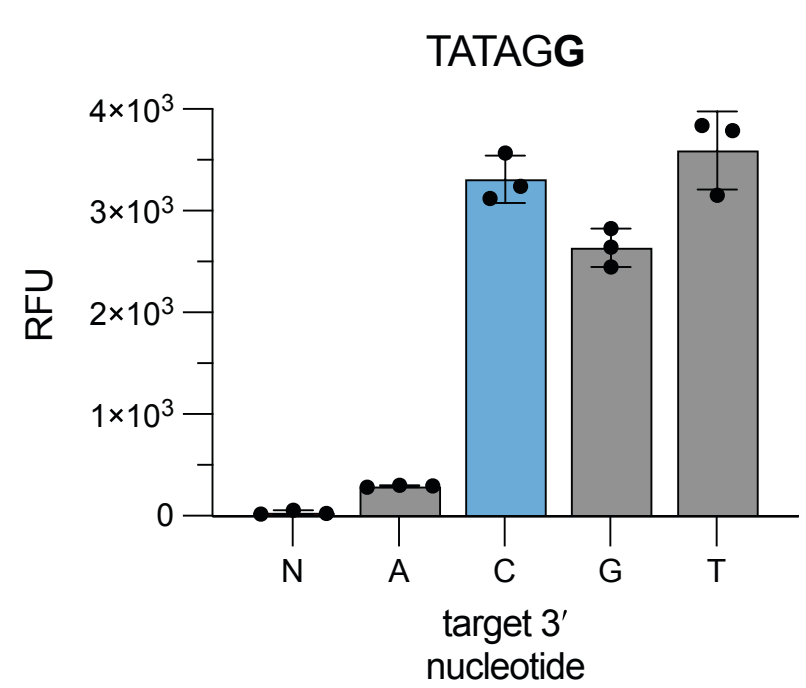

Supplementary Figure 15. Relative mNeonGreen fluorescence values obtained when using T4 ligase to repair nicks between the switch and the trigger, located at the ligation junction. The bolded letters above indicate the nucleotide at the +2 position of the Base Gap switch. The x-axis indicates the nucleotide at the 3′ end of the trigger. *N* = negative (no target). Error bars represent the mean ± standard deviation. The complementary base is highlighted in blue

We have added the following to the Results section:

"To begin, we explored how the position of ligation junction, controlled by the position of the nick on the template strand, impacts expression with and without ligase (**Figure 5B$_i$**). We observed the greatest difference in relative protein expression with and without ligation when the nick was introduced after the +1 position. Furthermore, we observed that the muting capacity of G–T mismatches decreases as they move further from the ligation junction (**Figure 5B$_{ii}$**). Accordingly, optimum mismatch discrimination is obtained with a nick in the +1 position, with the mismatch in the adjacent +2 position."

"Single base pair mismatches at other positions within the TBS did not significantly impact protein expression (**Supplementary Figure 16**). This suggests that selective ligation is important for mismatch discrimination."

We have added the following to the Discussion section:

"An alternative explanation is that transcription rates are slower when guanine and adenosine residues are in the +2 position.[43] Thus, even if ligation were more efficient, the relative expression rates could still be slower than genes containing a mismatch. An experiment in which control genes containing the same sequences but without the nicks supports this theory (**Supplementary Figure 19**)."

*"*Based on our results, we can propose a basic design strategy for Base Gap switches. 1) TBSs should have high RBS scores (<1000 should be discarded) and 2) low Gibbs free energy of mRNA hairpin formation. 3) For general targets, a short (five nucleotide) spacer should be included between the promoter and the TBS. 4) For SNV detection, no spacer should be employed, with the mismatch placed directly after the nick in the +2 position."

6. Trigger Length Effects (Supplementary Figure 4)

The reported trend of decreased performance with shorter triggers is not quantitatively analyzed. Specifically, 3′ truncations abolish activity (presumably due to ligation failure), while 5′ truncations are more tolerated. Without quantitative correlation between trigger length, binding affinity, and expression efficiency—including consideration of melting temperature and mismatch position—these observations cannot serve as a robust design guideline.

To address the concerns regarding design guidelines, we have included a more thorough structure–function study (see our above response to Comment 1). Between **Figure 2** and **Supplementary Figure 7**, we have looked into trigger length, binding affinity (Gibbs free energy of hybridization), melting temperature, and deduced that expression is driven primarily by changes in mRNA after transcription.

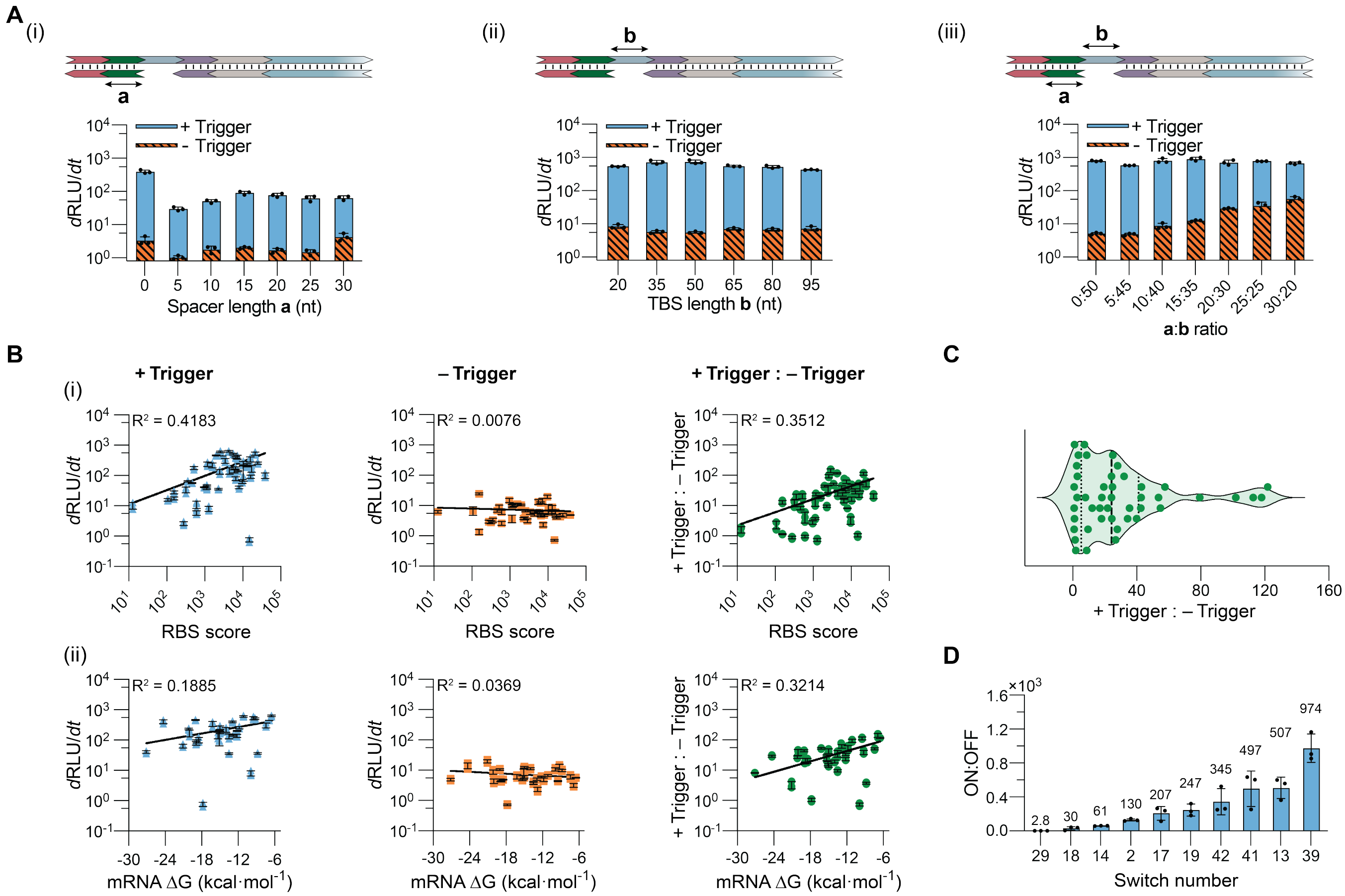

Figure 2. Structure–function relationships. A) NanoLuc Luciferase (Nluc) expression as a function of (i) the number of base pairs between the promoter and the TBS (a), (ii) the TBS length (b), and (iii) the ratio of the the promoter–TBS gap length and the TBS length (a:b) A gap of 5 basepairs is optimal, combined with a high a:b, though expression yields are invariable to TBS length. **B)** Nluc expression from 45 Base Gap-switch controlled SGCs with varying TBS compositions as a function of (i) RBS score and (ii) the Gibbs free energy of formation for mRNA hairpins. Data shows expression in the presence (+ Trigger, blue triangles) and absence (– Trigger, orange squares) of triggers, as well as the ratio of the two (+ Trigger : – Trigger, green circles). Switch performance is positively correlated with increasing RBS scores and decreasing hairpin stability. Switches with an RBS score <1000 (red triangles) were not included in subsequent analyses. **C)** A violin plot of the + Trigger : – Trigger values. **D)** mNeonGreen expression from a selection of switches, from low to high ON:OFF. The numbers above the bars represent the ON:OFF ratio. All plots with error bars represent the mean ± standard deviation. The numbers represent R^2 values were obtained from a semi-log fit of the data.

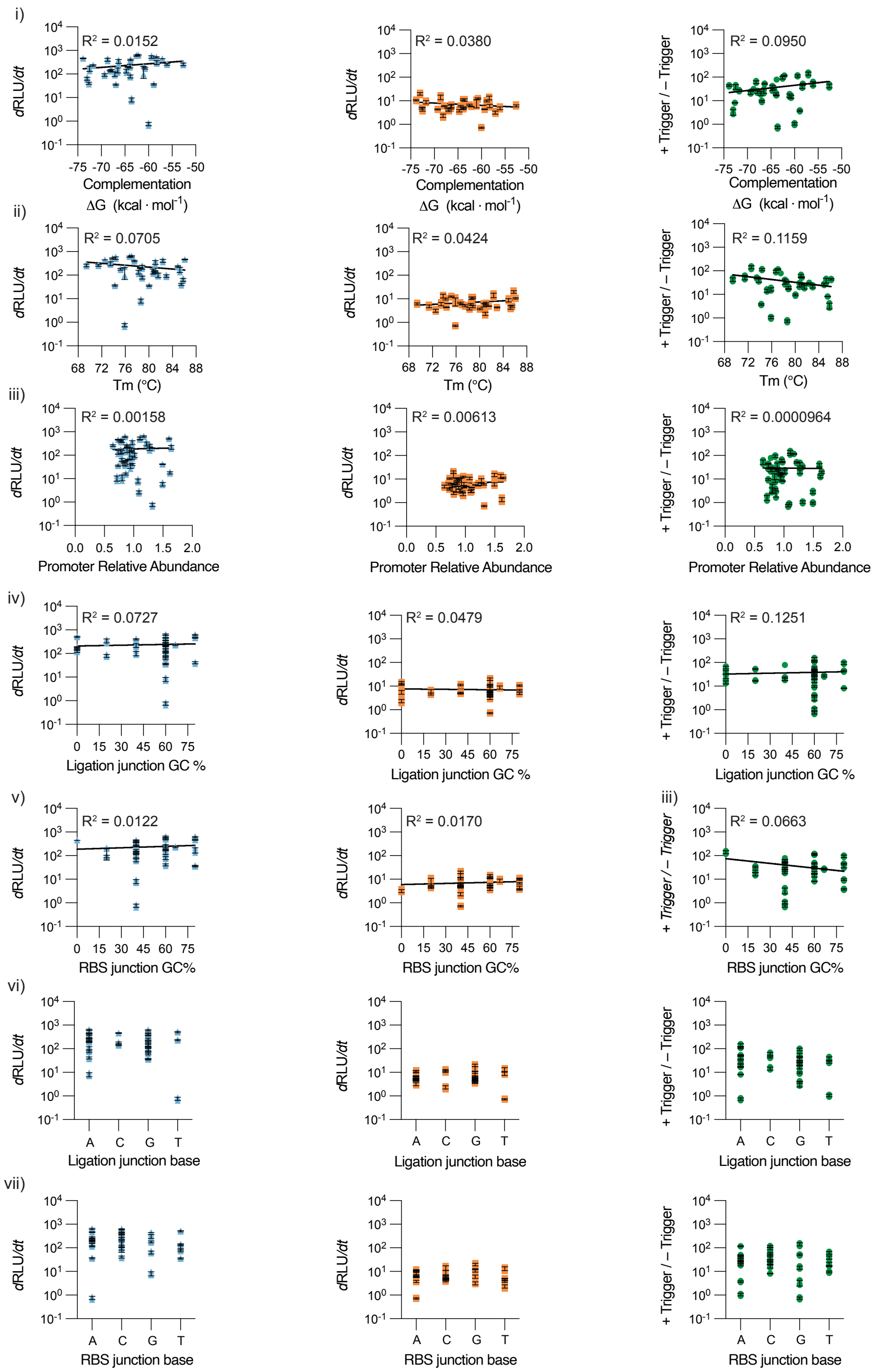

Supplementary Figure 7. NanoLuc Luciferase (Nluc) expression from 45 Base Gap-switch controlled SGCs with varying TBS compositions as a function of (i) the Gibbs free energy of complementation, (ii), the melting temperature (TM) of complementation, (iii) relative promoter strength, as determined by Conrad *et al.*, [1](iv) GC% of the five bases after the ligation junction, (v) GC% of the five bases before the RBS junction, (vi) the nucleotide at the ligation junction, and (vii) the nucleotide at the RBS junction Data shows expression in the presence (+ Trigger, blue triangles) and absence (– Trigger, orange squares) of triggers, as well as the ratio of the two (+ Trigger : – Trigger, green circles). Error bars represent the mean ± one standard deviation.

In **Figure 5** and **Supplementary Figure 16** we investigated the mismatch type and position, determining that mismatches in the +2 position were best discriminated, particularly mismatches with C and T bases.

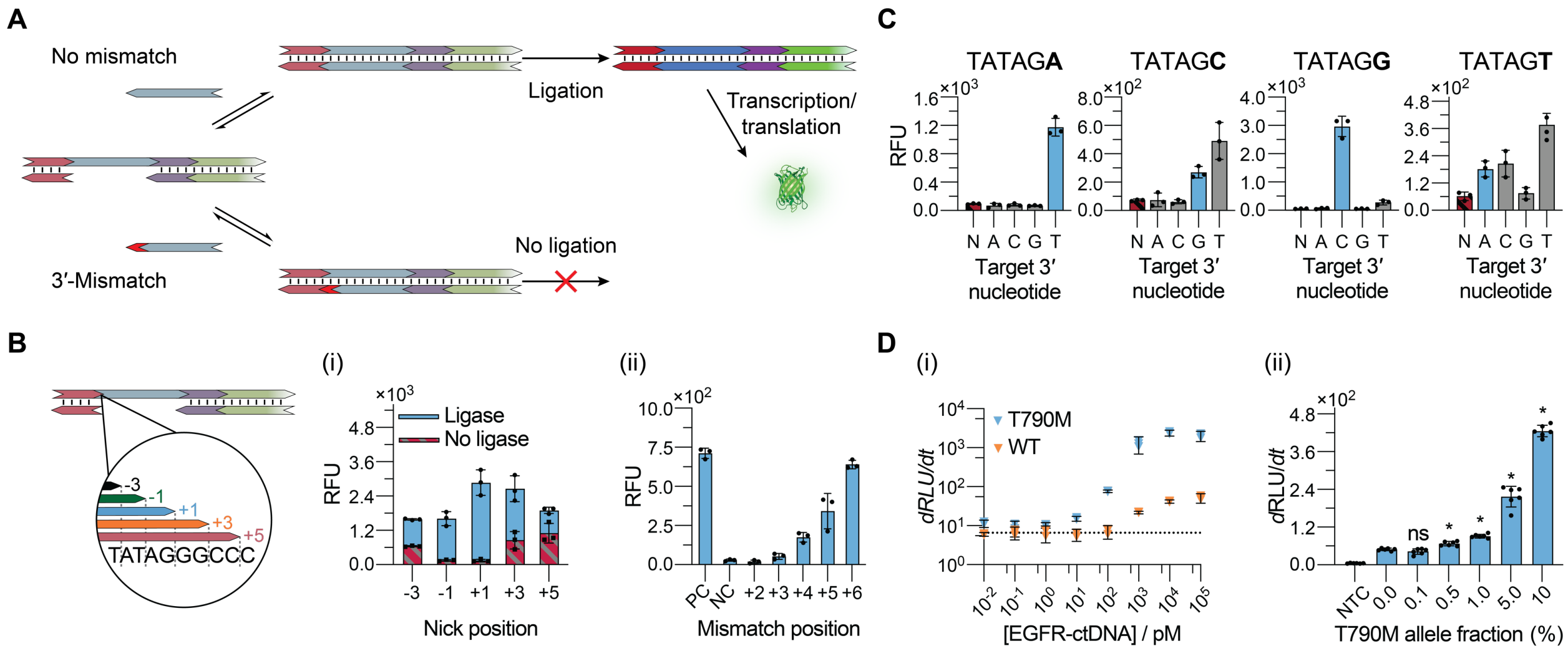

Figure 5. SNV detection using Base Gap switches. A) A schematic describing base mismatch discrimination using Base Gap switches. Mismatches at the 3′ end of the target prevent ligation to the promoter region (red). In the absence of ligation, RNAP cannot bypass the Base Gap and the expression cascade is muted. **B)** (i) mNeonGreen fluorescence as a function of ligation (nick) position, with and without ligase. The positions are numbered relative to the transcriptional start position for the T7 promoter (G,+1). (ii) mNeonGreen fluorescence as a function of mismatch position. A polyguanine (GGGGG) region was introduced directly following the nick site, with triggers containing a T mismatch in the assigned position. Positive control (PC) is a fully matched trigger. Negative control (NC) is no trigger. The greatest ligase dependence was observed with the nick at the +1 position, and high mismatch fidelity was observed with the mismatch placed in the 1-3 position. **C)** Relative mNeonGreen fluorescence values obtained with different base pair mismatches between the switch and the trigger. The bolded letters above indicate the nucleotide at the +2 position of the Base Gap switch. The x-axis group of each graph indicates the nucleotide at the 3′ end of the trigger. *N* = negative (no target). For each graph, the bar representing the complementary base is highlighted in blue. Switches with A and G nucleotides at the +2 position were highly sensitive to base pair mismatches. **D)** (i) NanoLuc Luciferase (Nluc) luminescence as a function of mutant EGFR cfDNA concentration. The Base Gap switch was specific to the T790M (C-T) mutation of EGFR. 10 fM of T790M DNA could be detected above the LoD (dotted line), whereas 1 nM of wildtype DNA was required to achieve significance. (ii) Relative Nluc luminescence as a function of T790M allele fraction (fraction of T790M cfDNA spiked into WT cfDNA). We detected allele fractions as low as 0.5%. All plots with error bars represent the mean ± standard deviation.* = $p < 0.01$, ns = not significant.

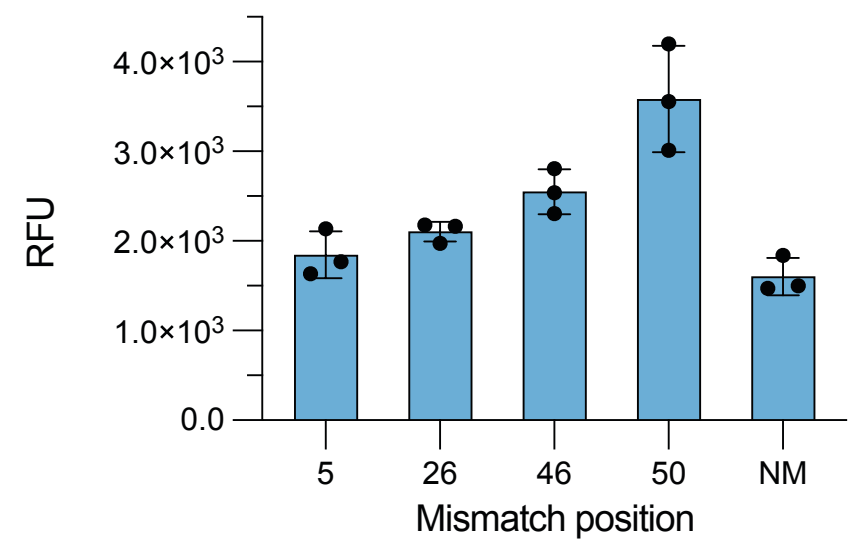

Supplementary Figure 16. Relative mNeonGreen fluorescence values obtained with different base pair mismatches between the switch and the trigger, positioned distal from the ligation junction. NM = no mismatch. Mismatches in the middle of the trigger have no negative impact on expression, and even increase it when placed in certain positions. Error bars represent the mean ± standard deviation

Regarding **Supplementary Figure 6** (Formerly **Supplementary Figure 4**), this data was intended solely to highlight the slight flexibility we have in designing the TBS. These experiments also highlighted the importance of ligation, which helped us to design the Base Gap switches for SNV detection. Given all of the additional structure–function work we have done to determine the guidelines for designing Base Gap switches, we feel like expanding this supporting data would be an unwise use of resources.

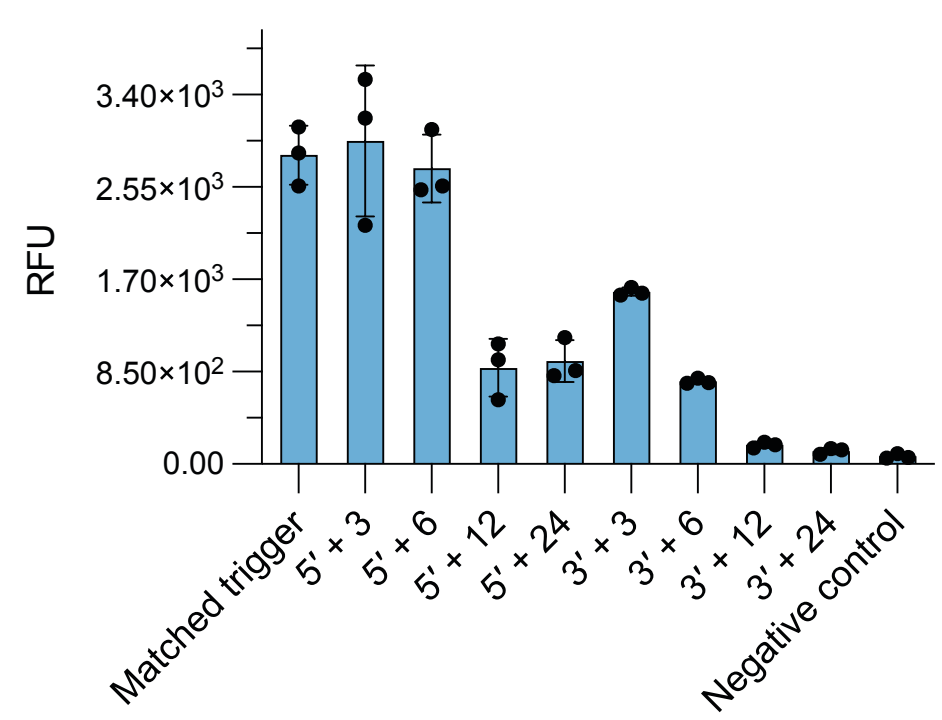

Supplementary Figure 6. Relative mNeonGreen fluorescence as a function of extended trigger length. Extensions at the 5′ end of the trigger are better tolerated, though longer extensions at both termini decrease protein expression. Error bars represent the mean ± one standard deviation

7. Orthogonality Testing in Multiplex Contexts (Supplementary Figure 7)

Orthogonality experiments were conducted in pairwise conditions only, which does not fully replicate competitive environments in true multiplex assays. Additional experiments involving three or more targets are needed to assess possible competition, trigger interference, or false positives in complex mixtures. Quantitative analysis under such conditions would provide greater confidence in the system's multiplexing capability.

This is an excellent point. We have addressed this with **Supplementary Figure 12D**. Here, we incubated a gene under the control of Switch 2 with a mixture of Triggers 1-7, and also Triggers 1, 3–7 (omitting Trigger 2). As controls, we incubating the gene with Trigger 2 alone and with no triggers. Mixing Trigger 2 with a complex mixture of triggers did not decrease

its ability to actuate the switch, and the switch could not be actuated by the complex mixture in the absence of trigger 2. This demonstrates orthogonality even under challenging conditions.

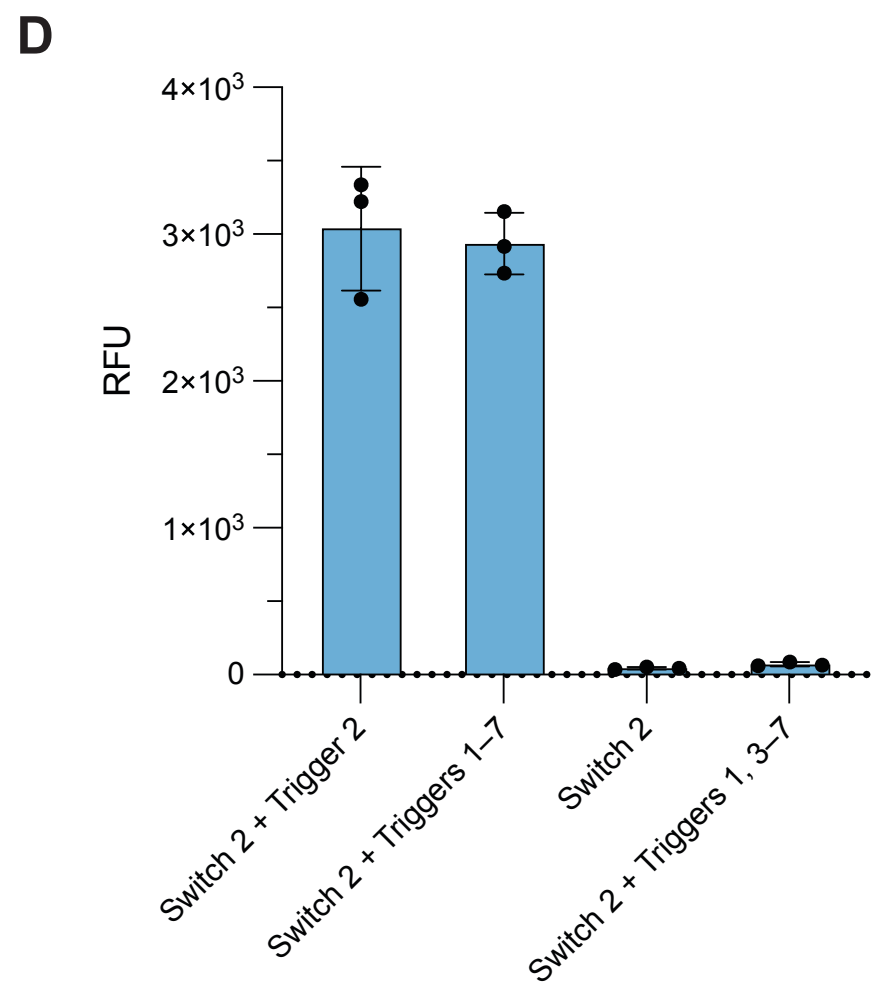

Supplementary Figure 12. Relative mNeonGreen fluorescence from the orthogonal combination of Base Gap switches and triggers. **D)** Relative mNeonGreen fluorescence from a gene controlled by Switch 2, incubated with a mixture of triggers with and without the cognate trigger 2. Expression rates are not impacted by the presence of additional triggers, and trigger 2 is required for switch activation.

This is further evidenced by our experiments with Sirius, mPapaya, and mNeptune (**Figure 4B**). We used mixtures of three circuits and 3 triggers here, and observed no cross-talk (other than the spectral overlap between the red and orange channels, **Supplementary Figure 13**).

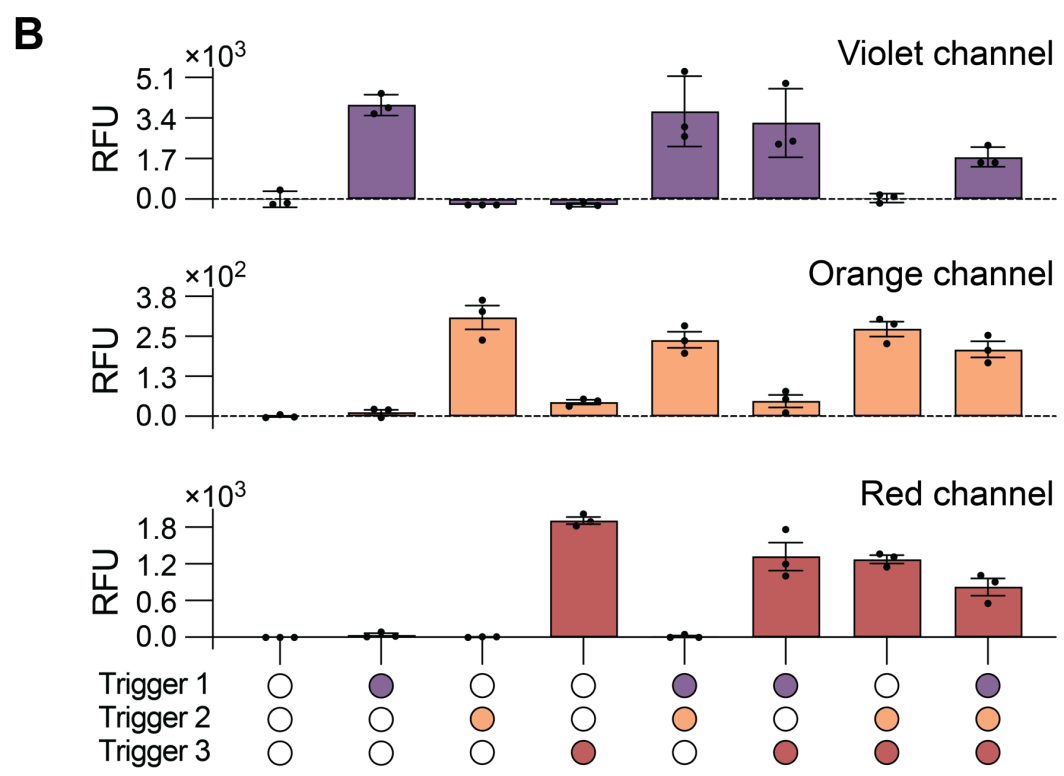

Figure 4. Demonstrating the orthogonality of Base Gap switches. B) Relative Sirius, mPapaya, and mNeptune fluorescence from a mixture of Base Gap-controlled genes actuated by orthogonal triggers. Significant signal is only observed in each channel (violet, orange, red) when the cognate trigger is present.

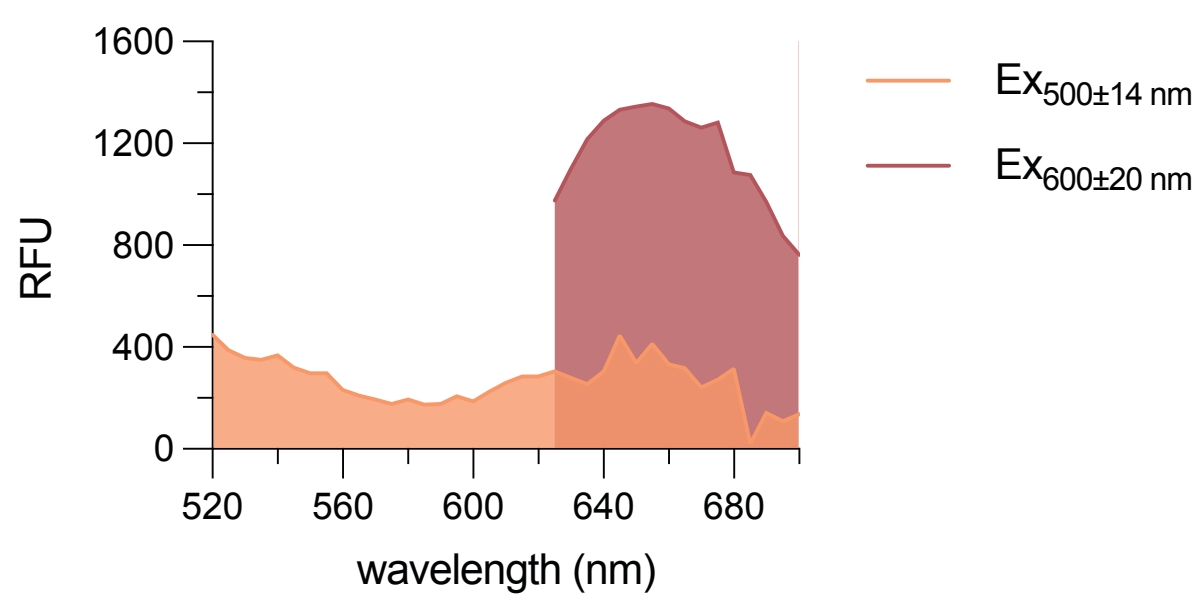

Supplementary Figure 13. Emission spectra for mNeptune obtained at different excitation wavelengths. The data is split into orange (Ex_{500} nm) and red (Ex_{600} nm) channels.

We have added the following to the Results section:

"We observed no decrease in sensitivity or selectivity when incubating a gene under the control of Switch 2 with an equimolar mixture of the other triggers (**Supplementary Figure 12D**). This suggests that Base Gap switches maintain a high degree of orthogonality, even in complex mixtures of targets."

Response to Reviewer 2

First and foremost, we would like to thank you for your incredibly positive comments and suggestions, and for recommending that our work be published. We are particularly pleased that you found the work innovative. We believe that incorporating your suggestions will significantly strengthen the work, so have addressed them as best we can.

General comments

Non-scientific phrases such as "Gratifyingly, orthogonality was maintained" should be removed from the text.

We have gone through the text and removed non-scientific language.

I personally find some of the figures too loaded with information and would suggest moving some of the content of the figures to the SI so that the details can be better seen, but I do not insist on this

Due to the reorganisation of the paper to address the Reviewers' comments, we have now reorganised the data completely. We have moved some data to the SI, going from 11 supplementary figures to 17. We have also redrawn all of the manuscript figures to increase the white space. We believe this improves the clarity.

1. Can the authors suggest why the mNeonGreen expression levels are reduced for dsDNA?

This is a very interesting phenomenon that we do not totally understand. With dsDNA, the complement of the Trigger has the same sequence as the TBS of the Base Gap switch, so it is logical to assume that there will be some competition between the TBS and the cognate complement for the Trigger. We propose that the TBS of the Base Gap switch is able to undergo a strand displacement reaction to replace the complement and bind the Trigger, thus kicking off the expression cascade. This is our current hypothesis, and we are currently performing more experiments to prove or disprove it.

We have added the following text in the Results section to discuss this.

"We hypothesise that the TBS sites of Base Gap switches can engage in strand displacement with dsDNA targets, thus facilitating hybridisation with their cognate ssDNA. Since DNA is primarily double-stranded, this result significantly bolsters the utility of Base Gap switches."

2. What was the rationale for targeting the actin gene of Trichomonas vaginalis and not a gene specific for TV?

We chose the actin gene because of its high genetic stability and high copy number on the genome, which would maximise test sensitivity. We chose the specific region of the actin gene because it has already been validated for specificity to *Trichomonas vaginalis* over related species (T. Crucitti, S. Abdellati, E. Van Dyck, A. Buvé, Molecular typing of the actin gene of Trichomonas vaginalis isolates by PCR–restriction fragment length polymorphism, *Clinical Microbiology and Infection*, 2008, 14, 9, 844-852)

3. Can the authors offer an explanation as to why in the Chlamydia trachomatis and Neisseria gonorrhoeae work leakage from the Base Gap switch was observed and why this would result in a lowering of the expression of AciI?

This is an interesting observation that we have not yet been able to resolve. The problem with the AciI gene circuit was not necessarily the low expression in the presence of the trigger, but rather the high leakage, which led to a poor dynamic range. To dig into this, we designed an experiment in which we attached an identical switch to three different restriction enzymes (BsaAI, HincII, and AciI). We found that the leakage was significantly greater in the AciI, even when the switch was identical (see figure below). This suggests that there may be something in the CDS of AciI that is causing switch leakage.

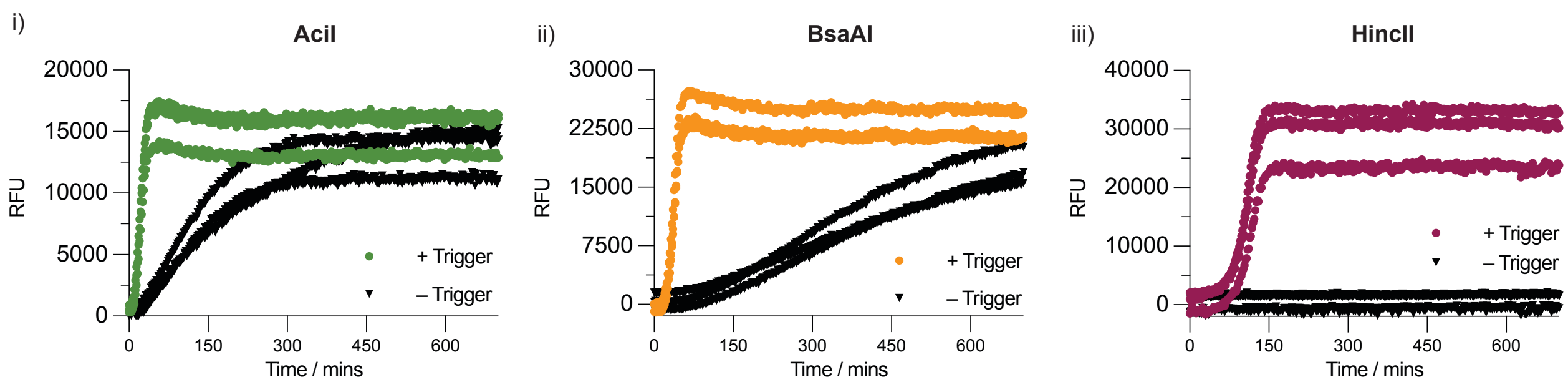

Response figure: Comparison of response curves when the same Base Gap switch is attached to genes encoding for (i) AciI, (ii) BsaAI, and (iii) HincII. Circles represent + Trigger, inverted triangles – Trigger. Despite using the same switch, the response curves differ dramatically.

We also noticed a difference in leakage when identical switches were attached to different genes while studying our switch library on nLuc and mNeonGreen (**Supplementary Figure 8**). This phenomenon has also been noted by others (F. Chizzollini, M Forlin, N. Y. Martin, G. Berloffa, D. Cecchi, S. S. Mansy, *ACS Synth. Biol.* 2017, 6, 4, 638–647). We are actively looking into this, but since the data currently presents an incomplete story, we are opting not to include it here, but rather publish it at a later date.

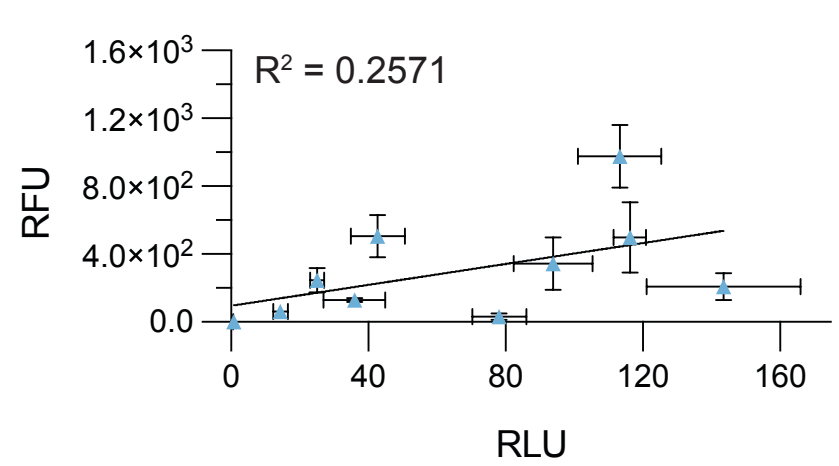

Supplementary Figure 8. Correlation between NanoLuc Luciferase (Nluc) and mNeonGreen expression when the same Base Gap switches are employed. Error bars represent the mean ± one standard deviation.

4. Why were no real samples analysed for Chlamydia trachomatis and Neisseria gonorrhoeae?

We had hoped to do this, but were not able to obtain clinical samples. We hope to publish a more detailed study on this in the future once we have obtained the necessary samples and ethical approvals to work with them.

5. Are the authors confident about the claim "To the best of our knowledge, these results represent the first demonstration of SNV discrimination using a strictly transcription-based biosensor."

We appreciate that this claim may have been a bit broad. While we are confident that this technology represents the first transcription-based expression switch that can discern SNVs, there are other techniques that employ transcription that can also detect SNVs (e.g., SHERLOCK, as pointed out by another Reviewer). Thus, we have opted to remove the statement.

Response to Reviewer 3

We thank you for your generally positive comments and constructive feedback. We particularly appreciate your acknowledging the potential for our work to inspire other sensor designs. We fully appreciate the criticisms of certain aspects of the manuscript, which we have now addressed. By integrating your feedback, we have a much better understanding of how Base Gap switches operate and, as a result, have been able to significantly improve the manuscript.

1a. The template switching reference (Ref. 37) shows a decrease in downstream transcription through a nick site and sequence-dependent template-switching efficiency; however, there is no mention of these effects in the manuscript

We believe our wording in the discussion may have caused some confusion. In the Results, we use the term template strand switching, whereas in the Discussion, we mention RNAP "traversing" the gap. It is likely that these mechanisms are one and the same. Traversing the gap is simply jumping from one template to another, much like it would if there was another template branch. This is even mentioned in Ref 37, where they write "branch and gap jumping", and use the word "traversing" interchangeably with "jumping" (M. Rong, R. K. Durbin, W. T. McAllister, Template Strand Switching by T7 RNA Polymerase, *The Journal of Biological Chemistry*, 1998, 273, 17, 10253 – 10260).

We have added the following statements to the Results section to make this clearer:

"These nicks are not completely deleterious to the function of the switch due to the template-switching ability of RNAP, which allows it to traverse, or "jump", nicks and gaps in the template strand.[39]"

“We attribute this to the aforementioned template-switching,[39] which allows RNAP to “jump” from the mismatched template onto the template encoding for mNeonGreen.”

We have included **Supplementary Figure 3** to demonstrate the impact of the nick on expression compared to a non-nicked control gene.

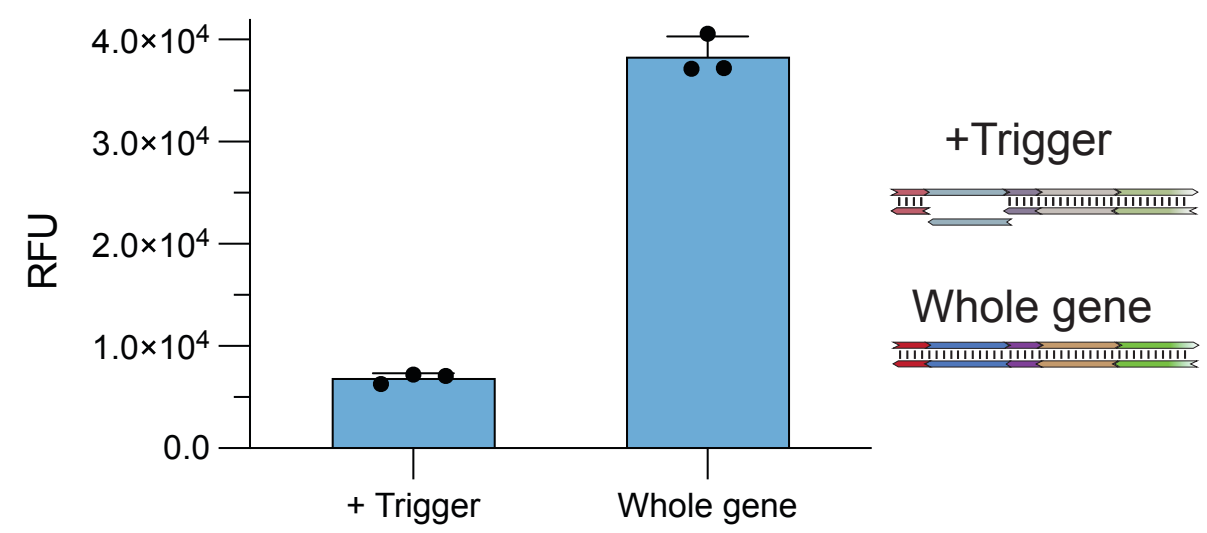

Supplementary Figure 3. Comparison of relative mNeonGreen fluorescence from a gene controlled by a Base Gap switch and an unmodified gene. The nucleotide sequence is identical. Introducing the Base Gap switch decreases expression compared to the control gene. Error bars represent the mean ± one standard deviation.

1b. How did these affect base gap switch performance?

Regarding the sequence-dependent nature of template switching, we have now explored this (**Supplementary Figure 7**) and found that it had no impact on the performance of our Base Gap switches. We studied whether the A,C,G,T content at the termini impacted the performance of our switches, but found no correlation. Indeed, we observed no strong correlation between any parameter and leakage (– Trigger), as would be expected with sequence-dependent "jumping". This was a surprise, and contrasts with the findings of Rong *et al.*, who reported that GC "clamps" upstream of a nick were beneficial to RNAP "jumping", as were poly A strings. We believe we do not observe such effects in our system because the limiting step in IVTT systems is, translation rather than transcription (see the larger comment below for more detail). So, while sequence may be a factor in the rate of transcription from Base Gap, the fact that IVTT systems are rate-limited by translation lowers the impact. This is a very interesting area that we are currently exploring.

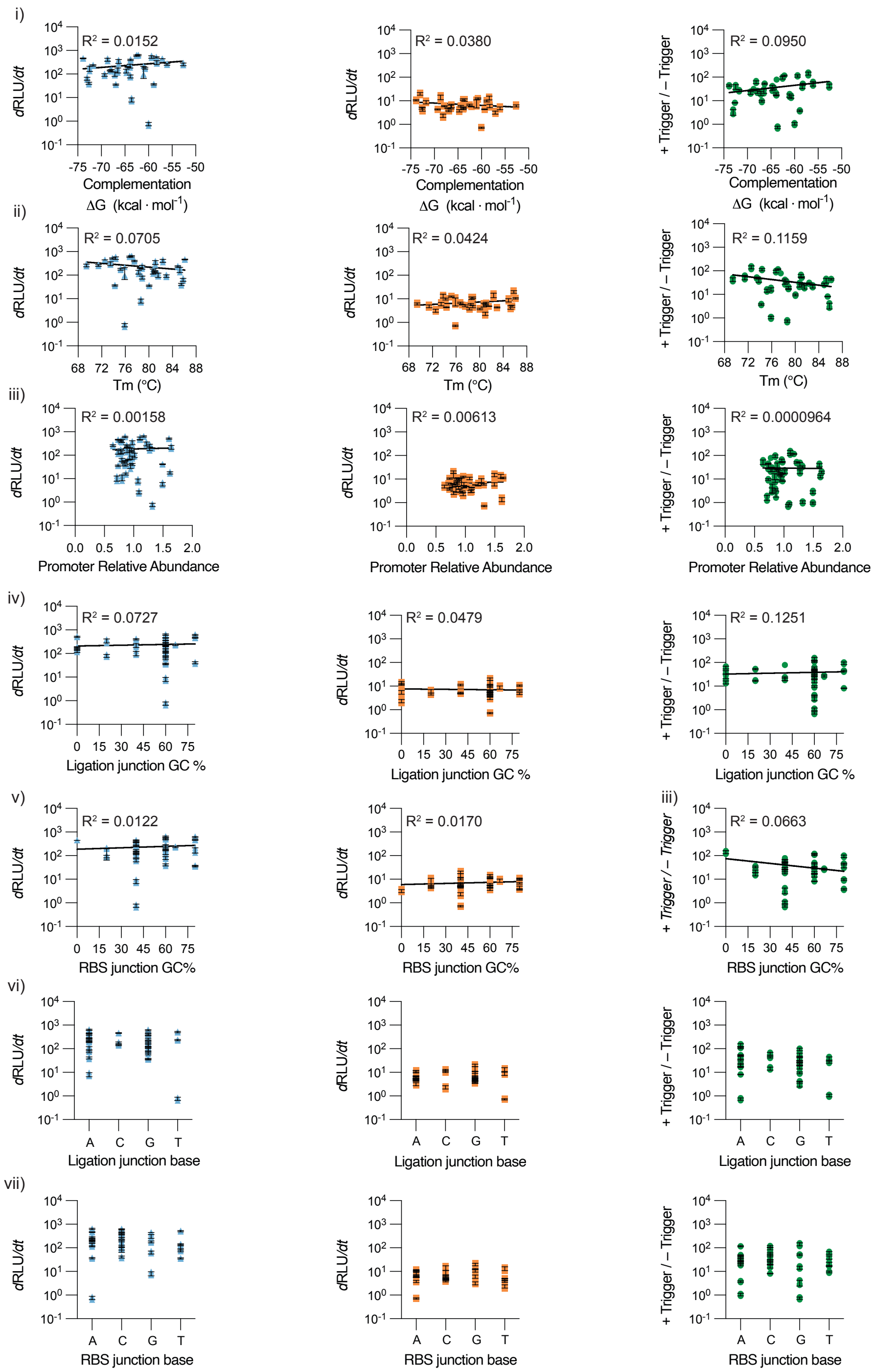

Supplementary Figure 7. NanoLuc Luciferase (Nluc) expression from 45 Base Gap-switch controlled SGCs with varying TBS compositions as a function of (i) the Gibbs free energy of complementation, (ii), the melting temperature (TM) of complementation, (iii) relative promoter strength, as determined by Conrad *et al.*, [1](iv) GC% of the five bases after the ligation junction, (v) GC% of the five bases before the RBS junction, (vi) the nucleotide at the ligation junction, and (vii) the nucleotide at the RBS junction Data shows expression in the presence (+ Trigger, blue triangles) and absence (– Trigger, orange squares) of triggers, as well as the ratio of the two (+ Trigger : – Trigger, green circles). Error bars represent the mean ± one standard deviation

We have added the following text to the Results section:

"The lack of correlation with basepair composition is particularly noteworthy, since prior reports suggest that the template switching ability of RNAP, which enables it to traverse gaps or nicks, is highly sequence-dependent.[39] This suggests that the differences in expression control across Base Gap switches are largely attributable to their relative impacts on translation, not transcription. This concurs with previous findings that translation is the rate-limiting step in IVTT,[40] and has a greater impact on relative protein expression.[42]"

1c. Were particular sequence rules identified for optimal targets?

We discovered this translation-dependence during our structure–function studies (**Figure 2, Supplementary Figure(s) 6 and 7**), which allowed us to determine the optimal sequence rules for Base Gap switches. Stronger mRNA hairpins lower translation efficiency, leading to lower rates of expression. We also found that longer spacers between the T7 promoter and shorter Base Gaps led to increased leakage, suggesting that RNAP is better able to traverse smaller gaps when given more opportunity to form stable transcription complexes. Thus, targets should be chosen based on whether the corresponding Base Gap switch has a strong RBS score and produces a transcript with a relatively weak mRNA hairpin. We will continue to explore this in our lab, as we feel lowering the leakage is key to getting the most from these switches.

We have added the following text to the Results section:

"To inform the rational design of Base Gap switches, we explored how their structure impacts their ability to control expression (**Figure 2**). Given the low levels of leakage observed with Base Gap switch-controlled mNeonGreen genes, we performed these studies using a Nano Luciferase (nLuc) reporter to ensure an observable signal.

"We achieved the best performance with a five basepair spacer, and observed no apparent relationship between spacer length and expression levels. Using this optimised spacer, we varied the TBS length, extending at the 3′ termini to avoid altering the composition near the sensitive promoter–TBS junction (Figure $2A_{ii}$). No significant relationship between TBS length and expression levels was apparent, suggesting Base Gap switches can detect targets of various lengths. Increasing the length of the spacer while decreasing the length of the TBS led to a significant increase in switch leakage (– Trigger) (**Figure $2A_{iii}$**). This suggests that when short TBS lengths are employed, shorter spacers decrease leakage and improve performance."

"To better guide the selection of TBSs, we created a library of 50nt Base Gap switches with varied TBS compositions and monitored their control over nLuc expression (Figure 2B, Figure 2C). We conducted an extensive analysis to correlate the performance of these

switches against multiple structural and thermodynamic parameters (**Supplementary Figure 6**), ultimately finding the best predictors of expression in the presence of trigger (+ Trigger) were RBS Score (Figure 2B$_i$), as calculated using the Salis Lab RBS calculator,[34,35] and the Gibbs free energy of mRNA hairpin formation (**Figure 2B$_{ii}$**)."

We have added the following text to the Discussion section:

"In our structure–function study, we found that coupling longer spaces between the T7 promoter and shorter Base Gaps led to increased leakage. This suggests that RNAP may be better able to traverse smaller gaps when given more opportunity to form stable transcription complexes. Elucidating the source of the spontaneous actuation in this system remains an ongoing area of study."

"Based on our results, we can propose a basic design strategy for Base Gap switches. 1) TBSs should have high RBS scores (<1000 should be discarded) and 2) low Gibbs free energy of mRNA hairpin formation. 3) For general targets, a short (five nucleotide) spacer should be included between the promoter and the TBS. 4) For SNV detection, no spacer should be employed, with the mismatch placed directly after the nick in the +2 position."

2. The paper is almost completely lacking in p-value calculations for statistical significance and the graphs do not show individual data points.

We have now included individual data points for all the graphs, and repeated key experiments with n = 6 replicates to facilitate statistical analysis using Mann-Whitney tests. We can now claim proper statistical significance for the LoD in the assay for *Trichomonas vaginalis* (TV) (**Figure 3B**), and for the SNV variant allele frequency detection (**Figure 5D$_{ii}$**).

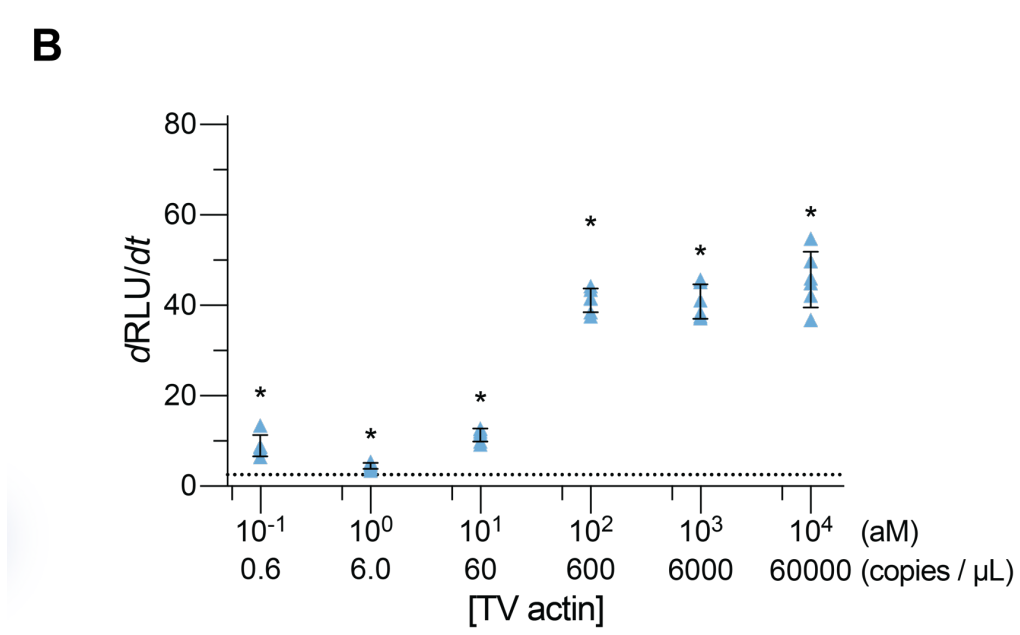

Figure 3. Combining Base Gap switch-controlled SGCs with nucleic acid amplification. B) Luminescence as a function of TV genome concentration. A positive dose-response relationship is observed, with a limit of detection (LoD) of 0.1 aM (0.6 copies / µL). The LoD is defined as the mean of three negatives (no target) ± three standard deviations. * = $p < 0.01$.

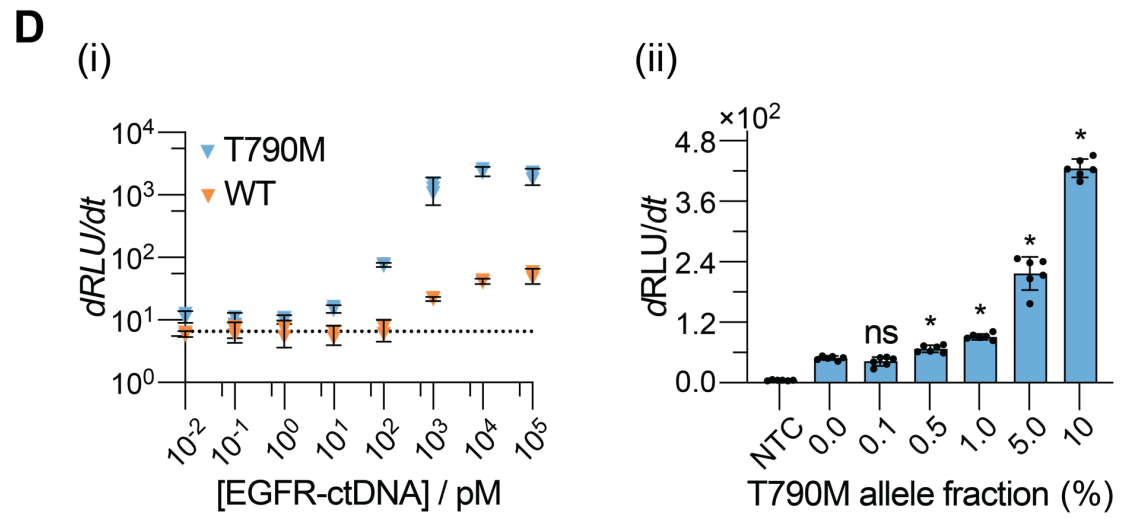

Figure 5. SNV detection using Base Gap switches. D) (i) NanoLuc Luciferase (Nluc) luminescence as a function of mutant EGFR cfDNA concentration. The Base Gap switch was specific to the T790M (C-T) mutation of EGFR. 10 fM of T790M DNA could be detected above the LoD (dotted line), whereas 1 nM of wildtype DNA was required to achieve significance. (ii) Relative Nluc luminescence as a function of T790M allele fraction (fraction of T790M cfDNA spiked into WT cfDNA). We detected allele fractions as low as 0.5%. All plots with error bars represent the mean ± standard deviation.* = p < 0.01, ns = not significant.

We have also added the following text to the Methods section:

"For statistical analyses, Mann-Whitney tests were performed on 6 independent replicates, with * = $p < 0.01$."

3. The ability of base gap switches to detect down to 10 fM of target without pre-amplification is cited as a key feature of the system. However, Figure 5D(i) where this parameter was determined, indicates that the analysis was flawed.

The measurements at 100 fM and possibly 1 pM indicate that the differences between the SNV and the mutant are not statistically significant (plotting individual points would help with this assessment). Consequently, it is unlikely that the measurement at 10 fM is correct and the true limit of detection is 2 to 3 orders of magnitude higher. This LoD analysis should be redone with p-value calculations and plotting of the individual data points.

We apologise for the confusion that has stemmed from this. We would like to clarify that the aim of **Figure 5D$_i$** was not to show a statistical difference between the MT and WT, but rather to show the significance of both from the non-target control (dotted line). Indeed, if you are trying to find significance between the WT and MT curves, this does not occur until perhaps 10 pM. However, the wildtype curve rises much later than the mutant-type, demonstrating the Switch preference for the mutant type. Furthermore, the mean ± 2 standard deviations of the 10 fM reading fell outside of the LoD (2 standard deviations of the mean of the negative), so we called it as significant. We have altered the text of the manuscript to clarify this, included individual points in the graph to make it easier to interpret, and included the individual slopes in the SI (**Supplementary Figure 17**).

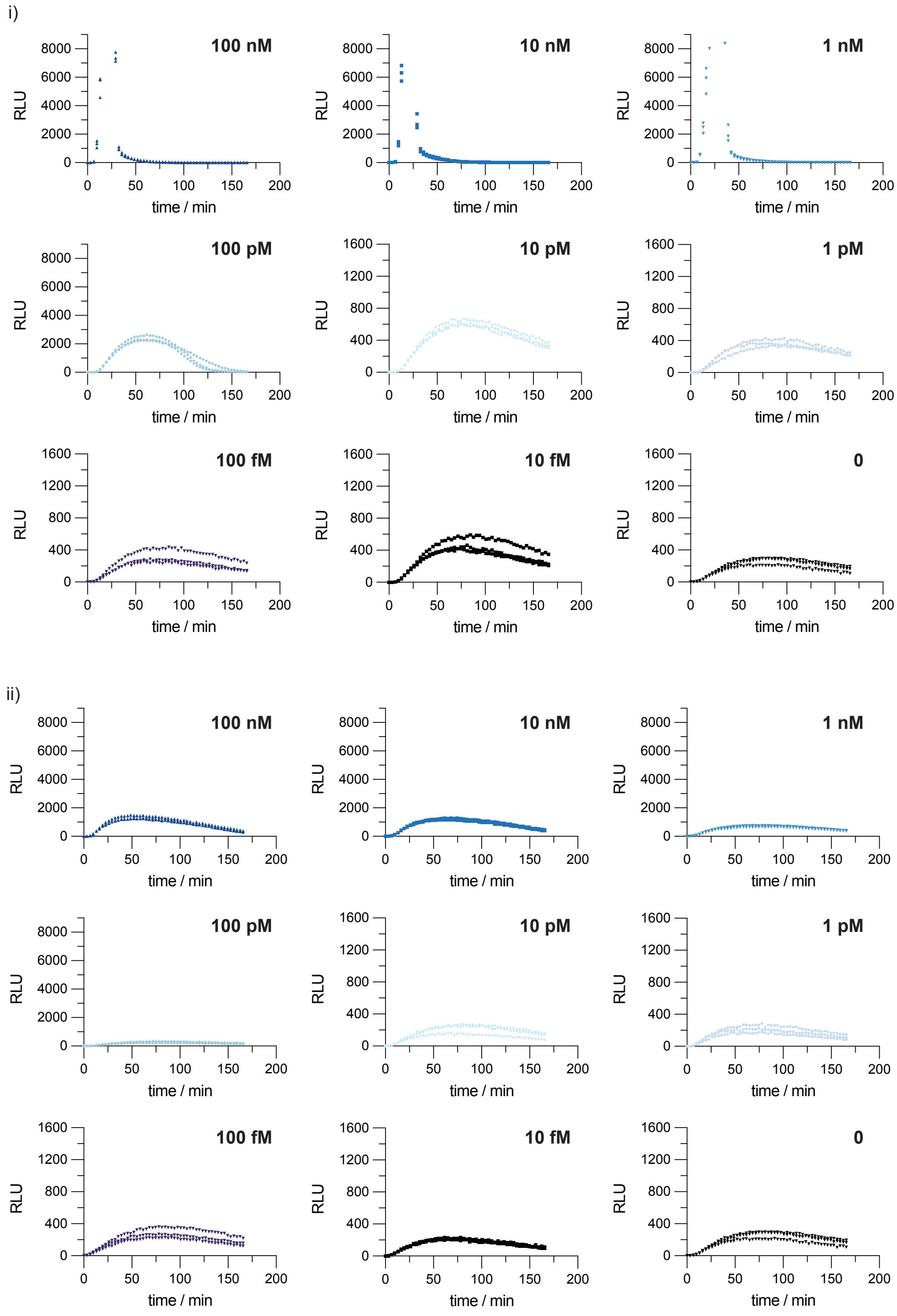

Supplementary Figure 17. Relative NanoLuc Luciferase (Nluc) luminescence as a function of time and (i) EGFR T790M cfDNA concentration or (ii) EGFR wildtype cfDNA. These are the raw curves used to plot the data in **Figure 5Di**. Note the difference in scale from 10 pM to 0.

We have included the following text in the methods section:

"Limits-of-detection (LoD) are reported as the mean of the negative ± two standard deviations."

It is in **Figure 5D$_{ii}$** where we demonstrate the ability of the Base Gap switch to specifically detect the mutant. We have now repeated this analysis with a higher number of replicates and performed a Mann-Whitney test to demonstrate statistical significance. P values are indicated in the caption.

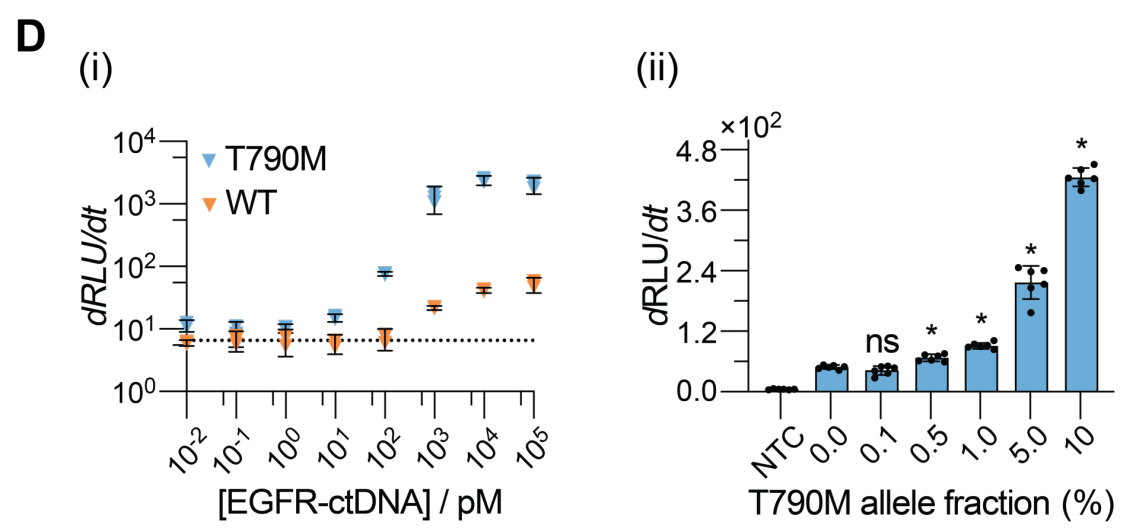

Figure 5. SNV detection using Base Gap switches. D) (i) NanoLuc Luciferase (Nluc) luminescence as a function of mutant EGFR cfDNA concentration. The Base Gap switch was specific to the T790M (C-T) mutation of EGFR. 10 fM of T790M DNA could be detected above the LoD (dotted line), whereas 1 nM of wildtype DNA was required to achieve significance. (ii) Relative Nluc luminescence as a function of T790M allele fraction (fraction of T790M cfDNA spiked into WT cfDNA). We detected allele fractions as low as 0.5%. All plots with error bars represent the mean ± standard deviation.* = $p < 0.01$, ns = not significant.

We have included the following text in the methods section:

"For statistical analyses, Mann-Whitney tests were performed on 6 independent replicates, with * = $p < 0.01$."

4. The manuscript transitions into systems with luciferase outputs and reports a rate of change of the luminescence signal but little detail is given.

4a. What are the units of Maximum dRLU/dT? Why is T capitalized, if it is presumably a time derivative?

We apologise for this typo. Indeed, this is a time-derivative. We have now amended this in all the appropriate plots, and removed the term "Maximum" to improve clarity. We have also included the following text in the Methods section:

"For Nanoluc Luciferase (Nluc) readouts, we employed a slope-based analysis. The first derivative of the raw luminescence curves was calculated, and the point of the maximum slope was used as the readout for expression."

"

4b. At what time is each Maximum dRLU/dT value determined?

We use different time points to determine the dRLU/dt value for different trigger concentrations. Higher concentrations of the triggers lead to steeper slopes at earlier time points. End-point analyses were not possible with Nluc because at high Nluc concentrations (present at high trigger concentrations), the furimazine substrate was converted so rapidly that the maximum signals were often reached within 20 minutes. To combat this, we switched to continuous monitoring and slope-based analysis. The dynamic slope-based analysis was necessary because of the “bell” shaped curves produced by the Nluc irreversible conversion of the furimazine, which is dependent on the concentration of Nluc. Picking a single time-point for every concentration would mean underestimating either the high or the low concentration samples.

4b. Are the Maximum dRLU/dT for each target concentration replicate taken at the same time?

The maximum dRLU/dt values for each replicate were determined at the same time point. While we did perform analyses in which we calculated the dRLU/dt values independently for each sample, we found that the results were almost identical to our original analysis (see below) and statistical significance is maintained regardless. We decided to stick with our original analysis (same time point for each replicate) to avoid any bias.

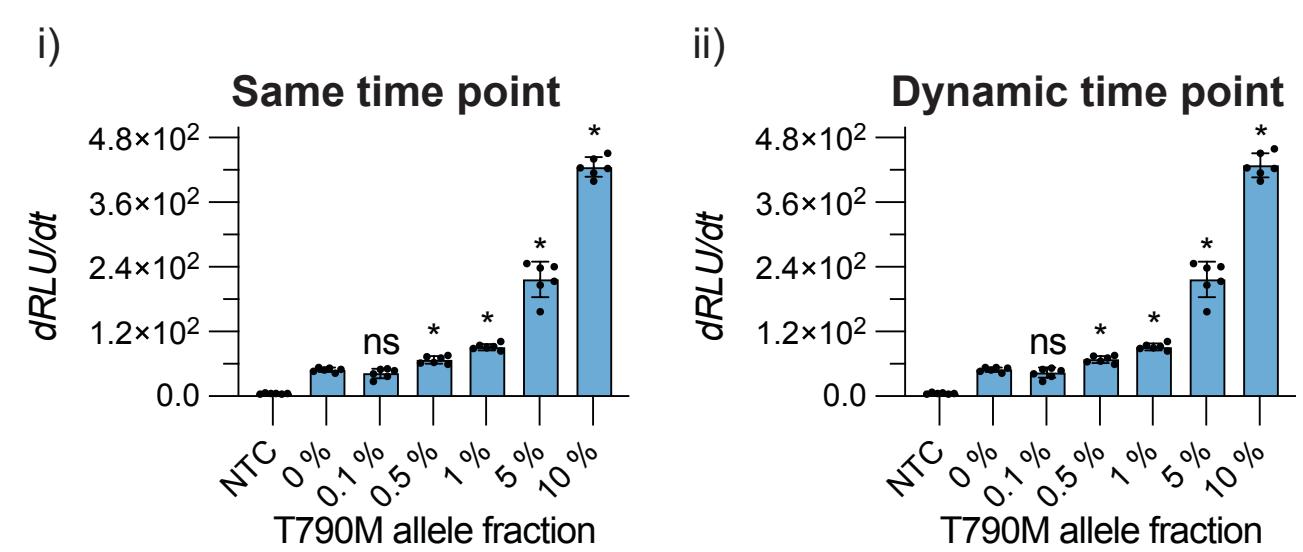

Response figure: Comparison of dRLU/dt at the same time point (i) vs different time points per replicate (ii) for the VAR analysis of EGFR T790M cfDNA.

4c. The RLU vs. time plots should be provided as supplementary information for the limit of detection experiments.

We have included individual RLU vs time plots (**Supplementary Figure(s) 9B, 17, 18**) for each analysis with a LoD.

B

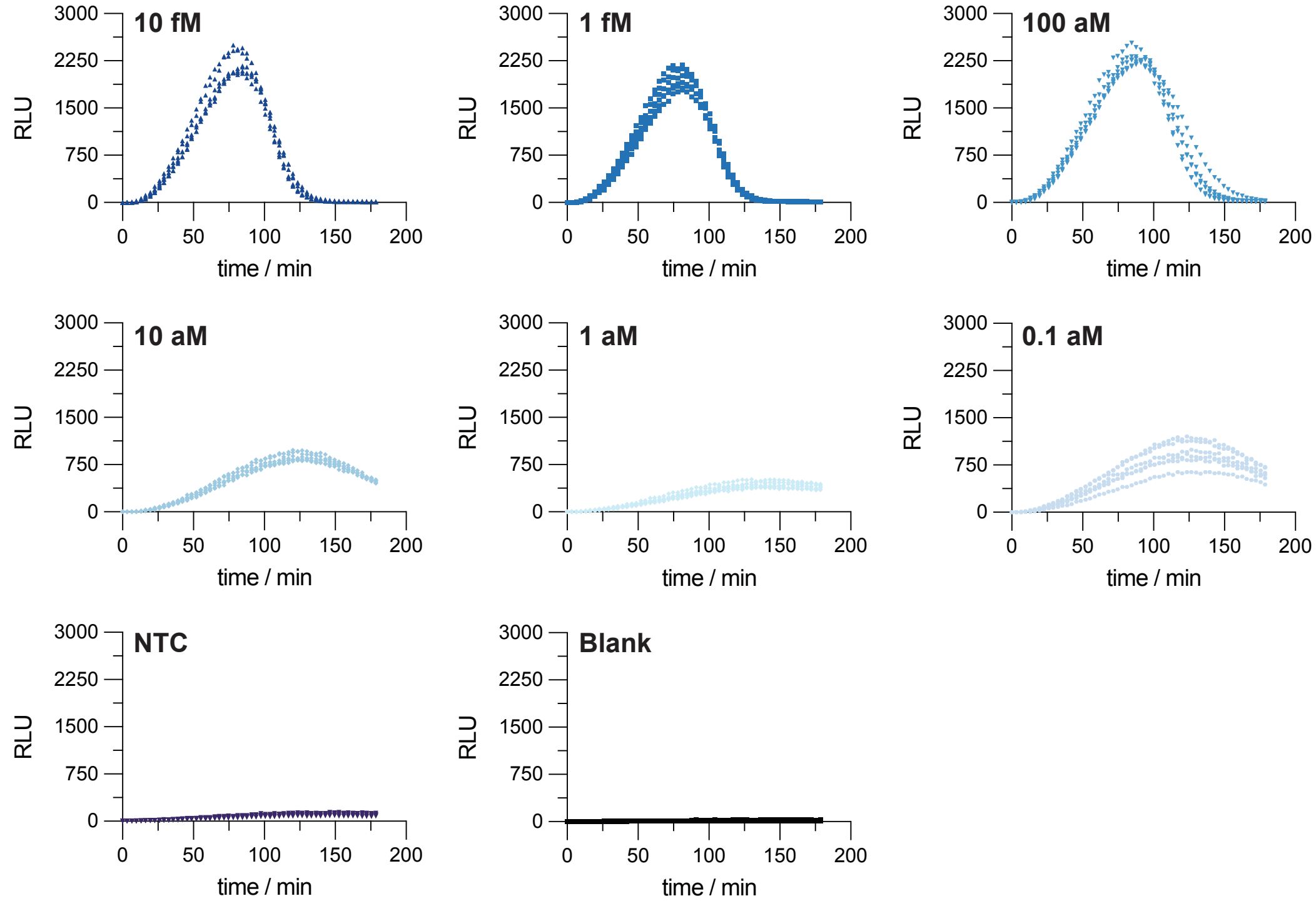

Supplementary Figure 9. (B) Relative NanoLuc Luciferase (Nluc) luminescence as a function of time and genomic DNA concentration. These are the raw curves used to plot the data in **Figure 3B.**

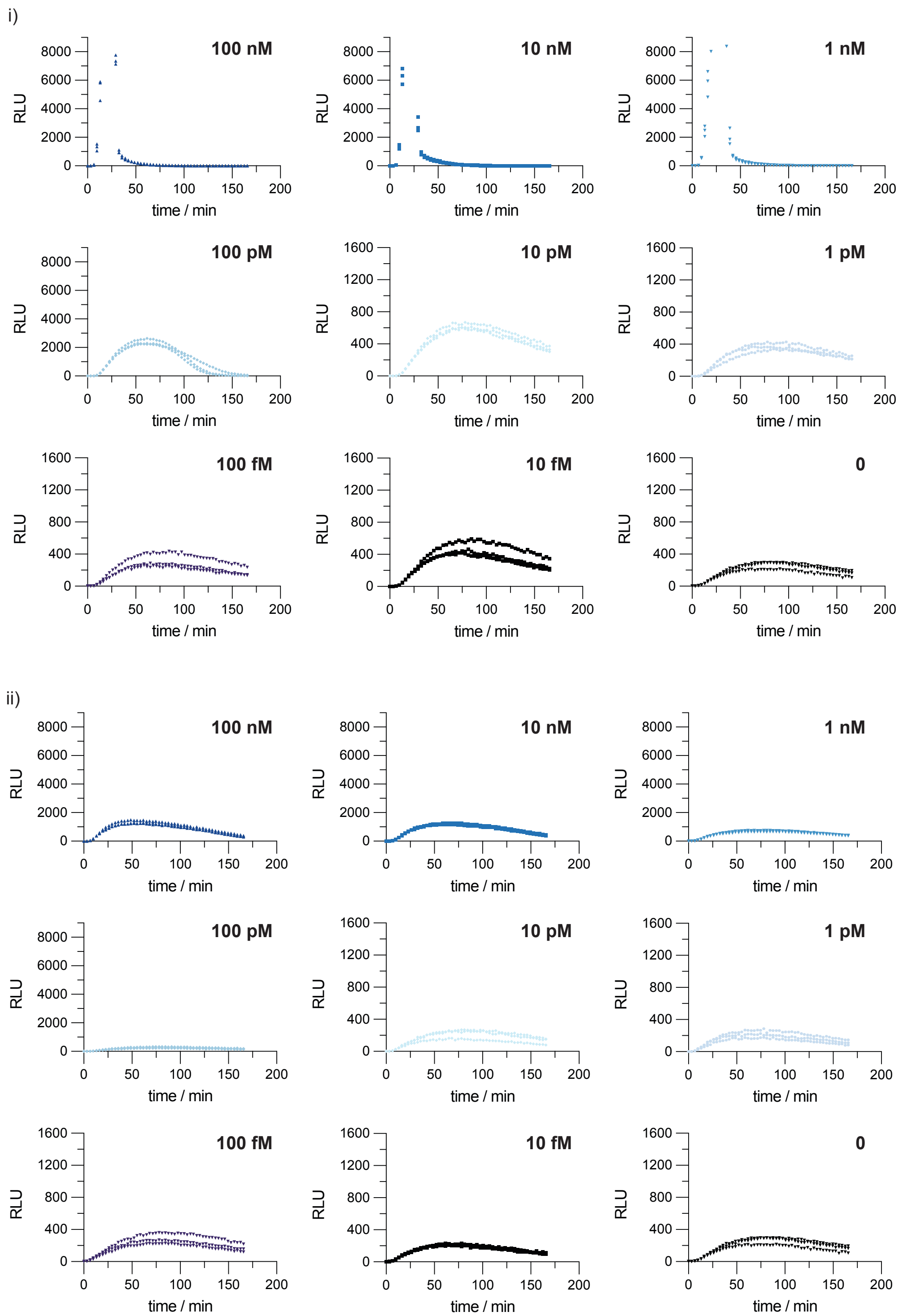

Supplementary Figure 17. Relative NanoLuc Luciferase (Nluc) luminescence as a function of time and (i) EGFR T790M cfDNA concentration or (ii) EGFR wildtype cfDNA. These are the raw curves used to plot the data in **Figure 5Di**. Note the difference in scale from 10 pM to 0.

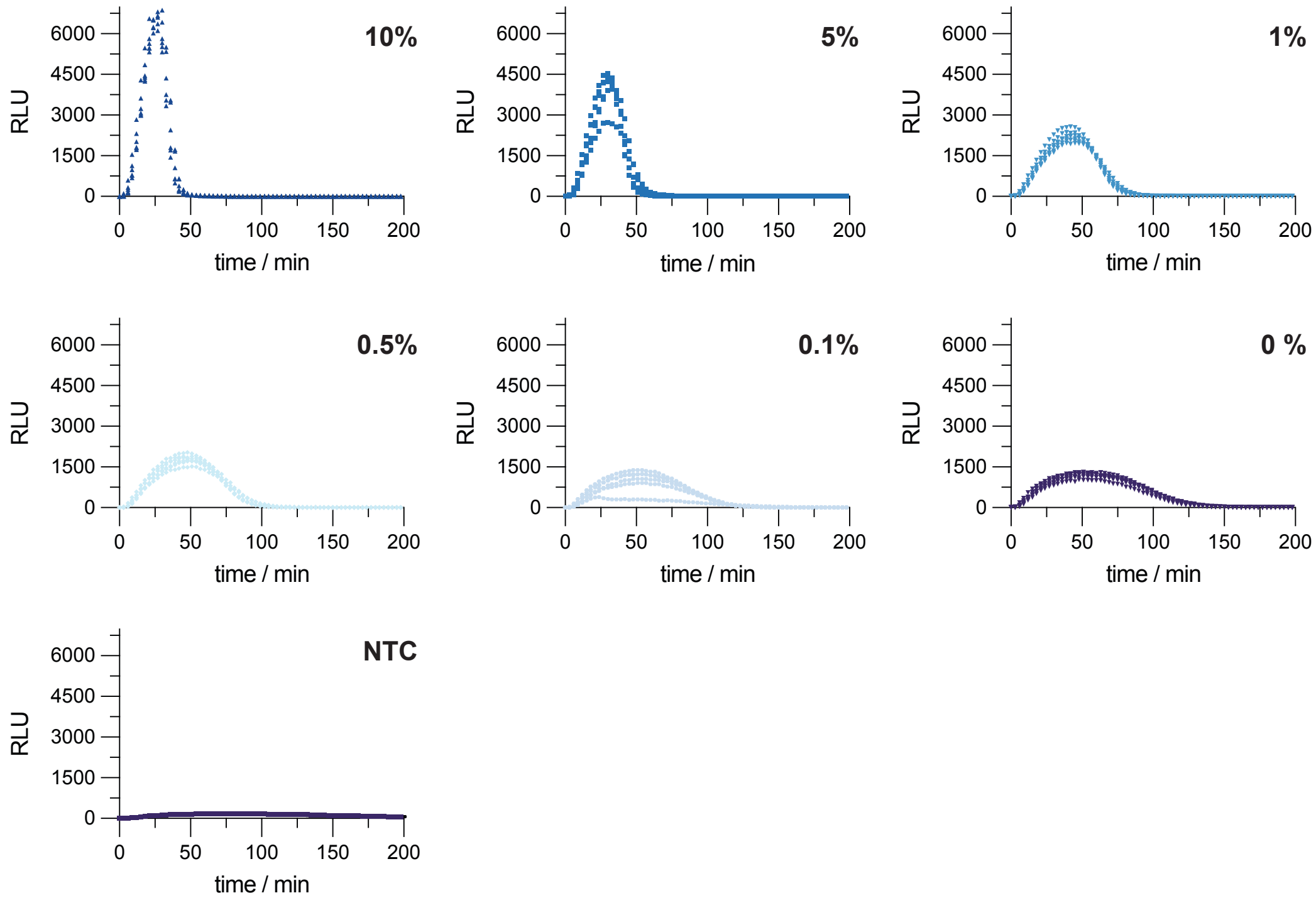

Supplementary Figure 18. Relative NanoLuc Luciferase (Nluc) luminescence as a function of time and EGFR T790M allele fraction. These are the raw curves used to plot the data in **Figure 5Dii**.

5. A major limitation of the system is its combination of transcription-based readout and use of exposed target binding site.

Based on the system design, an off-target sequence that has 5' and 3' ends that match the target sequence with a sufficiently high melting temperature and without a terminator sequence will probably activate the base gap switch. The authors should report in the paper the output of their system using an off-target sequence that has 5' and 3' ends generated from the RPA primers used in their assays, and using the appropriate amplicon strand polarity to bind to the TBS in the switch. This test case is a critical one to include in the paper since isothermal amplification techniques are known to have non-specific amplification. These spurious amplicons will often have 5' and 3' ends generated from the amplification primers, which could thus activate the base gap switches.

This is a good point. We have now investigated this and included two additional supplementary figures. As suggested, we incubated a Base Gap switch with triggers where the termini matched, but the middle section did not (**Supplementary Figure 10B**). While we observed partial activation of the switch in triggers with a high degree of sequence identity at the termini (30 bases at each, or 63% total identity), this partial activation decreased with decreasing sequence identity. In another experiment, we titrated a mixture of primer dimers from the TV actin amplification (generated from a non-template control) against the switch, to determine whether these dimers could activate the switch

(**Supplementary Figure 10A**). While we observed a small increase over the negative control, no dose-dependence was noted. Together, these experiments suggest that the problem of non-specific activation can be entirely mitigated with appropriate primer design.

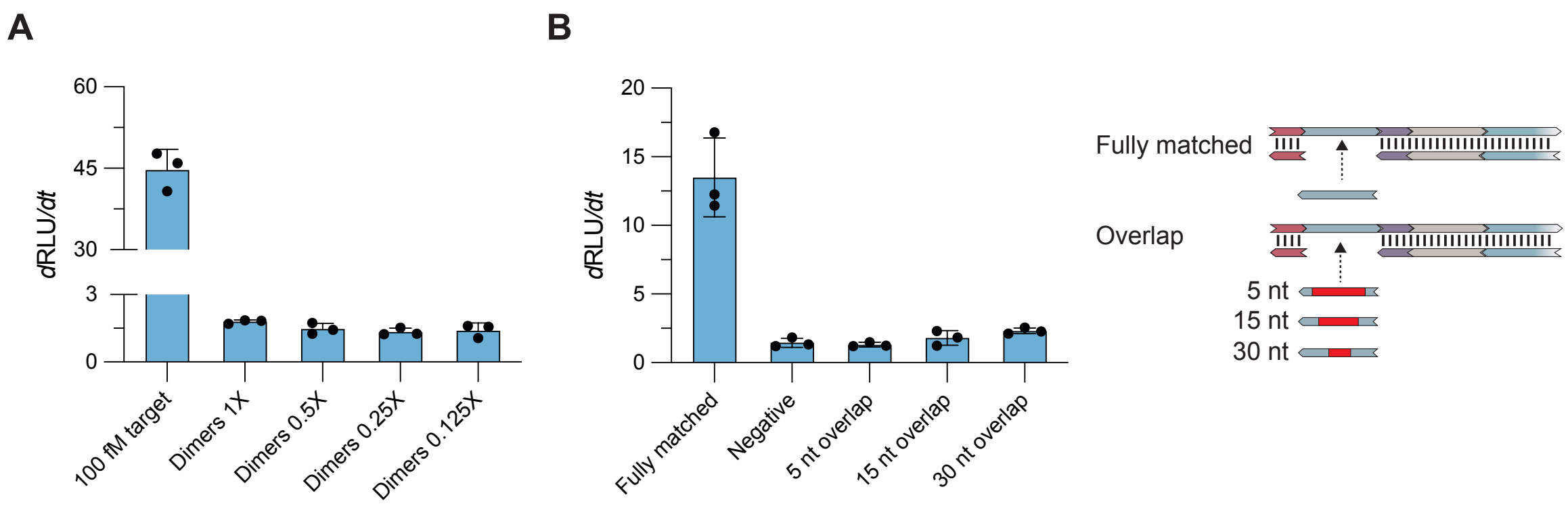

Supplementary Figure 10. NanoLuc Luciferase (Nluc) expression as a function of amplicon dimers. **A)** Dilutions of primer dimers (obtained from RPA in the absence of a target) were incubated with the Nluc circuit with a Base Gap gap switch specific to TV actin. No dose-response was observed. Error bars represent the mean ± one standard deviation. **B)** Relative Nluc luminescence values obtained when only the last 5, 15, or 30 bases at the 5′ and 3′ termini of the trigger (total length = 95 nt) match with the TBS. Significant expression over the negative is only observed with a 30 nt overlap. Error bars represent the mean ± one standard deviation.

We have added the following statement to the Results section:

"No dose-dependent expression was observed when incubating the circuit with primer dimers (**Supplementary Figure 10A**), ruling out non-specific amplification. While non-matched triggers containing complementarity regions at the termini can activate a Base Gap switch (**Supplementary Figure 10B**), this is only observed with a high degree of shared identity and expression rates are significantly reduced. Together, these experiments suggest that non-specific Base Gap switch actuation can be avoided with appropriate primer design."

6. The ability of the base gap switches to detect dsDNA as described by the authors is unexpected.

Complete strand invasion of the single-stranded TBS into a fully double-stranded target would be a very slow reaction without thermodynamic favorability. Have the authors confirmed that the signal is not due to free single-stranded amplicons generated during RPA or PCR?

We initially questioned these results, too. To rule this out, we annealed the dsDNA with a slight excess (1.2x) of the strand that was not complementary to the TBS. Thus, any single-stranded DNA left over from the reaction would be identical to the TBS, not complementary,

and thus not activate the switch. Under these conditions, we still observed significant switch activation, suggesting the activation is not due to free ssDNA.

7. The SNV detection scheme has major limitations that will constrain practical utility.

Since the system requires the SNV to be located near the ligation site, many amplification methods will not be compatible (since the mutation will often be in the primer binding site) and the amplicon will probably have to be treated in some way to ensure the mutation is near the end. The authors should demonstrate how their SNV detection method can be made compatible with a general amplification scheme, or they should at least present a putative application where an amplification-free assay with a putative LoD of 10 pM could be valuable.

This is a fair point. As it stands, the system is limited by the need to place the SNV near the ligation site, which is difficult to reconcile with nucleic acid amplification. This is why we performed our SNV study without any amplification step. We have added the following to the Discussion to address this:

"One notable limitation of our system is the requirement for the SNV to be placed adjacent to the ligation junction. This precludes the use of many nucleic acid amplification techniques, since the SNV would be at the 5′ termini of the primer. Finding ways to couple Base Gap switch-mediated SNV detection with nucleic acid amplification would greatly improve sensitivity; this is something we are actively investigating."

However, despite this limitation, we are confident in the ability of our test to detect EGFR T790M cfDNA in allele fractions as low as 0.5% (**Figure 5D$_{ii}$**). This has clear clinical relevance. The Roche cobas® EGFR Mutation Test v2 requires a VAR of 2-3% to achieve a positive call for T790M (cobas EGFR Mutation Test V2, P120019/S007 Approval Package, Table 4, https://www.accessdata.fda.gov/cdrh_docs/pdf12/p120019s007c.pdf). The cobas EGFR Mutation Test v2 has found extensive use in guiding treatment for cancers related to EGFR mutations (John T, *et al.*, Akamatsu H, Delmonte A ...EGFR mutation analysis for prospective patient selection in AURA3 phase III trial of osimertinib versus platinum-pemetrexed in patients with EGFR T790M-positive advanced non-small-cell lung cancer, *Lung Cancer*, 2018; 126, 133-138, Benlloch S, *et al.,* Clinical Validation of a PCR Assay for the Detection of *EGFR* Mutations in Non–Small-Cell Lung Cancer: Retrospective Testing of Specimens from the EURTAC Trial. PLoS ONE, 2014, 9(2): e89518). Since our test is arguably more sensitive for T790M than an FDA-approved test that has been used to guide treatment, we feel that it already has clinical value. While we agree that more work needs to be done to fully flesh-out the SNV aspect of Base Gap switches, this deserves a thorough study that would go beyond the scope of this current work. We hope that you understand.

8. The authors have at least three novelty statements in the paper that are incorrect:

a) “To the best of our knowledge, these results represent the first demonstration of SNV discrimination using a strictly transcription-based biosensor." However, as a counterexample, Yoon et al., Biosensors and Bioelectronics 208, 114221 (2022) reports a transcription-based sensor with SNV specificity. Furthermore, the category of "transcription-based biosensor" could encompass many other assays that rely on the availability of a functional transcription template. For instance, SHERLOCK assays rely on a transcription template to produce an RNA for the reaction and they also provide SNV specificity.

Thank you for highlighting this. We have now removed this novelty claim.

b) The "first SGC-based assay to achieve an LOD lower than 100 copies without digital discretisation (e.g., CATCH28)." However, in Ref. 53, Figure 1 shows detection down to 1 molecule per µL, which corresponds to an LOD below 100. Ref. 53 is not the only paper that reports SGC-based assays with <100 copies either.

We have also removed this novelty claim.

c) “In our orthogonality test of seven Base Gap switches, we observed an average crosstalk of just 0.89% and a maximum crosstalk of 31.26% (Figure 4A, Supplementary Figure 7). These values drop to just 0.16 and 3.1%, respectively, when we eliminate the combination of CSS-1 and Trigger 3, which was specifically designed with a high degree of paired identity (90%)..... Thus, our data suggest that Base Gap switches may be more orthogonal than traditional toehold switches." The original Green et al. paper describing toehold switches states the following: "For example, the set of 18 switches with less than 2% crosstalk has a reciprocal crosstalk threshold of 50. This metric corresponds to the minimum output dynamic range to expect in a network employing this library of 18 switches." Partington et al. are thus showing a higher maximum crosstalk level of 3.1% vs. 2% using only 6 switches compared to 18 for Green et al. Thus, this novelty claim also does not hold.

We have removed the last sentence of this novelty claim. However, it is worth pointing out that the 2% value reported by Green et al was for a hand-selected subset of their toehold switches, thus is not representative of the toehold switches as a whole. Their full set of 26 switches had a maximum 12% crosstalk.

9. The Methods section describes the base gap switch as being run three times through a DNA NucleoSpin Gel and PCR Clean-up Kit.

9a. How important is this repeated procedure?

This is important. Because we use an excess of the Base Gap switch to be added, repeated purifications are important to ensure it is all removed.

9b. Did the number of purification times affect the leakage levels of the sensors?

We have not observed any impact of repeated purification on leakage, but insufficient purification may decrease sensitivity because the remaining coding strand provides an alternative complementary hybridisation site for the trigger. This can lower binding to the TBS and trigger-mediated specific activation of the switch.

9c. Were the sensors purified through gel extraction?

In our original manuscript, we had not explored gel extraction. However, to ensure the accuracy of our structure–function study, we took this suggestion on board and used gel purification to ensure each Base Gap switch-controlled gene was free of other components and completely homogenous. This allowed us to determine gene concentrations with the utmost accuracy and ensure that no concentration biases affected our results. We confirmed that the gel extraction method did not impact the function of the genes. However, we can stress that gel purification was not necessary for using Base Gap switches.

We have included the following in the Methods section:

"To ensure complete purity, agarose gel extraction was used to purify the Nluc genes used in the structure–function study (**Figure 2**). Briefly, the unstained bands of a 1% agarose gel were cut out and then purified once using a DNA NucleoSpin™ Gel and PCR Clean-up Kit (Macherey-Nagel)."

9d. What was the yield of the final purified base gap switches?

We obtained yields around 60%, with the losses attributed to the 3-step purification.

10. More information needs to be provided for the stability testing shown in Supplementary Figure 3.

Did the reaction subjected to long-term storage contain the enzymes required for the assay or only the DNA components? Since DNA should be very stable at room temperature over 8 weeks, the stability test is only really noteworthy if it is done with the ligase present.

We did not include the enzymes or proteins required for the assay in this study. We simply wanted to show that even though our Base Gap switches contain regions of ssDNA, meaning they could potentially be sensitive to nuclease degradation. These studies showed that this is not the case.

We are currently exploring whether we can lyophilise the entire reaction mixtures (including all enzymes, PURExpress, etc) whilst maintaining activity. We will do accelerated aging studies as part of this, and hope to report the findings soon.

11. The manuscript should also report the number of copies of target per µL the system can detect.

The current version of the paper lists the total number of target copies detected, but that metric is not very informative without the volume used. Volumes used in the RPA reactions should also be provided since the manufacturer instructions may change.

We have now changed the x-axis to copies / µL from copies in **Figure 3B** and **Figure 4C**.

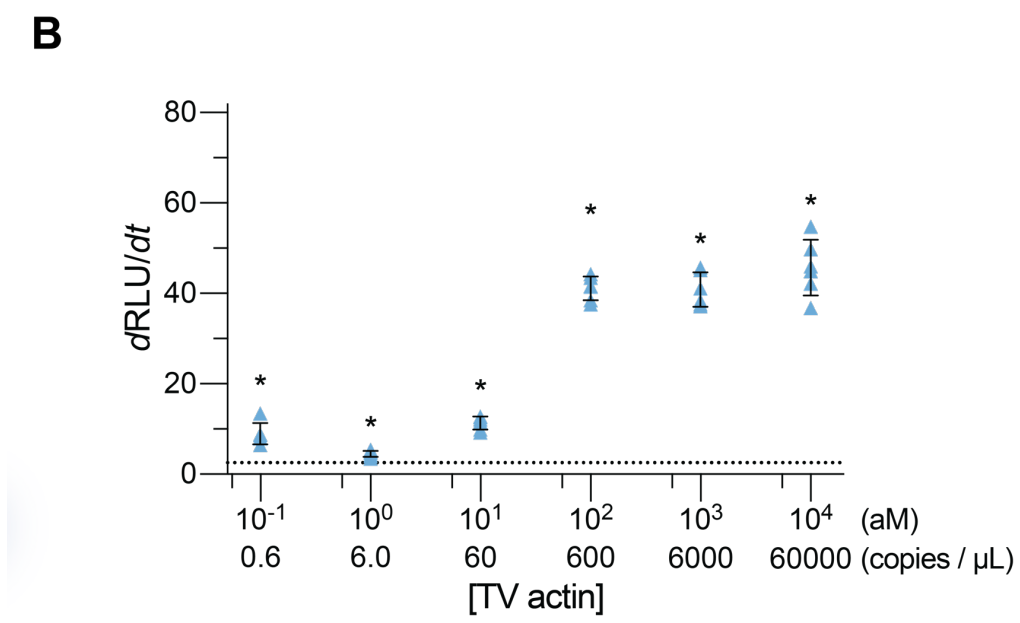

Figure 3. Combining Base Gap switch-controlled SGCs with nucleic acid amplification. B) Luminescence as a function of TV genome concentration. A positive dose-response relationship is observed, with a limit of detection (LoD) of 0.1 aM (0.6 copies / µL). The LoD is defined as the mean of three negatives (no target) ± three standard deviations. * = $p < 0.01$. All plots with error bars represent the mean ± standard deviation.

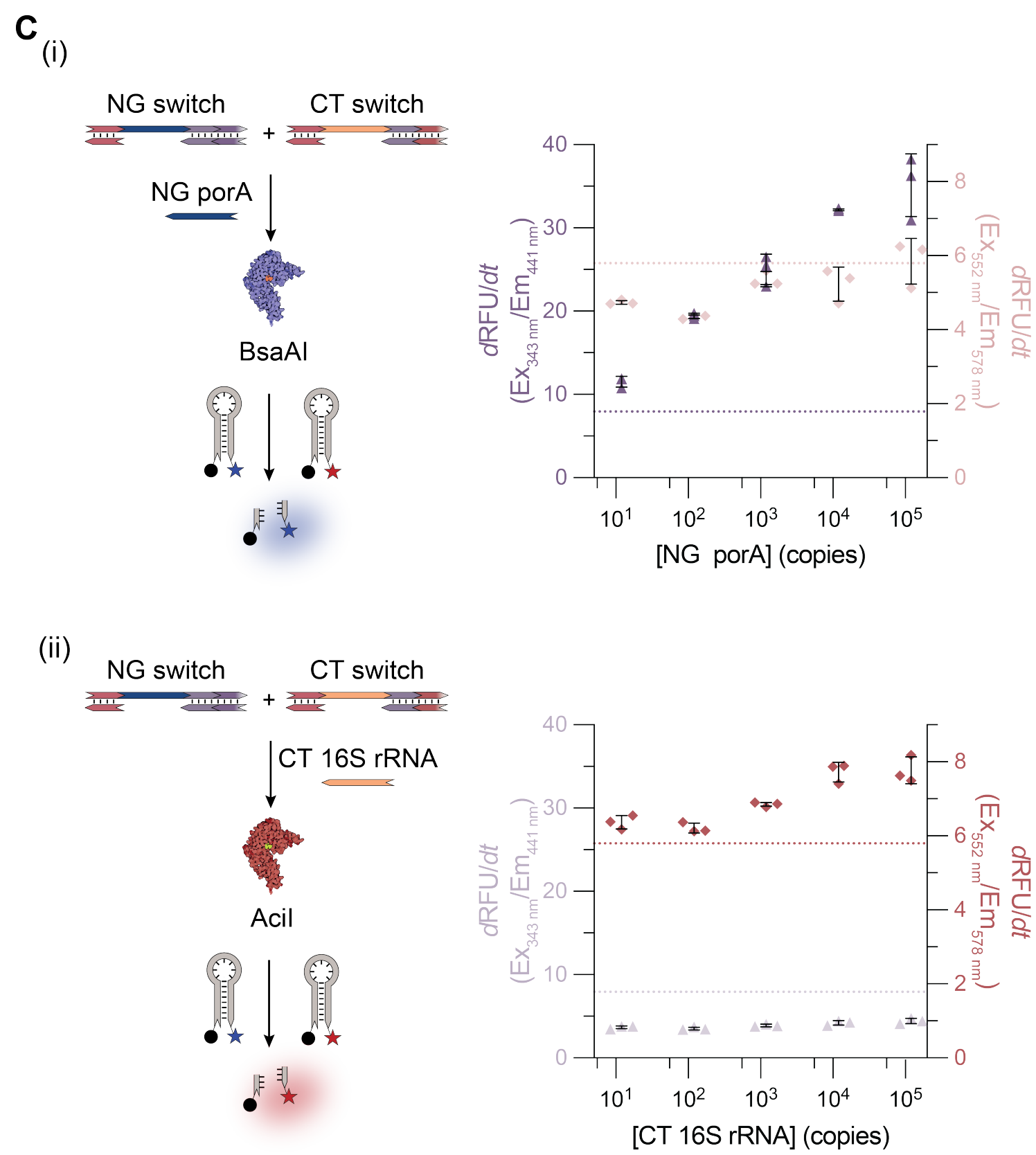

Figure 4. Demonstrating the orthogonality of Base Gap switches. **C)** Relative AciI and BsaAI expression as a function of NG *porA* (i), and CT 16S rRNA (ii) DNA concentration. RPA was performed prior to incubation with the gene circuit. Fluorescence is generated from the cleavage of quenched molecular beacons. Data for each graph were obtained from a mixture of the gene circuits in a single reaction vessel. The violet and red dotted lines represent the LoD for NG *porA* and CT 16S rRNA, respectively, defined as the mean of three negatives (no target) ± two standard deviations. The Base Gap switches maintained their orthogonality, even at high concentrations of the non-target DNA. All plots with error bars represent the mean ± standard deviation.

12. In Figure 1C, it is not possible to see the — trigger bars.

The graph should be updated to make sure these can be seen.

We have now reworked the manuscript, and this Figure is no longer there. However, taking this advise on board, in similar figures we have used a log Y-scale to ensure that low values in -Trigger situations are interpretable.

13. Figure 1 should be updated to make it clear that the region where the trigger binds contains a promoter.

otherwise the mechanism of the system is unclear.

Given the extensive additions to the manuscript, we have now removed the template-strand switches. So, this Figure has now been removed.

14. Line 51: "assa y" should be "assay"

Thanks for pointing this out. We have now corrected this, and thoroughly proof-read the manuscript to try and eliminate similar mistakes.

Response to Reviewer 1

Thank you for reviewing our manuscript a second time, we really appreciate you taking the time. We are pleased that you found our revisions sufficient to recommend publication of our work.

1. Additional references

As the authors have adequately addressed all reviewers' comments, I recommend that this manuscript be accepted after minor revision. The manuscript would benefit from the inclusion of additional relevant and recent references to further strengthen its scientific context and impact. In particular, the authors are encouraged to consider incorporating recent studies in this field, such as those reported in doi.org/10.1016/j.bios.2024.116517, doi.org/10.1016/j.cej.2023.142614, doi.org/10.1016/j.bios.2022.114221, as well as other closely related recent works, to further enhance the discussion and positioning of the work

Since initial submission of this work, several new papers in the field have emerged, as you have highlighted.

The paper you have highlighted by Kim *et al* is cited in our Introduction section:

"Kim *et al.* reported a split T7 RNAP switch that exploits target-dependent sealing of nicks in a T7 promoter to control transcription, and subsequently, translation. [33]"

We have also added the following text to the Introduction section:

"These split T7 switches have also been used effectively in translation-based biosensors in which target presence initiates the transcription of RNA aptamers.[34,35]"

Response to Reviewer 2

Once again, we would like to sincerely thank Reviewer 2 for the kind words and enthusiastic support of our work. We are glad that we were able to address their prior concerns during our revisions and are pleased that they have recommended publication of our work.

Response to Reviewer 3

We thank you again for your constructive feedback and acknowledgement of our efforts to address your concerns. We have taken your current comments on board and address them point-by-point below. We hope that our responses alleviate your concerns.

1. RPA–IVTT assays

More information should be provided on the reactions rather than referring readers to the manufacturer's instructions, which may change or become unavailable. What concentration of the RPA primers was used? What was the volume of the other components? The current TwistAmp Basic Kit does not use 25 µL as the recommended volume. What was the volume of the PUREfrex reactions and the volumes of the different components?

We apologise for this oversight, and have added the following text to the Methods section:

"Recombinase Polymerase Amplification (RPA) of the target (*Trichomonas vaginalis G3 strain genomic DNA* ATCC PRA-98) was performed using the TwistAmp® Basic kit (TwistDX, Maidenhead, UK) according to the manufacturer's instructions. Briefly, for an $n = 6$ titration curve of six samples and one negative (63 samples), forward and reverse primers (76.8 µL), (10 µM in Ultrapure H_2O) were mixed with 944.0 µL of primer dehydration buffer, followed by 262.4 µL of d.d. H_2O and 32 TwistAmp pellets. This MasterMix was split into 21.25 µL aliquots, and to each aliquot the target (2.5 µL, 10 fM – 0.1 aM in d.d. H_2O) was added, followed by MgOAc (1.25 µL, 280 mM). Non-template controls (NTC, d.d H_2O) were prepared similarly. All samples were then incubated at 37 °C for 20 minutes. The samples were subsequently heat-inactivated at 75 °C for 20 minutes and mixed 1:1 with template promoter (14 nM, QuickLigase buffer). The IVTT experiments were performed as described above (*Expression of NanoLuciferase)*; however, PUREfrex® 2.0 (GeneFrontier Corporation, Japan) was replaced as the reconstituted cell-free protein synthesis reagent. Briefly, a PUREfrex MasterMix was prepared (10:1:2 solution 1:solution 2:solution 3), and diluted into an equal volume of d.d. H_2O. For each replicate, a 3 µL aliquot of this MasterMix was prepared and mixed with 1 µL of the deactivated sample. The solutions were covered with mineral oil (5 µL) and incubated in a 1536-well plate at 37 °C, with a total incubation time of 180 minutes and luminescence recorded every three minutes.

2. The section "Trichomonas Vaginalis patient samples" does not provide an acceptable description of the methods used.

"The collection and processing of swab samples are described in the AVEONS study": I have checked the referenced paper and the main text does not describe any methods for PCR sample preparation. How were samples recovered off the swabs? Were the samples subjected to any DNA/RNA extraction prior to use? When were these samples obtained from patients?

We have rectified this with the following additional details in the Methods section:

"The collection and processing of swab samples are described in the AVEONS study.[39] Clinical specimens were obtained from asymptomatic pregnant women in the Democratic

Republic of the Congo.[40] Cervicovaginal lavage samples were collected by rinsing the cervical mucosa with 5 mL of sterile physiological water. Sample collection, handling, and storage procedures have been described previously.[41] Briefly, DNA was extracted from CVL samples using the RNeasy PowerMicrobiome Kit (Qiagen) according to the manufacturer's instructions, with minor modifications (omission of the DNase treatment step). Detection and quantification of *T. vaginalis* DNA were performed using the S-DiaMGTV qPCR kit (Diagenode), following the manufacturer's protocol as previously described.[42] This assay provides quantitative estimates of target DNA concentration (copies/µL), which were used to stratify samples into predefined categories (high, medium, low, and ultra-low target loads). The resulting qPCR data (**Supplementary Table 7**) served as the reference method for subsequent comparison with the Base Gap switch-based assay, for which amplification of the target and protein expression were carried out as described above. ”

3. For the paragraph starting on line 319, were the complementary strands of the dsDNA pre-annealed before adding to the reaction?

In these experiments, we used single-stranded DNA triggers.

We added the following to the Methods section as clarification:

"To better guide the selection of TBSs, we created a library of 50nt Base Gap switches with varied TBS compositions and monitored their control over Nluc expression when activated by ssDNA triggers"

4. The authors should describe exactly what was calculated for the "Gibbs free energy of mRNA hairpin formation".

We have added the following text to the Methods section:

"Gibbs free energies of hairpin formation were calculated using RNAstructure Version 6.5.[38] Briefly, mRNA sequences starting at the +1 position (TATA**<u>G</u>**) and terminating at the end of the target binding site (TBS) region of the switch were folded into hairpins, and the Gibbs free energies provided by the software were used directly."

5. For Figure 3B, the LoD determination should be better explained/justified.

The authors seem to be basing the LoD determination on the separation from the dotted line. The dotted line is probably the mean of the max. dRLU/dt measurement for the 0-copy control experiment (I cannot find a description of it in the caption or main text); however, that control measurement should have its own standard deviation, which is not included in the plot. The Methods section states that "limits-of-detection (LoD) are reported as the mean of the negative ± two standard deviations", but it is unclear which standard deviations are used. The LoD determination must include the variation in the measurements of both the sample and the negative control, but the authors do not appear to be doing that in the paper.

The 6 copies/µL measurement seems to have multiple measurements that fall very close to the 0-copy control line, which suggests that the effective LoD could be higher than 6 copies/µL if the variance in negative control was properly taken into account.

We apologise for the confusion. The dotted line denotes the +2 standard deviation, i.e., the upper end of the variance in measurement. Accordingly, the line already accounts for the variance of the negative. In this plot, we also determined significance using a Mann-Whitney test, to confirm the statistical significance of our results.

6. The authors on line 414 state that "we were still able to detect 12 copies / µL of both targets and observed no significant crosstalk."

However, this statement does not match well with the experimental data in Figure 4C. The leakage signal from the CT switch in Figure 4C(i) appears quite significant and increases with the concentration of the non-cognate NG porA target. In particular, the 10^5 copies of NG porA generates a signal that overlaps with that of the dotted line, which the authors state represents the LoD of NG porA "defined as the mean of three negatives (no target) ± two standard deviations" (+ two standard deviations would be more appropriate since there is only 1 line). It seems quite likely that this crosstalk signal could lead to false positives at higher NG porA levels. Can the authors also explain why the LoD line from the negatives is significantly higher than the signals from the lower NG porA levels (≤ ~10^3 copies)? Why would lower concentrations of NG porA cause suppression of output of AciI?

We believe that some of these concerns stem from our lack of clarity on what the LoD line represents. As discussed above, the LoD line is actually the +2 standard deviation line, not the average. So, while it is true that we observe a slight concentration-specific increase in the red channel upon incubation of the circuit mixture with the NG porA RPA amplicons, suggesting they are able to activate the CT circuit to a small degree, the signal never gains significance above the negative. Thus, while we do agree that leakage from the CT circuit is high (as discussed in the text), the impact of this seems to be more limiting in terms of dynamic range than LoD.

Regarding the apparent suppressive effects, this is a very interesting point, and one that we were also surprised to see. We do not know the reason for this, but we hypothesise that it is not the NG porA amplicons that are suppressing the AcII-expressing circuit, but rather that the expression of the BsaAI is competing with the resources of the PURExpress system, causing suppression of expression of the other circuit. This would seem unlikely, given that the resources are in a large excess, but is not impossible. Unfortunately, given that we used commercial PURE kits for this work, this is not something we could easily control. However, we are now actively investigating this phenomenon by creating our own bespoke PURE systems in which we can carefully control the resources.

7. For line 316: "These optimised Base Gap switch-controlled genes remain stable in solution at both 21 °C and 4 °C for at least eight weeks (Supplementary Figure 5)"

The authors should explicitly state that this test was done using DNA alone and that any enzymes and in vitro transcription/translation components were added later, and presumably stored at low temperature prior to the test. The current language is somewhat ambiguous and could lead readers to assume the system was tested with components that are known to be more susceptible to degradation/denaturation at higher temperatures. It is not unexpected for DNA constructs/primers/plasmids to remain stable for weeks at room temperature.

We apologise for the oversight of your initial comment. **We have now clarified with the following statement in the caption:**

"Incubation times relate only to the DNA circuits; all other components (enzymes, PURExpress, etc) were stored at -20 °C."

8. For the cfDNA experiments, the authors should explain the source of the cfDNA samples and how they were obtained and processed for the assays.

We apologise for the confusion. These triggers were synthetic replicas of the DNA reported by Ma *et al.* These were not clinical samples. **We have added the following text to the Results section:**

"Titration of synthetic analogues of both mutant (T790M) and wild-type triggers against this SGC confirmed the specificity of the switch (**Figure 5D**$_{i}$, **Supplementary Figure 17**)."

We have also added the following text to the caption:

"All data were obtained using synthetic analogues of the mutants described by Ma *et al.*[59]"

9. For Figure 5D(i), the authors have chosen to retain their claim that the detection limit of the assay for T790M DNA is 10 fM.

The 100 fM (0.1 pM) measurements clearly overlap with the LoD line on the plot. If the true LoD is 10 fM, then the 100 fM data should also be clearly above the LoD line. Also, following the earlier comment, the dotted line should not be used for determination of LoD because it does not seem to reflect the variance in the negative measurements.

We apologise for this oversight. You are entirely correct. However, when analysing the data, we noticed that the overlap of 100 fM was caused by a potentially outlying data point. To confirm beyond doubt that this test is capable of detecting 10 fM without amplification, we have repeated this analysis with n = 6 replicates and performed a Mann-Whitney test to

confirm that the 10 fM results is statistically significant from the negative. The data is significant (p < 0.01) according to the Mann-Whitney test, and all replicates lay above the +2 standard deviation line. We have now updated the figure and caption with these new data.

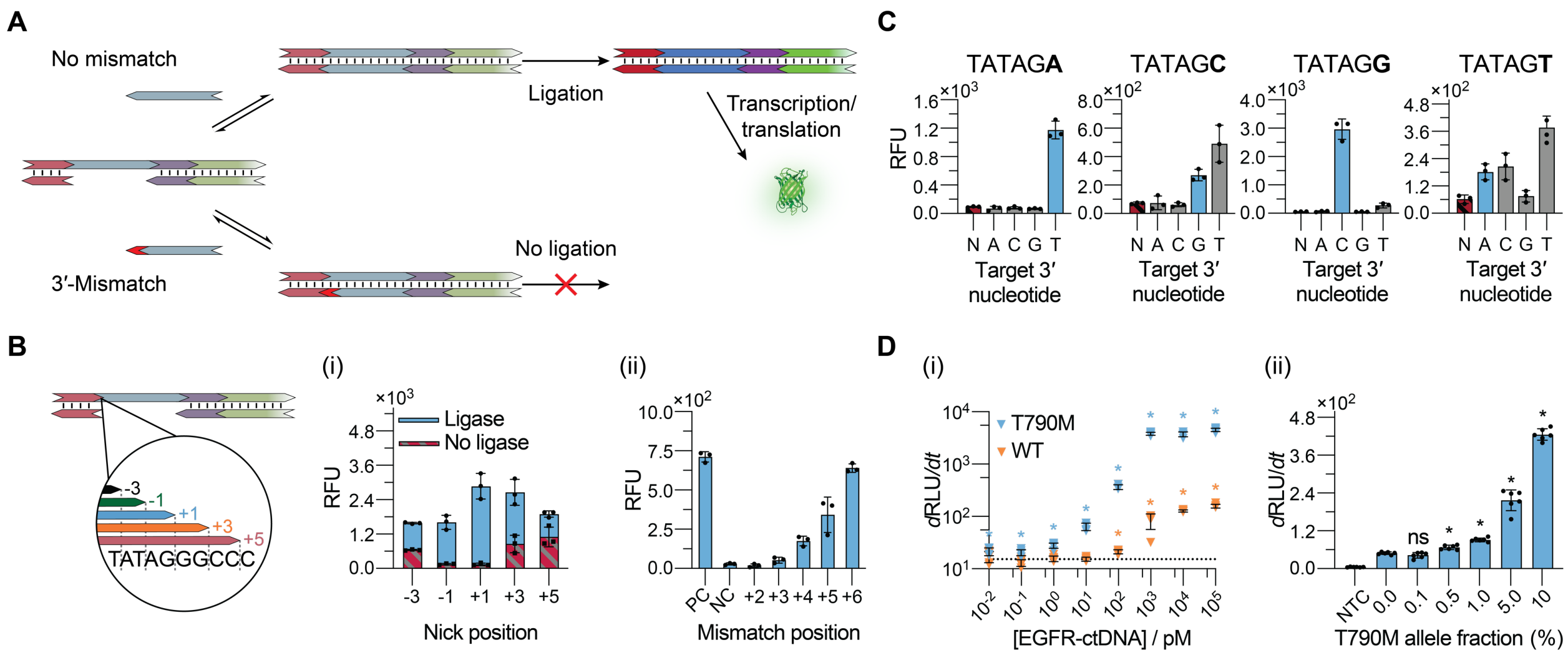

Figure 5. SNV detection using Base Gap switches. A) A schematic describing base mismatch discrimination using Base Gap switches. Mismatches at the 3′ end of the target prevents ligation to the promoter region (red). In the absence of ligation, RNAP cannot bypass the Base Gap and the expression cascade is muted. **B)** (i) mNeonGreen fluorescence as a function of ligation (nick) position, with and without ligase. The positions are numbered relative to the transcriptional start position for the T7 promoter (G,+1). (ii) mNeonGreen fluorescence as a function of mismatch position. A polyguanine (GGGGG) region was introduced directly following the nick site, with triggers containing a T mismatch in the assigned position. Positive control (PC) is a fully matched trigger. Negative control (NC) is no trigger. The greatest ligase dependence was observed with the nick at the +1 position, and high mismatch fidelity was observed with the mismatch placed in the 1-3 position. **C)** Relative mNeonGreen fluorescence values obtained with different base pair mismatches between the switch and the trigger. The bolded letters above indicate the nucleotide at the +2 position of the Base Gap switch. The x-axis indicates the nucleotide at the 3′ end of the trigger. N = negative (no target). For each graph, the bar representing the complementary base is highlighted in blue. Switches with A and G nucleotides at the +2 position were highly sensitive to base pair mismatches. **D** (i) NanoLuc Luciferase (Nluc) luminescence as a function of mutant EGFR cfDNA concentration. The Base Gap switch was specific to the T790M (C-T) mutation of EGFR. 10 fM of T790M DNA could be detected whereas 100 pM of wildtype DNA was required to achieve significance. The dotted line represents the +2 standard deviation above the mean of the non-template control (negative). (ii) Relative Nluc luminescence as a function of T790M allele fraction (fraction of T790M cfDNA spiked into WT cfDNA). The total DNA trigger concentration was 1 nM. We detected allele fractions as low as 0.5%. All plots with error bars represent the mean ± one standard deviation. Statistical significance was determined using a Mann-Whitney test (* = $p < 0.01$, ns = not significant). All data were obtained using synthetic analogues of the mutants described by Ma *et al.*[59]

Interestingly, we note that with n = 6, we observed significance from the negative with the WT trigger at 100 pM, whereas previously significance above the LoD was observed at 1000 pM. We have updated the caption to reflect this. However, this does not impact our statements on specificity, as this is demonstrated in Figure Dii.

10. For Figure 5D(ii), how much total DNA was added to the reactions?

For these experiments, the total trigger DNA concentration is 1 nM. **This has been updated in the caption:**

"The total DNA trigger concentration was 1 nM."

11. For Supplementary Figure 10B, more information on the experiment should be provided.

Were the triggers supplied as ssDNA or dsDNA? Did they have 5' phosphate groups? Was ligase used in the reactions? The absence of a strong signal seems to contradict other experimental data in the paper. In orthogonality study using 50-nt triggers, a non-cognate trigger with 90% sequence match to the cognate trigger demonstrated 30% crosstalk. Thus, two 30-nt regions with 100% sequence match should be able to hybridize to the template and be ligated. Moreover, in Figure 1C, 3' elongated and 5' elongated triggers can still generate gene products, which demonstrates that T7 RNAP can continue transcribing even when the templates are not base paired. The authors should describe in the Discussion how the results in Supplementary FIgure 10 can be reconciled with the potentially contradictory data from other experiments. Sequences for the experiments in Supplementary Figure 10B should also be provided. They do not seem to be present in the Supplementary Tables spreadsheet or on the chemRxiv version of the tables (https://doi.org/10.26434/chemrxiv.15001046/v1).

These experiments were set up under the same conditions as all the others. These were ssDNA triggers, with no 5` phosphate groups. As shown in **Figure 1C,** phosphate groups at the 5` of the triggers did not significantly impact expression yields. Ligase was used in the reactions.

While we were surprised by quite how well these experiments worked, we do not believe they are contradictory. While the triggers used in **Supplementary Figure 10B** have a 30 nt overlap, this is not 100% sequence match. The trigger length here is 95 nt, so the total similarity is 63%, thus much lower than the data from **Figure 4**. We believe that ligation is likely occurring, but transcription is simply halted by the larger degree of mismatched base pairing. Moreover, as demonstrated in **Figure 2**, it is likely that overall expression rates are governed not just by the degree of hybridization, but other parameters such as mRNA hairpin stability. Thus, comparing the two data sets based just on degree of sequence similarity may be too simplistic.

We have added the following text to the Discussion section:

"Importantly, we demonstrated that triggers with a high degree of paired identity at the termini (63%) did not cause significant activation of Base Gap switches (**Figure 10**). This suggests that primer dimers will not lead to increased assay background, provided they are

designed well and the amplicons are of sufficient length to maintain an overall paired identity.”

We have also added the trigger sequences used here to Supplementary Tables 5 and 6:

TBS_length_95nt	5'-ATTGGGTTGTGAATAGGCGCCAAAAACAGCCAGCCTTCCTAAGATGTCTTGCCGGTTGGTCGTCCATACTGCAGAGGACGAGAACTGAATGTGTA-3'
TBS_length_95nt_5ntoverlap	5'-ATTGGATTCCACCAGTAGTACACAGATTAAGTCGTCTCCAGCCAACATTTGGTGAAATCAGATTCCCGGAACAGGGTATAAGCATCGAGGGTGTA-3'
TBS_length_95nt_15ntoverlap	5'-ATTGGGTTGTGAATATAGTACACAGATTAATCGTCTCCAGCCAACATTTGGTGAAATCAGATTCCCGGAACAGGGTATAAAGAACTGAATGTGTA-3'
TBS_length_95nt_30ntoverlap	5'-ATTGGGTTGTGAATAGGCGCCAAAAACAGCTCGTCTCCAGCCAACATTTGGTGAAATCAGATTCCATACTGCAGAGGACGAGAACTGAATGTGTA-3'

Figure S10

Panel	Encoder	Switch	Trigger
		TBS_length_95nt	TBS_length_95nt
		TBS_length_95nt	TBS_length_95nt_5ntoverlap
		TBS_length_95nt	TBS_length_95nt_15ntoverlap
A	nLuc	TBS_length_95nt	TBS_length_95nt_30ntoverlap

12. Methods section: How long does the ligation step before the IVTT reaction take?

We perform the ligation reaction *in situ*, rather than performing pre-ligation. **We have the following text in the Results section:**

“Extended pre-incubation with T4 ligase did not substantially improve expression yields (**Supplementary Figure 4$_{i}$**), nor did increasing the ligase concentration (**Supplementary Figure 4$_{ii}$**). Accordingly, moving forward, we performed ligation *in situ* to reduce the number of assay steps”

13. Line 186: "The RBS scores for both start positions encoding a functional protein (+85, +87)": Are these positions correct?

Yes, +85 corresponds to the AUG codon. As is often the case, the +87 position (omitting the starting methionine residue) often also has a reasonably high RBS score. We chose to include this, as it would still produce a functional protein.

14. In Supplementary Table 1, none of the RPA primers are shown as having 5' phosphate groups. Are these present in the primers? If not, how is transcription affected by the presence of a nick in the template strand?

This is true; we do not have 5` phosphate groups here. We showed in **Figure 1C** that sealing the gap between the trigger and the RBS did not significantly impact expression yields, and thus moving forwards we opted not to include them. However, it is possible that this could improve the expression yields for dsDNA triggers, as it would help push the reaction in our favour. This is something we are currently exploring.

We have the following text in the Results section:

“Intriguingly, the presence of a nick on the distal end of the trigger–switch interface, between the TBS and the RBS, did not impact expression, and sealing this nick is not necessary (**Figure 1C**, column 9).”

Response to Reviewer 3

Thank you once again for your feedback. Regarding your comment about the supplementary data/tables, I will request that the editor send these directly to you. We have uploaded them each time, so I am not sure why they are not being made available to you. Below is a point-by-point response to your comments.

1. The author's use of the dashed line to make LOD determinations is not sufficiently transparent and does not allow readers to see how reasonable the LOD measurements are.

To avoid this issue, the authors must include the data points they measured for their NTC experiments on their plots rather than having the dashed line on its own. Simply including this important information would remove the uncertainty surrounding their LOD determinations. Indeed, Figure 3C already includes the NTC measurements and it is clearly helpful in identifying the false-negative measurements. The NTC measurements should be included at minimum in Figure 3B, Figure 4C(i and ii), and Figure 5D(i). Also, individual data points should be spread out more laterally so that it is easier for readers to discern data variability. This is particularly a problem for Figure 5D(i) and Figure 3B, even though the points are spread out laterally for a very similar plot in Figure 4C.

We have now added the data points for the NTCs to Figure 3B, Figure 4C, and Figure 5D(i). Inclusion of these NTCs clearly demonstrates the significance of our data. We have also spaced the data points in these figures further apart to improve legibility.

Figure 3

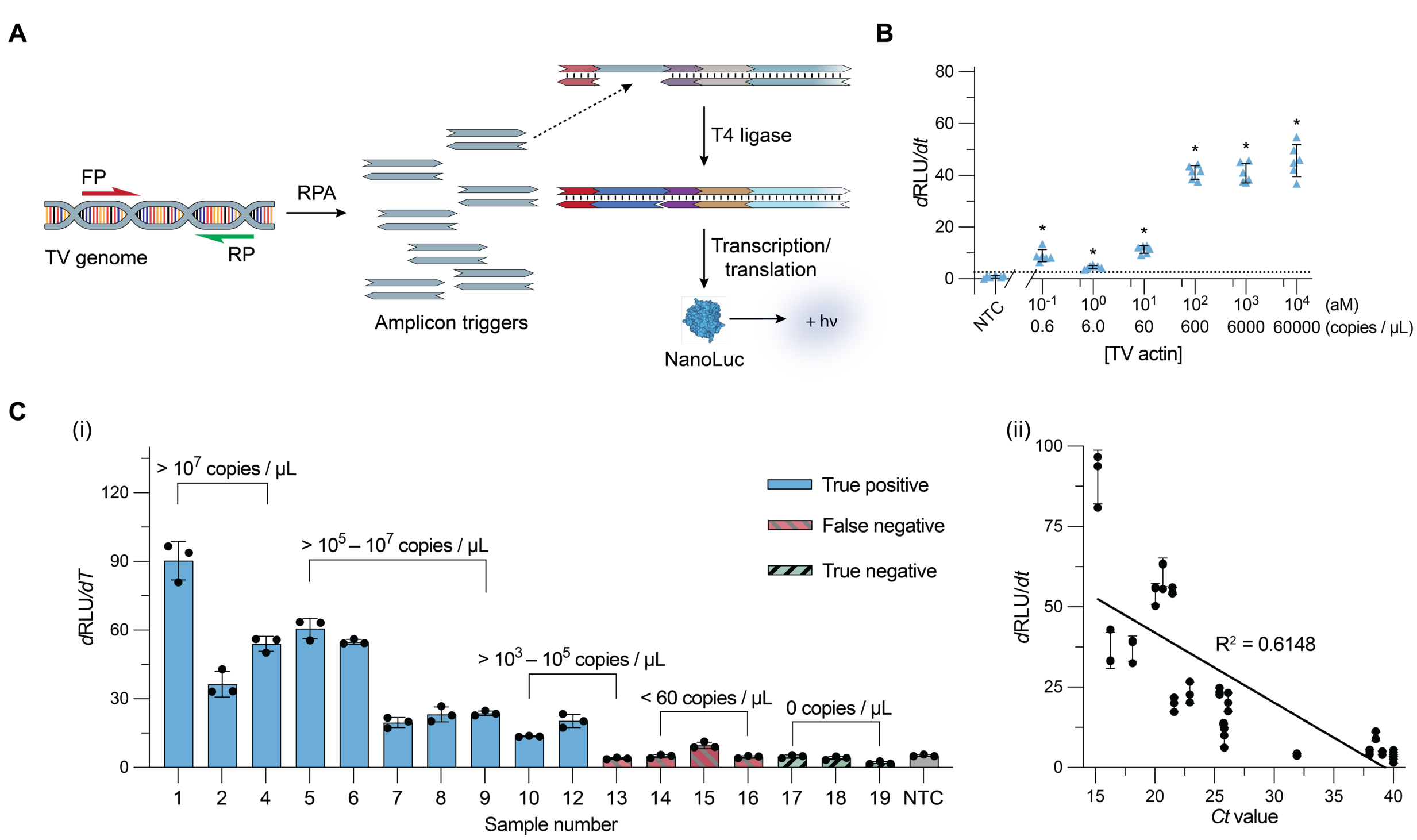

Figure 4

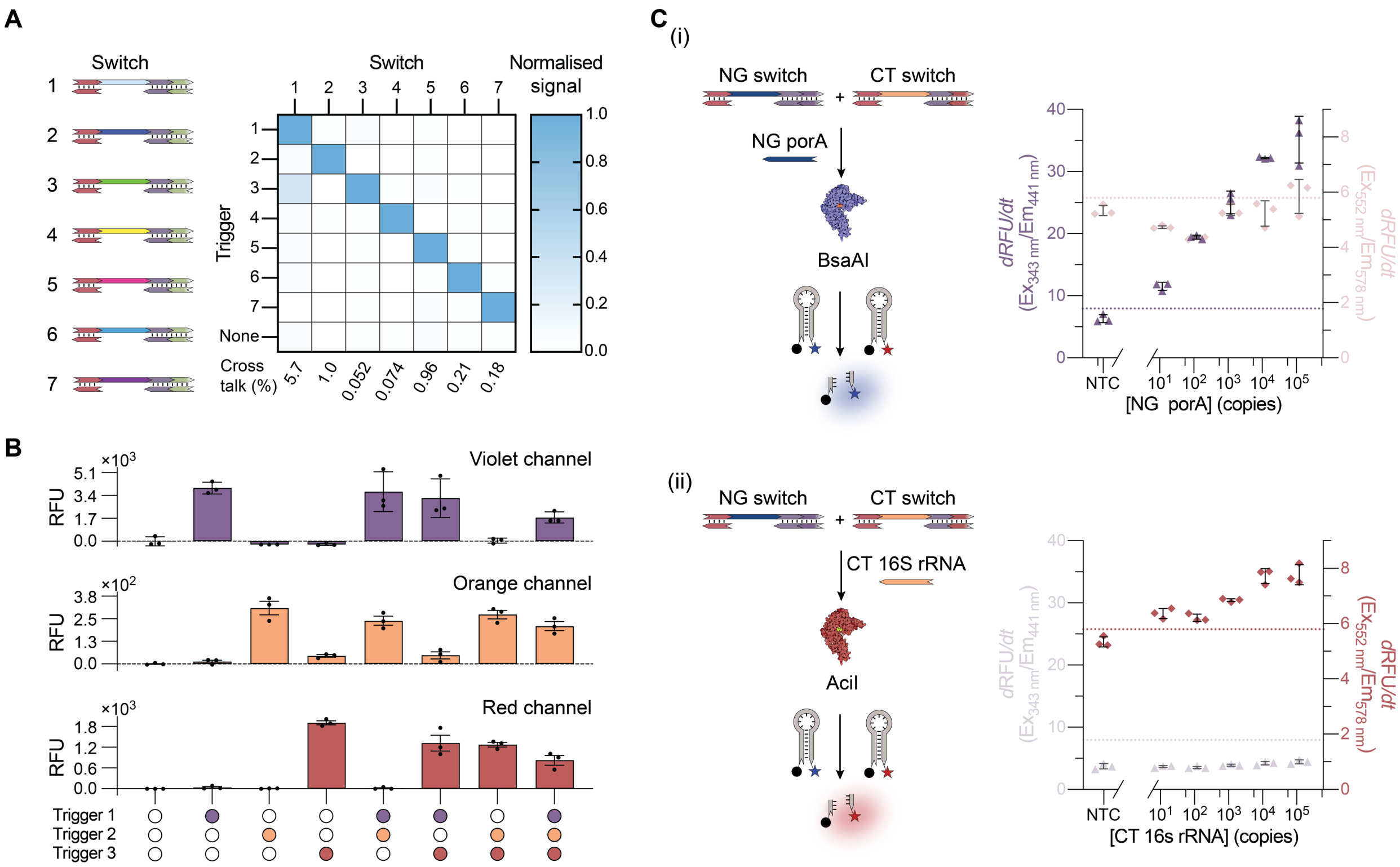

Figure 5

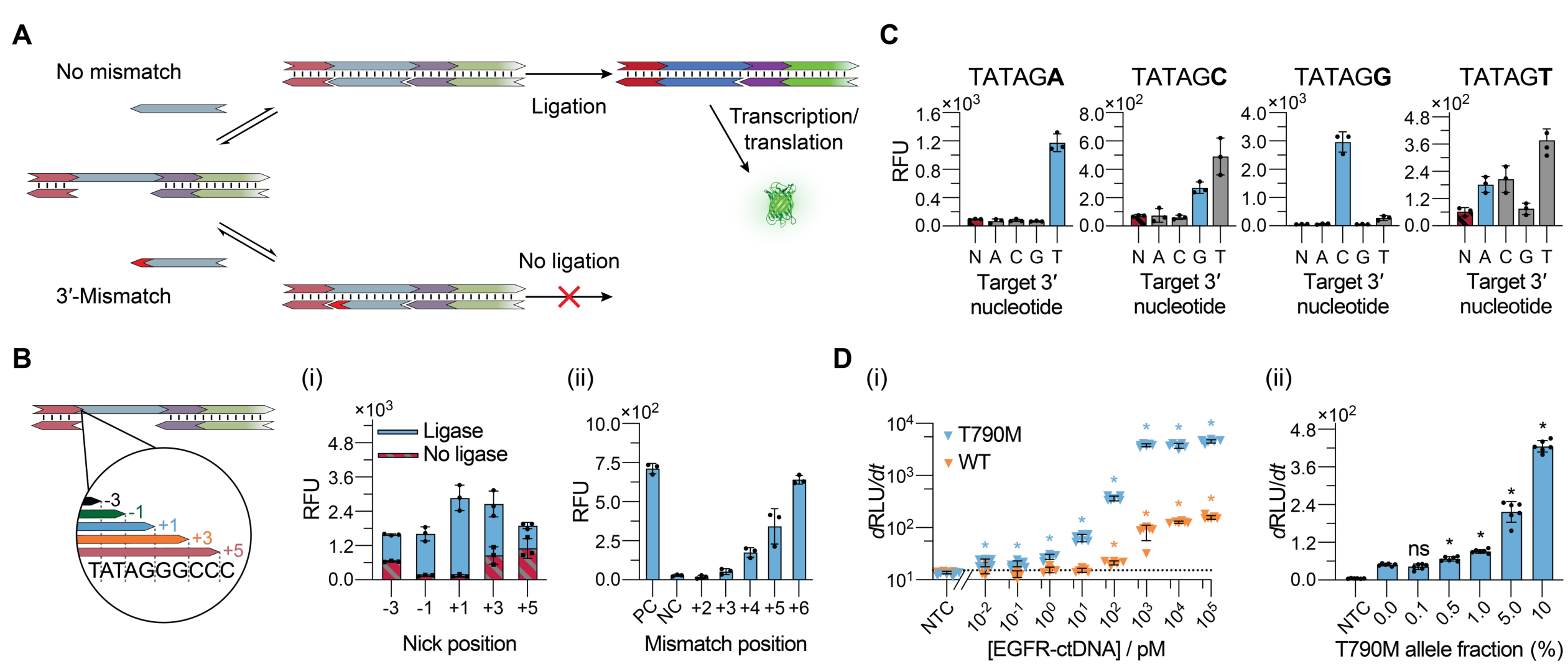

2. Regarding the revision to the stability testing description (line 285-286), it is inappropriate to only mention in the caption of the supplementary figure that enzymes were not involved in stability testing.

Readers could assume from the main text that room-temperature stability was actually validated for the sensors. The main text must state that all other components (enzymes, PURExpress, etc) were stored at -20 °C prior to use in the experiment.

We have rectified this with the following statement in the Results:

"These optimised Base Gap switch-controlled genes remain stable at both 21 °C and 4 °C for at least eight weeks (**Supplementary Figure 5**), though it is worth noting that other components (PURExpress, Quick Ligase) were stored at −20 °C until use. We are currently conducting further experiments to determine the stability of lyophilised mixtures."

3. The use of the term "in situ" regarding the ligation step is unclear. In a biological context, this usually means in a biological sample or specimen. Language like a one-pot or all-in-one ligation/IVTT reaction would be clearer.

We have amended the Results section as clarification:

"Accordingly, moving forward, we performed ligation alongside IVTT ("one-pot" ligation/IVTT) to reduce the number of assay steps."

4. Use of positions +85 and +87 for RBS calculator translation start site is still perplexing. +85 and +87 only differ by 2 nt so they would not be in the same reading frame.

Thank you very much for spotting this. This should be positions +85 and +88.

We have amended this in the Methods section:

"The RBS scores for both start positions encoding a functional protein (+85, +88) were summed and used in the subsequent analyses."

5. The results from Supplementary Figure 10B make more sense with the sequence information and knowing there is no 5' phosphate and there is a nick in the template. However, it seems unlikely that transcriptional termination is occurring because of mismatched base pairing since T7 RNAP can transcribe through DNA duplexes.

Furthermore, it is well known that T7 RNAP can efficiently transcribe on ssDNA after transcription initiation from a dsDNA promoter, so termination in the mismatched region is unlikely. It seems more likely that the observed behavior is from the effect of the transcriptional bubble from the active RNAP. The bubble draws the distal portion (5' end) of the target away from the complementary portion of the sense strand. With the mismatched middle region of the target not bound to the sense strand, there will probably be a greater distance that the RNAP must jump for template switching, which lowers transcriptional efficiency into the downstream region.

The exact mechanisms of how these gaps slow down (or terminate) transcription remains an active area of study in our lab. While several studies have shown that RNAP can transcribe from ssDNA, they have all been performed on single-stranded template strands. Our single-stranded region is on the coding strand. I agree that the mismatches in the middle likely increase the “jumping” distance for template switching, which can result in early termination. As a follow-up study, we plan to perform both transcriptomics and proteomics to further elucidate the mechanisms underpinning Base Gap switches.

6. It would be very helpful to readers if the authors could clarify their figures. Some marker to indicate if a strand is phosphorylated would be useful, and some difference in the diagram to indicate whether strands are ligated or nicked would avoid confusion. For instance, in Figure 3A, the diagram does not make it clear that the distal portion (5' end) of the target is not ligated while the other end would be. At present, readers would be forced to look into the supplementary tables to get most of this essential information.

These are helpful suggestions. We have added white space to indicate a nick in the strand in the schematic diagrams throughout the manuscript. We have opted not to include any additional colour to indicate phosphorylation, as we feel this could be mistaken for the markers we use to indicate SNPs and cause confusion. However, in Figure 1A we have clearly indicated the phosphorylation site with a circle.

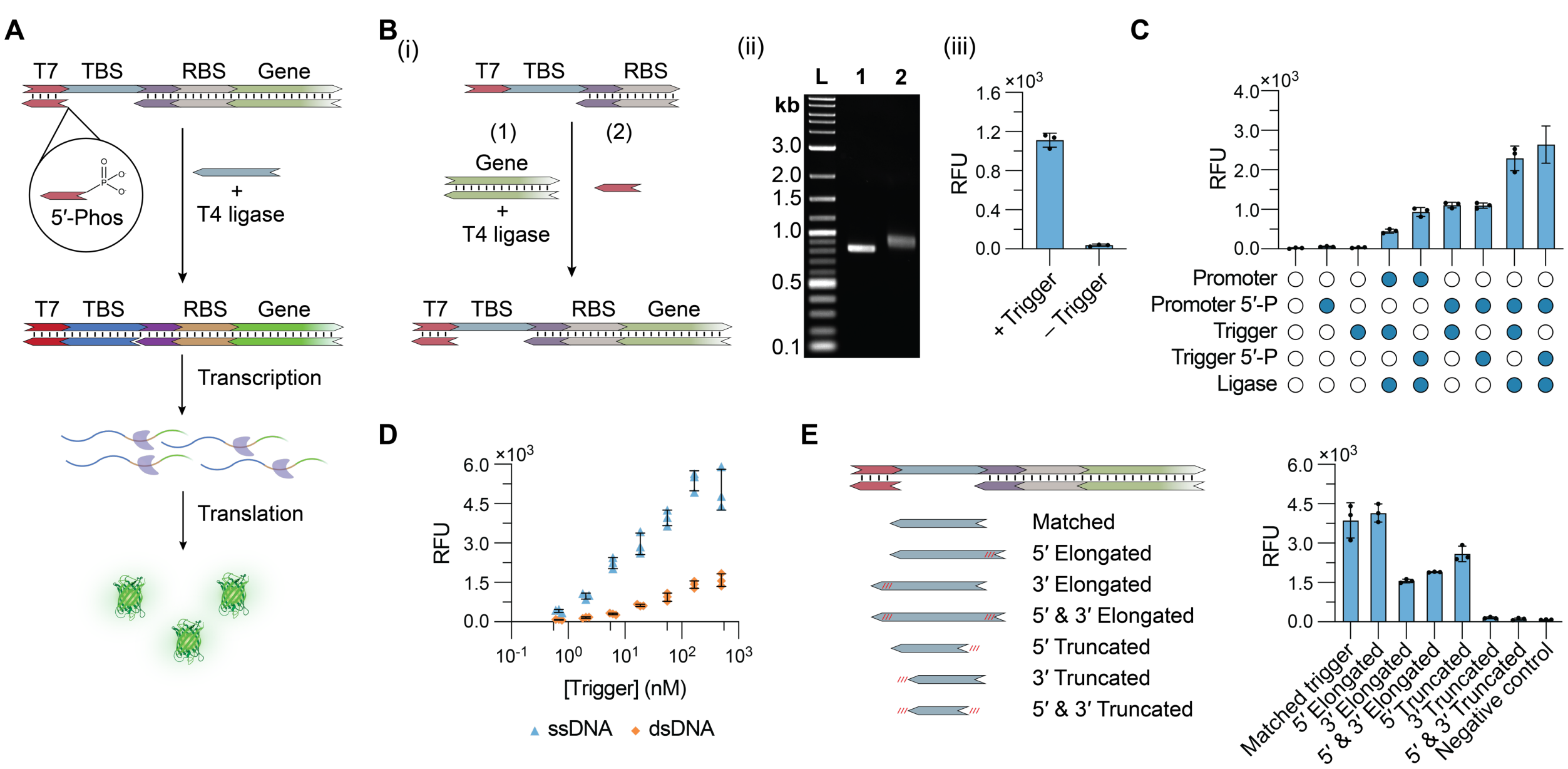

We have also added the following to the caption of Figure 1 as clarification:

“Ligation between the trigger and the promoter region significantly enhances protein expression. Ligation between the trigger and the RBS region does not significantly enhance protein expression.”

7. In Figure 3C(i), the size of the error bars do not seem to match the spread of the individual data points (e.g. Samples 1-5, 8).

Thank you for noticing this. These error bars were plotted as 95% confidence intervals rather than SDs by mistake. **This has now been rectified:**

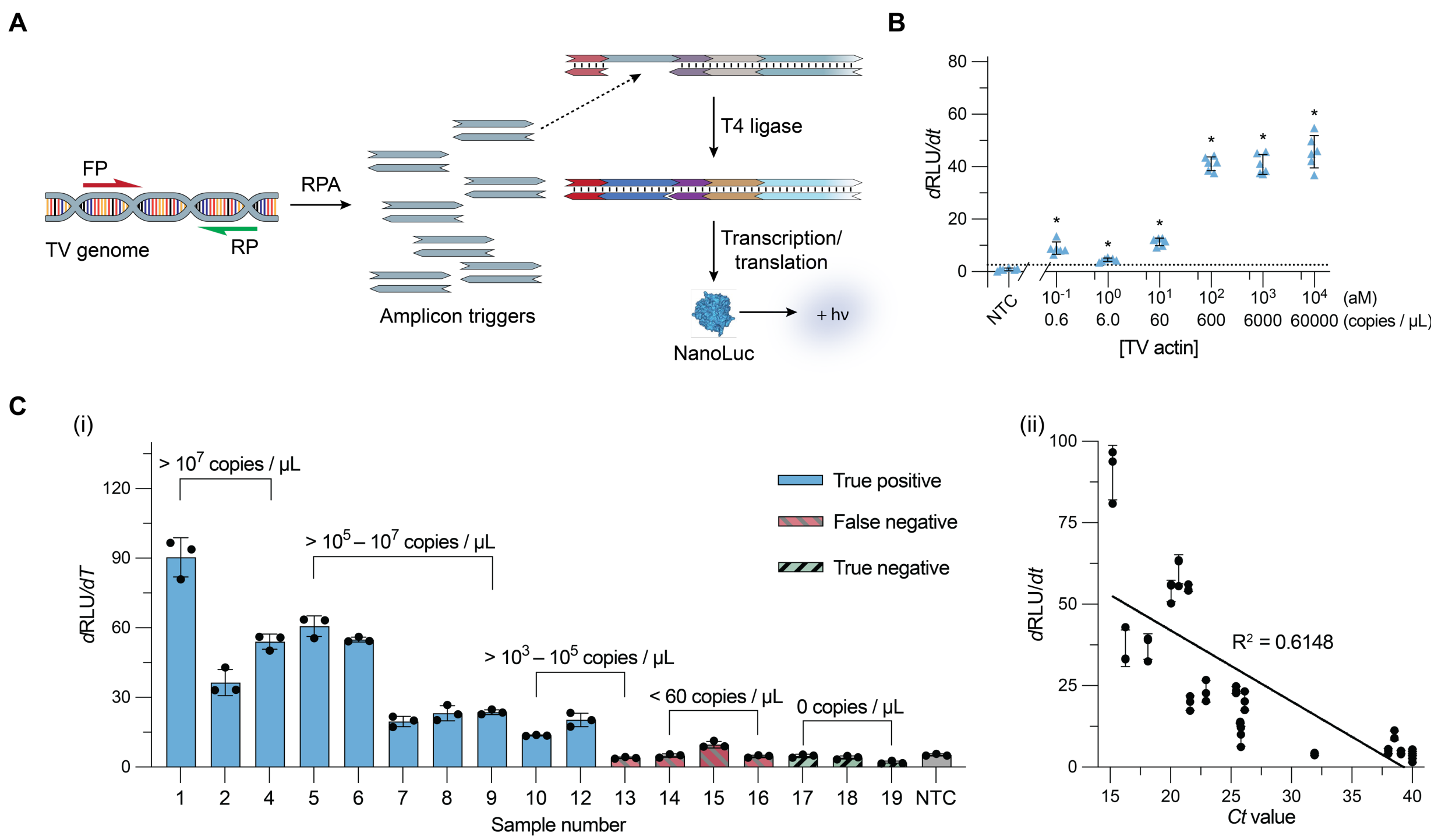

Supplement: Supplementary file 3 [file 44460_2026_134_MOESM3_ESM.pdf]
